# Autophagic flux blockage by accumulation of weakly basic tenovins leads to elimination of B-Raf mutant tumour cells that survive vemurafenib

**DOI:** 10.1371/journal.pone.0195956

**Published:** 2018-04-23

**Authors:** Marcus J. G. W. Ladds, Andrés Pastor-Fernández, Gergana Popova, Ingeborg M. M. van Leeuwen, Kai Er Eng, Catherine J. Drummond, Lars Johansson, Richard Svensson, Nicholas J. Westwood, Anna R. McCarthy, Fredrik Tholander, Mihaela Popa, David P. Lane, Emmet McCormack, Gerald M. McInerney, Ravi Bhatia, Sonia Laín

**Affiliations:** 1 Department of Microbiology, Tumor and Cell Biology, Karolinska Institutet, Stockholm, Sweden; 2 Chemical Biology Consortium Sweden, Department of Medical Biochemistry and Biophysics, Science for Life Laboratory, Division of Translational Medicine and Chemical Biology, Karolinska Institutet, Stockholm, Sweden; 3 Department of Pharmacy, Uppsala University Drug Optimization and Pharmaceutical Profiling Platform (UDOPP), Uppsala University, Uppsala, Sweden; 4 School of Chemistry and Biomedical Science Research Complex, University of St. Andrews and EaStCHEM, St Andrews, Fife, Scotland, United Kingdom; 5 Department of Medical Biochemistry and Biophysics, Karolinska Institutet, Stockholm, Sweden; 6 Department of Clinical Science, University of Bergen, Bergen, Norway; 7 Department of Internal Medicine, Hematology Section, Haukeland University Hospital, Bergen, Norway; 8 Department of Hematology and Oncology, University of Alabama, Birmingham, Alabama, United States of America; Univerzitet u Beogradu, SERBIA

## Abstract

Tenovin-6 is the most studied member of a family of small molecules with antitumour activity *in vivo*. Previously, it has been determined that part of the effects of tenovin-6 associate with its ability to inhibit SirT1 and activate p53. However, tenovin-6 has also been shown to modulate autophagic flux. Here we show that blockage of autophagic flux occurs in a variety of cell lines in response to certain tenovins, that autophagy blockage occurs regardless of the effect of tenovins on SirT1 or p53, and that this blockage is dependent on the aliphatic tertiary amine side chain of these molecules. Additionally, we evaluate the contribution of this tertiary amine to the elimination of proliferating melanoma cells in culture. We also demonstrate that the presence of the tertiary amine is sufficient to lead to death of tumour cells arrested in G1 phase following vemurafenib treatment. We conclude that blockage of autophagic flux by tenovins is necessary to eliminate melanoma cells that survive B-Raf inhibition and achieve total tumour cell kill and that autophagy blockage can be achieved at a lower concentration than by chloroquine. This observation is of great relevance as relapse and resistance are frequently observed in cancer patients treated with B-Raf inhibitors.

## Introduction

Tenovin-6 is a small molecule identified as an activator of p53-dependent transcription in a cell-based screen of 30,000 compounds [1]. *In vivo*, tenovin-6 reduces the rate of growth of xenograft tumours originating from ARN8 melanoma cells implanted in SCID mice [1]. Furthermore, tenovin-6 destabilises Myc proteins and reduces tumour growth in a N-Myc-driven tumour model [2,3]. It has been shown that tenovin-6 can induce apoptosis in leukaemic stem cells taken from chronic myelogenous leukaemia (CML) patient samples and achieve cure in murine CML models together with the Bcr-Abl tyrosine kinase inhibitor imatinib [4,5]. Additionally, tenovin-6 has been shown to synergise with the tyrosine-kinase inhibitor AC220 and significantly reduce acute myeloid leukaemic stem cells and lead to apoptosis of FLT3-ITD+ cells [6,7]. This remarkable anti-leukaemic stem cell effect of tenovin-6 is proposed, at least in part, to be a result of its ability to inhibit SirT1 protein deacetylase activity and subsequent activation of p53. Indeed, SirT1 deacetylates lysine residues of p53 and promotes its subsequent ubiquitination and degradation [8,9].

There are 76,380 new cases of melanoma predicted in the United States alone in 2017 [10], which together with its aggressive and invasive nature, makes it a challenging cancer to treat. Around 50% of melanomas possess activating mutations in B-Raf, a key component of the Ras-Raf-MEK-ERK pathway responsible for converting extracellular signals into expression of genes responsible for proliferation, survival and differentiation [11]. This led to the development of targeted therapeutics against B-Raf kinase activity [12]. One such inhibitor is vemurafenib, a drug currently used in the clinic to treat melanoma [13]. Vemurafenib, however, may be insufficient to achieve cure as a single agent in mutant B-Raf driven melanomas [14]. Coupled with this, trials of vemurafenib with the immune stimulatory agent ipilimumab, a human monoclonal antibody targeting CTLA-4, have resulted in hepatotoxicity [15]. Finally, combination of vemurafenib with anti-PD1 therapies such as nivolumab has resulted in cutaneous and neurologic toxicities [16]. This inability of vemurafenib to synergise with ipilimumab due to treatment-limiting toxicity makes finding another non-genotoxic therapy for melanoma to combine with vemurafenib an important need. One potential pathway to exploit is autophagy.

Autophagy is the process of self-ingestion of defective macromolecules and organelles. It provides an efficient mechanism for detoxification as well as a means for survival during nutrient starvation [17]. There are three phenotypically distinct forms of autophagy: macro-autophagy, micro-autophagy and chaperone-mediated autophagy [17,18]. In the context of this paper, macro-autophagy–the process of degrading cytoplasmic contents in the lysosome via fusion to the intermediary double membrane–bound autophagosome [17,18] is henceforth referred to solely as autophagy. The possibility that modulation of autophagic flux is a potential cancer therapeutic has increasingly gained acceptance [19]. Indeed, there are a rising number of publications citing chloroquine (CQ) co-treatment with current cytotoxic and targeted therapeutics to increase tumour cell kill as a proof of principle [19–22]. In particular hydroxychloroquine (HCQ), a less toxic analogue of CQ, is in clinical trials in combination with imatinib [23] as well as 13 other current clinical trials listed on clinicaltrials.gov. Protonation of nitrogen-containing moieties in CQ, in particular its aliphatic tertiary amine, is thought to trap the molecule in acidic organelles, such as the lysosome, causing alkalinisation [24–27]. This alkalinisation impairs lysosomal hydrolases that depend upon low pH for their activity, and affects the fusion of the autophagosome and lysosome [28,29].

Previously it has been reported that tenovin-6 is capable of perturbing autophagy [30–32]. It has been speculated that the inhibitory effect of tenovins on SirT1 is responsible for the perturbation of autophagy, though this was called into doubt in a recent publication [33]. However, as tenovin-6, much like chloroquine, possesses a side chain with an aliphatic tertiary amine capable of acting as a proton acceptor, we investigated whether these moieties may contribute to blocking autophagic flux in cells through a similar mechanism to chloroquine. Equally, due to the activity of tenovin-6 against tumour cells, we investigated whether the tenovins were capable of achieving total tumour kill in melanoma cultures, whether the tenovins were capable of killing cells arrested by vemurafenib treatment, and whether the effect of tenovins on autophagic flux contributed to their killing effect on tumour cells.

## Materials and methods

### Compounds and reagents

All antibodies were obtained from sources as described in the figure legends. CQ diphosphate salt was obtained from Sigma-Aldrich (St. Louis, MO, USA C6628). CQ diphosphate salt was made up as 100 mM stock in 1× PBS (HyClone, Thermo Scientific, Waltham, MA, USA #SH30256) and freshly made each experiment. Tenovin-1 was purchased from Cayman (Ann Arbor, MI, USA #13085). Vemurafenib was purchased from Selleck Chemicals (Houston, TX, USA #S1267). Vemurafenib stocks were prepared as 40 mM in DMSO (Sigma-Aldrich #D8418) and stored at–20°C. The synthesis of tenovins 6, 30a, 30b, 30d, 30j, 30k, 30n, 33 and 39 was described previously [34]. The synthesis of tenovins D1 and D3 was described previously [35]. The synthesis of tenovins 39-OH, 50, 50-OH and 51 is described in supplemental methods. All tenovin compounds were prepared as 40 mM or 60 mM stocks in DMSO (Sigma-Aldrich #D8418) and stored at–20°C.

### Cell lines and growth conditions

ARN8 cells are derived from the parental A375 human melanoma cell line by stably transfecting the pRGCΔfos-lacZ p53-dependent reporter construct and pSV_2_ neo as described previously [36]. HOS-EGFP-LC3 cells are described previously [37]. Human normal dermal fibroblasts (HNDF) were purchased from PromoCell (Heidelberg, Germany #C-12300). MDA-MB468 human breast carcinoma cells (#HTB-132), HT144 melanoma cells (#HTB-63), Colo829 melanoma cells (#CRL-1974), H1299 metastatic lung cancer cells (#CRL-5803) and A375 melanoma cells (#CRL-1619) were all purchased from ATCC (Manassas, VA, USA). SK-Mel-28 melanoma cells were a kind gift from Stig Linder and Johan Hansson (Karolinska Institute). All cell culture medium was supplemented with 10% fetal bovine serum (FBS) v/v (Hyclone #SV30160) and 100 U mL^-1^ penicillin/streptomycin (Hyclone #SV30010) unless otherwise specified. HNDF, MDA-MB468, ARN8 and A375 cells were cultured in high glucose DMEM (Hyclone #SH30243). Colo829 and H1299 cells were grown in RPMI-1640 (Hyclone #SH30027.01). SK-Mel-28 were grown in MEME (Sigma #51416C). HT144 cells were grown in McCoy’s medium (Sigma #M8403). All cells were grown at 37°C in atmospheric O_2_, 5% CO_2_ and high humidity. Passaging of cells was conducted using trypsin/EDTA (Sigma-Aldrich #T4174) detachment. All cells were counted using a Bürker cell counting chamber. All cells were tested for mycoplasma using a commercially available kit (MycoAlert, Lonza Biosciences LT07-418)

### Sulforhodamine B viability assay (SRB)

**Fig 1:**
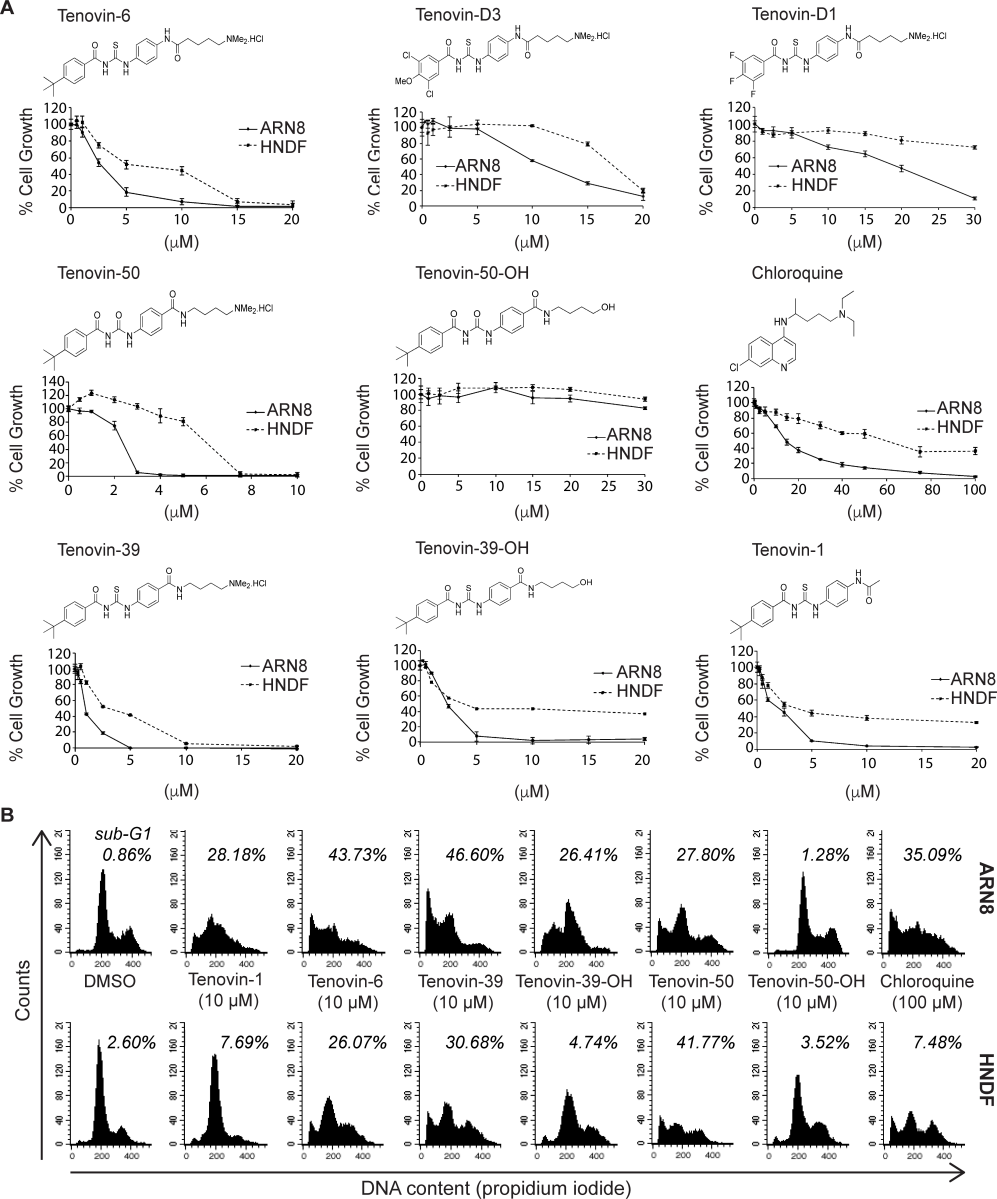
Structure activity relationship study of tenovin-6 analogues. (A) Sulforhodamine B (SRB) assays and structures for indicated compounds illustrating the ability to reduce cell growth in ARN8 human melanoma cells or in human normal dermal fibroblast (HNDF) cells with treatment for 72 hours prior to staining with SRB. (B) Flow cytometric analysis of cell cycle progression for ARN8 human melanoma and HNDF cells treated with the indicated compounds for 48 hours prior to fixation and staining with propidium iodide and analysis by flow cytometry.

For Fig 1 and S1 Fig, cells were seeded in a 96-well plate at a density of 500 cells per well (ARN8) or 2000 cells per well (HNDF) in a volume of 100 μL fully supplemented growth medium and incubated as described above for 24 hours. Cells were checked for adherence and treated with tenovins in DMSO or chloroquine in PBS. The final percentage of DMSO per well was 0.2%. Cells were incubated for either 48 hours or 72 hours with compounds. Following incubation, media was removed from the cells and replaced by 150 μL of 1× sterile PBS and 50 μL of 40% w/v trichloroacetic acid (Sigma-Aldrich #T9159) in dH_2_O and incubated at 4°C for one hour to fix the cells. The plate was then washed with three changes of water with all liquid allowed to drain from the plate. 50 μL of 0.4% w/v sulforhodamine B (Sigma-Aldrich #230162) with 1% v/v acetic acid (Sigma-Aldrich #320099) in dH_2_O was added to each well and incubated for 30 minutes. Excess dye was washed out with three changes of 1% v/v acetic acid in dH_2_O. The remaining dye was solubilised in 100 μL of 10 mM un-buffered Tris-base (Sigma-Aldrich #93362) per well and absorbance was read at 570 nm on a spectrophotometer.

### Western blotting

**Fig 2:**
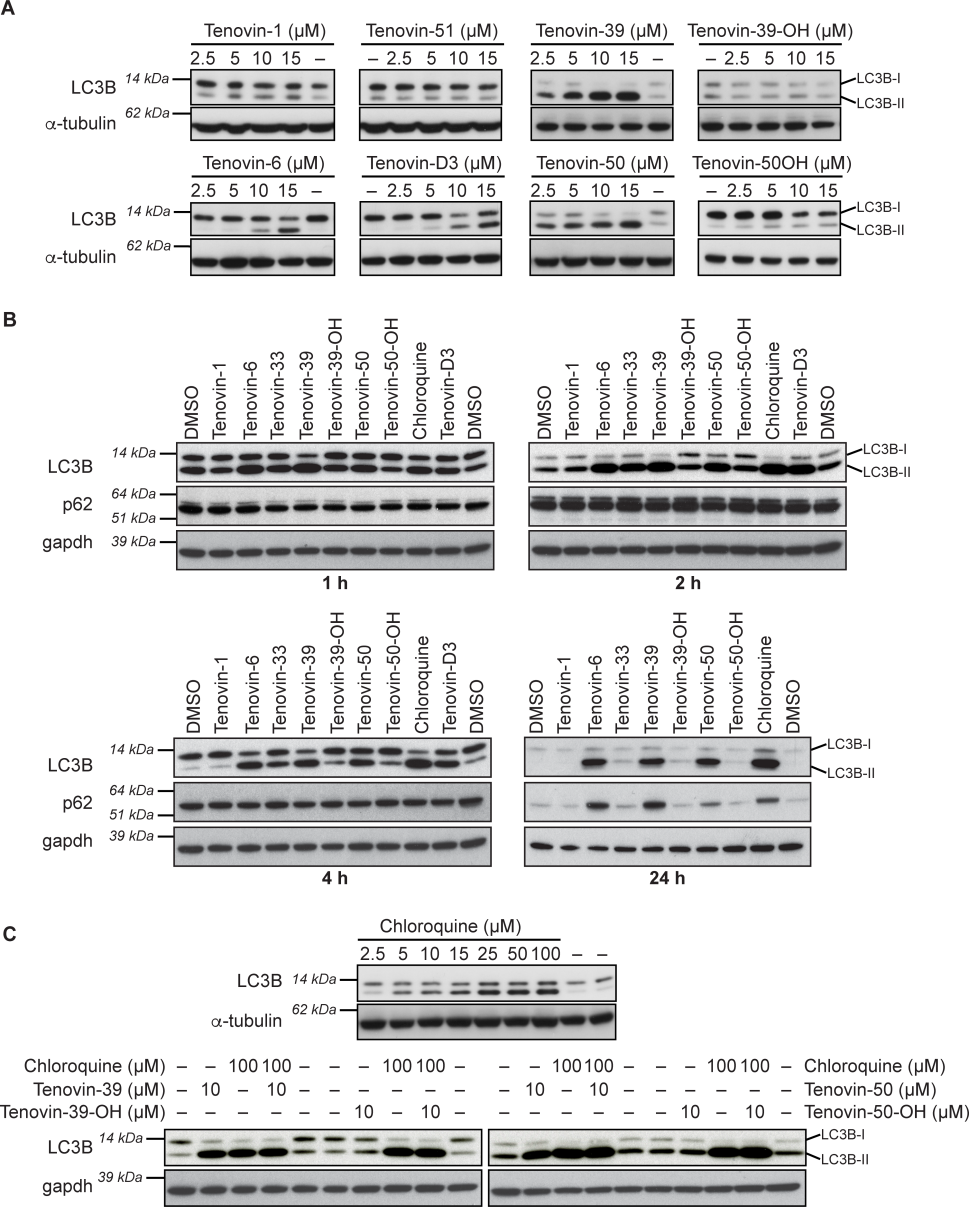
Induction of LC3B is correlated with the presence of an aliphatic tertiary amine. (A) MDA-MB468 human breast cancer cells treated with indicated doses of the stated compounds or vehicle for six hours with LC3B and alpha-tubulin detected by western blot. See details of the presence or absence of the aliphatic tertiary amine in Table 1. (B) MDA-MB468 human breast cancer cells treated with indicated compounds at 10 μM for indicated times with LC3B, p62 and alpha-tubulin detected by western blot. (C) MDA-MB468 human breast cancer cells treated with indicated doses of the stated compounds with or without chloroquine for six hours with LC3B and alpha-tubulin detected by western blot.

Cells were seeded at a density of 300 000 cells per well (MDA-MB468), 100 000 per well (ARN8) or 50 000 per well (HNDF) in a volume of 2 mL of fully supplemented growth medium in a six-well plate (Fig 2A). Cells were seeded at a density of 85 000 cells per well (MDA-MB468) or 80 000 cells per well (ARN8) in a volume of 2 mL of fully supplemented growth medium in a six-well plate (Fig 2B and S3 Fig) Cells were incubated for 48 hours prior to dosing. Two hours prior to dosing, media was removed from the plate and replaced with fresh fully supplemented growth media. All compounds were diluted to 10× stock in fully supplemented growth media. Cells were incubated for six hours with the compounds before all media was removed and each well washed with 1× PBS twice and lysed in 150 μL of 1× LDS buffer (Invitrogen, Carlsbad, CA, USA #NP0008). Samples were heated to 95°C for five minutes and sonicated for 10 seconds three times before undergoing centrifugation briefly at 16 000 *g*. Protein levels of samples were ascertained using the bicinchoninic acid method [38] using a commercially available kit (Pierce, ThermoScientific #23227) with protein concentration normalised between samples. Samples were reduced using 100 mM DTT (Sigma-Aldrich #43819) and heated at 95°X for five minutes and loaded onto either 4–12% (Invitrogen #NP0322) or 12% (Invitrogen #NP0302) pre-cast *bis*-tris gels and run at 150 V in MOPS running buffer (Invitrogen #NP0001) with antioxidant present (Invitrogen #NP0005). Transfer was conducted using the NuPAGE transfer system (Invitrogen #NP00061) onto PVDF membranes (Millipore Billerica, MA, USA #IPVH00010) at 35 V for 90 minutes. All membranes were blocked in 5% milk (w/v) in PBS-T containing 0.1% tween 20 v/v (Sigma-Aldrich P9416). All antibodies were made up in 5% milk w/v in PBS-T. All antibody incubations were either overnight at 4°C or at room temperature for 1 hour. Primary antibodies for LC3B (Abcam, Cambridge, UK #Ab51520), p62 (Abcam #Ab109012), alpha tubulin (Abcam #Ab15246) or gapdh (Abcam #Ab8245) were diluted to concentrations of 1:3000, 1:5000 and 1:5000 v/v respectively. All secondary antibodies were horseradish peroxidase conjugated polyclonal rabbit anti-mouse (DAKO, Glostrup, Denmark #P0260) or swine anti-rabbit (DAKO #P0399) and diluted at concentrations of 1:1000 or 1:2000 v/v.

### LysoTracker cell staining

**Fig 3:**
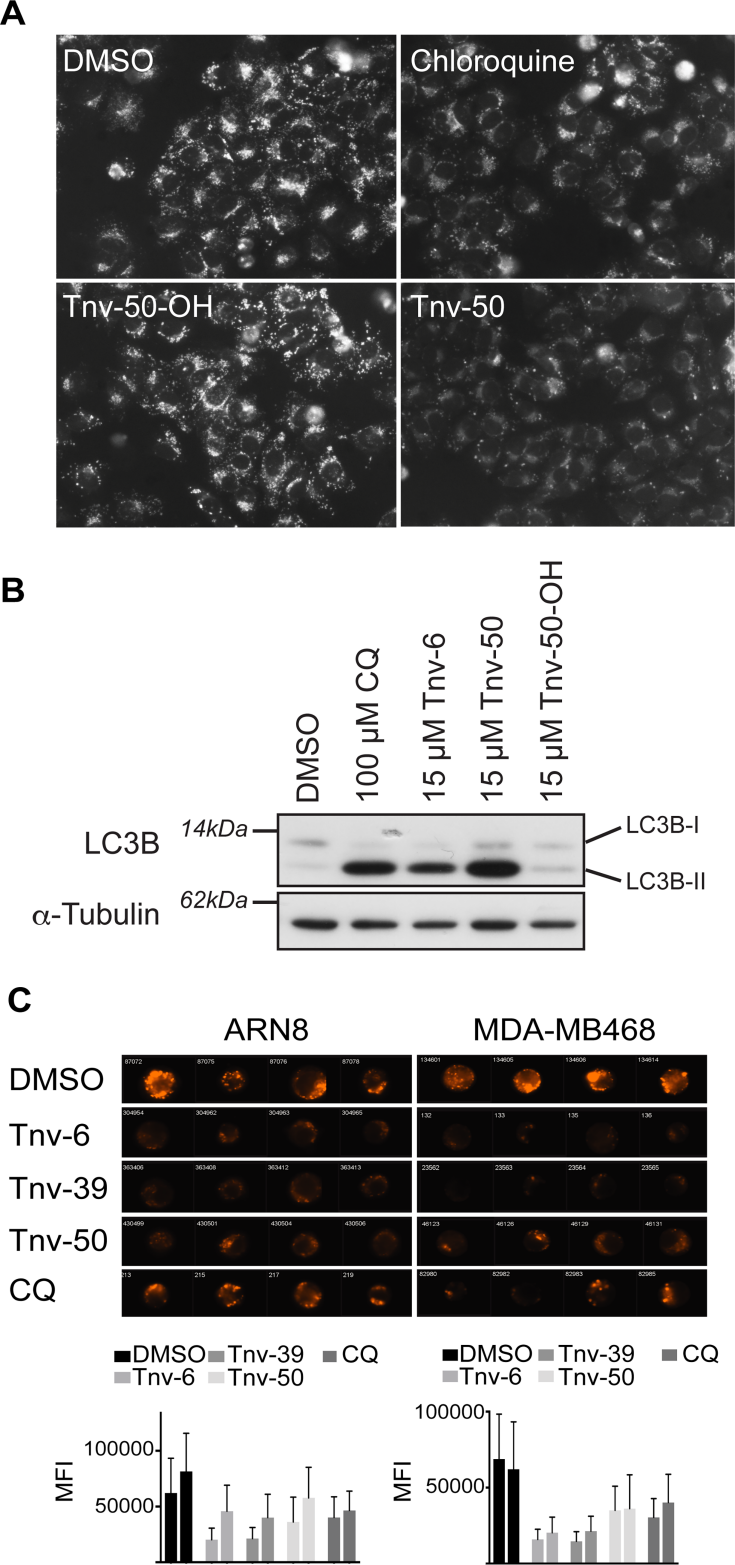
Tenovins prevent autophagic flux by a similar mechanism to chloroquine. (A) ARN8 cells stained with 500 nM of LysoTracker red after treatment with either 15 μM of tenovin-50-OH or tenovin-50, 100 μM chloroquine or vehicle for two hours prior to imaging at 32× magnification. (B) ARN8 cells treated with indicated compounds for six hours with LC3B and alpha-tubulin levels detected by western blot. (C) ARN8 and MDA-MB468 cells treated with 10 μM of tenovin-6, tenovin-39, tenovin-50 or 100 μM chloroquine (CQ) for six hours. Shown are representative images obtained using the ImageStream platform of LysoTracker stained cells and graphs of the mean fluorescence intensity from two independent biological repeats with each separate treatment group having >10 000 events in the singlet and focused gates counted for the analysis.

In Fig 3, ARN8 were seeded at 150 000 cells per well in fully supplemented media in a six-well plate and incubated as described above for 24 hours. Two hours prior to dosing, media was removed from the plate and replaced with serum free high glucose DMEM (HyClone #SH30243) containing 100 U mL^-1^ penicillin/streptomycin (Hyclone #SC30010). All compounds were made up to a 10× stock in serum free growth medium described above and added to cells for two hours. 500 nM lysotracker red (Invitrogen #L7528) was added to each well and the cells imaged after 5 minutes of incubation. Images were obtained on an Axiovert 40CFL microscope (Zeiss, Oberkochen, Germany #451212-0000-000) using an AxioCam MRc 5 (Zeiss #000000-0450-354) using Filter set 43 Cy 3 shift free (Zeiss #489043-0000-000) with excitation at 550 nm and observing emission at 605 nm. Plates were then returned to the incubator and imaged again two hours later.

### Clonogenic recovery assay

**Fig 4:**
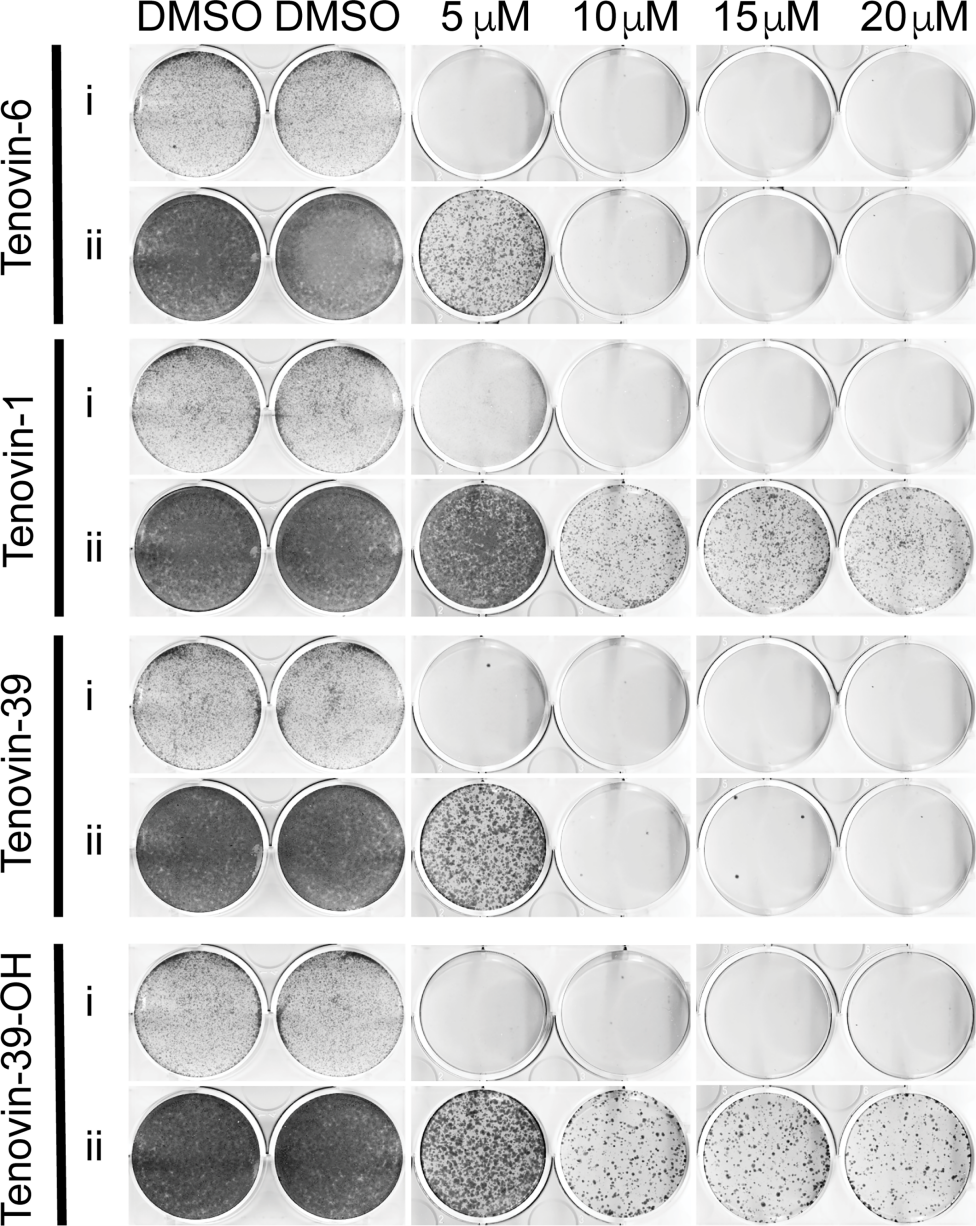
Tenovins eliminate melanoma cells in culture due to blockage of autophagic flux. Clonogenic assay in ARN8 human melanoma cells showing the ability of indicated tenovins to eliminate tumor cells in culture. (i) Cells were treated for 72 hours and stained with Giemsa to show pre-recovery cell density. (ii) Cells were treated for 72 hours with the indicated compounds. Following treatment, the medium was replaced and the cells allowed to grow for a set period of time as described in materials and methods followed by staining with Giemsa solution to show colonies of proliferating cells during recovery in compound-free medium.

For Fig 4 and S5 Fig, ARN8, A375, HT144 or SK-Mel-28 were seeded at 10 000 cells per well in six-well plates and incubated for 24 hours. Two hours prior to dosing, media was removed from the plate and replaced with fresh fully supplemented growth medium. All compounds were diluted to 10× stock in fully supplemented growth media. Cells were grown for 72 hours in the presence of the compounds. Following treatment, medium was removed and cells were washed twice with fully supplemented growth medium or a following treatment and then grown in fully supplemented medium for three to eight days prior to fixation unless otherwise stated. Following the recovery phase, growth media was removed and each well was washed twice with 1× PBS. Cells were fixed using 1:1 methanol:acetone v/v and incubated at–20°C for 10 minutes. The solvents were removed and cells left to air dry. Cells were stained with Giemsa stain (Sigma-Aldrich #48900) diluted to 7.5% v/v in 1× PBS. Cells were then washed with warm water to remove excess stain and allowed to dry.

### Flow cytometry

In S3 Fig panel A, HOS-EGFP-LC3 cells were grown and treated with tenovin-6 or tenovin-D3 at the stated doses for four hours. GFP-tagged LC3 levels were determined using previously established methods [37]. In Fig 3 and S3 Fig panel C, ARN8 and MDA-MB468 cells were grown in 75 cm^2^ flasks in fully supplemented growth medium until >90% confluency. Two hours prior to dosing medium was removed and replaced with high glucose DMEM (HyClone #SH30243) supplemented as indicated above. Cells were treated with indicated compounds for 6 hours prior to harvesting. 30 minutes before harvesting 50 nM LysoTracker red (Invitrogen #L7528) was added. Cell culture medium was removed and the cells washed with 1× PBS. Cells were trypsinised in 200 μL of 1× trypsin/EDTA (Sigma-Aldrich #T4174). Following detachment, cells were washed with fully supplemented growth medium and centrifuged at 200 *g* for 5 minutes. Cells were resuspended in 200 μL FACS Buffer (2 mM EDTA, 0.5% BSA in 1× PBS). Cells were run on an Imagestream X Mk II with excitation at 561 nm and emission in channel 4 (595–660 nm).

**Fig 5:**
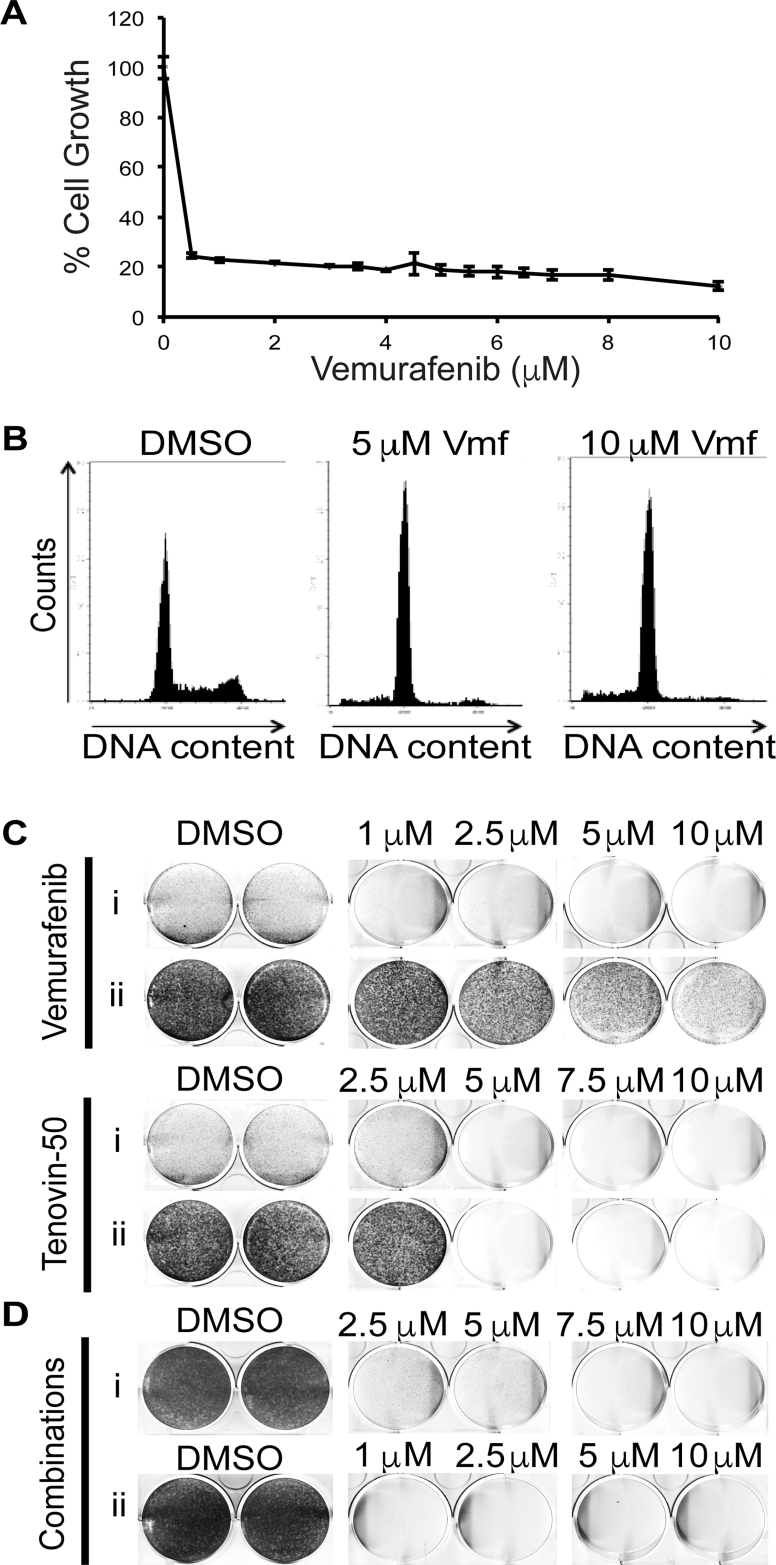
Autophagic flux blockage by tenovins leads to elimination of remaining melanoma cells following vemurafenib treatment. (A) SRB assay indicating surviving populations of ARN8 melanoma cells upon treatment with indicated doses of vemurafenib. (B) Flow cytometric analysis of ARN8 cells treated for 73 hours with indicated dose of vemurafenib and stained with propidium iodide. (C) Clonogenic assay in ARN8 melanoma cells treated as indicated for (i) 72 hours followed by staining with Giemsa to show surviving cells or (ii) 72 hours followed by four days of recovery in compound-free medium followed by staining with Giemsa stain to show cells capable of proliferating. (D) Clonogenic assay using ARN8 melanoma cells treated with (i) vemurafenib at 10 μM dose for 72 hours followed by a 72 hour treatment with vemurafenib at 10 μM and tenovin-50 at indicated doses or (ii) vemurafenib at indicated doses for 72 hours followed by 72 hours treatment with tenovin-50 at 7.5 μM. Both treatment groups were allowed four days of recovery in compound-free medium followed by staining with Giemsa solution to show cells capable of proliferating.

In Figs 1 and 5 and S1 Fig, ARN8 and HNDF cells were seeded at 50 000 or 30 000 per well respectively, in six-well plates and incubated for 24 hours. All compounds were diluted to 10× stocks in fully supplemented growth media. Cells were incubated with the compounds for 48 hours. Cell culture medium was removed and placed into tubes. Wells were washed twice with 1× PBS with the washes saved in the tubes to harvest floating dead cells. Cells remaining in the wells were trypsinised with 200 μL of 1× trypsin/EDTA (Sigma-Aldrich #T4174). Following detachment fresh growth media was added to each well and the contents removed and placed in the relevant tubes. Any remaining cells in the plate were then gathered by washing twice with 2 mL of 1× PBS with the washes saved and added to the relevant tubes. The tubes were centrifuged at 500 *g* for five minutes. Cell pellets were washed twice with 1× PBS. The pellets were resuspended in 1 mL of 1× PBS and added dropwise to 3 mL of 99.9% ethanol whilst vortex mixing at high speed. Cells were then fixed at –20°C for 24 hours. Following fixation, cells were pelleted at 1390 *g* for 5 minutes. The cells were washed three times with 2 mL of 1× PBS containing 3% FBS with centrifugation at 1390 *g* between each wash. Cells were resuspended in 0.5 mL PBS containing 30 μg propidium iodide (Invitrogen #P3566) and 75 μg RNAse (Sigma-Aldrich #R4642) and incubated on ice in the dark for 10 minutes. Flow cytometry was performed using a Becton Dickinson FACScan (Franklin Lakes, NJ, USA) with 10 000 events counted.

### Software

All microscopy was performed using AxioVision LE Module Fluorescence Lite (Zeiss #410130-0504-000). All western blots, microscopy and clonogenic plates were processed using Adobe Photoshop CS5.1 to adjust brightness, contrast, to crop, and to convert all images to greyscale. Figures were constructed using Microsoft PowerPoint:Mac 2011 (version 14.3.6) and Adobe Illustrator (version CS5.1). All statistical calculations and graphs were made using either Microsoft Excel:Mac 2011 version 14.3.6 or Graphpad Prism (version 7.0b). All pKa calculations were carried out using ADMET Predictor version 7.2 (Simulations Plus Inc). All propidium iodide flow cytometry was processed using BD CellQuest Pro software (version 5.1). All lysotracker flow cytometry obtained on the Imagestream X Mk II were analysed using the IDEAS software (version 6.2.183).

## Results

### High doses of tenovins achieve cell kill in melanoma cell lines independently of sirtuin inhibition and p53 activation

We have previously demonstrated that tenovin-6 is capable of inducing cell death in ARN8 melanoma cells both *in vitro* and *in vivo* [1]. Based on these prior discoveries, we compared eight different tenovin compounds to ascertain their ability to perturb cell growth in ARN8 cells and human normal dermal fibroblasts (HNDFs) as an untransformed control cell line (Fig 1). Following 72 hours of treatment, there were differences noted between the activities of tenovin-6, tenovin-D3 and tenovin-D1 as shown in panel A. Tenovin-6 is capable of inhibiting both SirT1 and SirT2, tenovin D3 inhibits SirT2 and tenovin-D1 is unable to inhibit either SirT1 or SirT2 [35]. The inability of tenovin-D1 to inhibit sirtuins does not fully ablate the effect of this compound on the proliferation of ARN8 cells. This result suggests that these tenovins may have another effect on cells, possibly relating to the common thiourea or the aliphatic polar side chain moieties they share with tenovin-6. Following SRB analysis, we conducted flow cytometry with propidium iodide staining to examine the effects on the cell cycle of a series of compound pairs at the high dose of 10 μM, namely tenovin-1, tenovin-6, tenovin-39, tenovin-39-OH, tenovin-50 and tenovin-50-OH, as well as chloroquine at 100 μM. As seen in Fig 1 panel B, tenovin-1, tenovin-39-OH and tenovin-50-OH led to cell cycle arrest in the HNDFs, whereas they showed a mixed sub-G1 and cell cycle arrest profile in the melanoma cells. By contrast, at these high doses, tenovin-6, tenovin-39 and tenovin-50 resulted in a significant amount of cell death in both the ARN8 and HNDF cultures. Chloroquine at high doses was also toxic to both cell lines. We also examined the effect of a 48 h dose titration on ARN8 and HNDF cells (S1 Fig panel A) and repeated the propidium iodide experiments and noted a very reproducible effect on cells (S1 Fig panel B).

We then carried out cellular thermal shift assays to determine whether tenovins studied were capable of stabilising SirT1 along a temperature gradient in cells. We confirmed that the previously studied tenovin-6 and 39 as well as novel tenovins 39-OH and 50 were able to stabilise SirT1 across a temperature gradient as well as in a concentration dependent manner confirming these compounds interact with SirT1 in cells (S1 Fig panel A–H) in a similar manner to the known SirT1 inhibitor, EX-527 (S1 Fig panels I and J) [39] without altering SirT1 levels (S1 Fig panel K). We also used the CCLE database to assess SirT1 levels in the cell lines used in this study and found that the expression of SirT1 was similar between immortalised fibroblasts, A375, MDA-MB468 and H1299 cells [40].

### Blockage of autophagic flux is dependent on the presence of an aliphatic tertiary amine

MAP1LC3, hereafter referred to as LC3, is essential for the formation of autophagosomal vesicles [41]. LC3 exists as several homologues in mammals, including LC3A, LC3B and LC3C and undergoes C-terminal cleavage to give the cytosolic form, LC3-I [41]. Under normal conditions, LC3 is not detected due to processing by Atg4 to LC3-I immediately following synthesis [42]. During the formation of autophagosomes, LC3-I undergoes conjugation to phosphatidylethanolamine to give what is often termed as its lipidated form LC3-II [18,41,43]. As LC3-II is associated with autophagosomes until the fusion with lysosomes, it is a very commonly used marker of the presence of autophagosomes in mammalian cells, with LC3B being the most commonly used homologue for autophagy assays [43,44]. Our collaborators have reported that tenovin-6 leads to the appearance of autophagic vacuoles in chronic lymphocytic leukaemia cells as well as markers of autophagy, such as LC3-II [31,32]. The findings motivated us to investigate whether all tenovin analogues possess the ability to perturb autophagy and whether this perturbation is cell-type specific.

p53 induces autophagy leading to a rise in LC3-I conversion to LC3-II [45]. This is thought to be through its ability to induce the expression of the autophagy modulator DRAM [46]. Therefore we decided to ascertain whether the effect of the tenovins on LC3-II levels is independent of wild-type p53. To this end we used cells that carry two different p53 mutations (MDA-MB468 and HOS-EGFP-LC3). Indeed, certain tenovins increased LC3B-II levels in cells lacking wild type p53 (Fig 2A and S2A Fig) suggesting that the effect of tenovins on autophagy is independent of p53-mediated transcription. Additionally, tenovin-D3, a tenovin that poorly inhibits SirT1 and does not activate p53 [35], also leads to an increase in LC3B-II levels (Fig 2A and S2A Fig). This observation further supports that the effect of tenovins on autophagy is independent of their ability to activate p53 or their effect on SirT1. This also serves to confirm other research that indicated a lack of relationship between SirT1 inhibition and autophagy with tenovins [33]. Other tenovins that fail to activate p53 and still increase LC3B-II levels include tenovin-D1, 30j, 30k and 50 (Table 1).

**Table 1:** Characterization of tenovin analogs and other studied compounds showing dependence of LC3B-II accumulation on the presence of the aliphatic tertiary amine.

Compound	Aliphatic Tertiary Amine	LC3B-II Accumulation^a^	p53 activation^b^	pKa^c^
**Tenovin-D1**	+	+	–	9.36
**Tenovin-D3**	+	+	–	9.32
**Tenovin-6**	+	+	+	9.45
**Tenovin-39**	+	+	+	9.49
**Tenovin-39-OH**	–	–	+	-0.13
**Tenovin-33**	+	+	+	9.43
**Tenovin-50**	+	+	–	9.15
**Tenovin-50-OH**	–	–	–	-0.5
**Tenovin 51**	–	–	+	0.23
**Tenovin-1**	–	–	+	-0.4
**Tenovin-3**	–	+	+	4.6
**Tenovin-30a**	+	+	+	9.45
**Tenovin-30b**	+	+	+	9.45
**Tenovin-30d**	+	+	+	9.46
**Tenovin-30j**	+	+	–	9.42
**Tenovin-30k**	+	+	–	9.43
**Tenovin-30n**	+	+	+	9.42
**5406085**	–	–	–	-1.59
**Chloroquine**	+	+	–	9.86

^a^LC3B-II accumulation assessed by western blotting in MDA-MB468 cells.

^b^Activation of p53 transcription factor activity was measured as described in (1) with + representing activity.

^c^pKa was calculated with respect to the site most likely to undergo protonation using software from ADMET Predictor version 7.2 (Simulations Plus Inc, 2015). All chemical structures are shown in either Fig 1 or S1 Table.

Tenovin-39 and tenovin-50 are able to induce high levels of LC3B-II levels (Fig 2A) whereas tenovin-39-OH and tenovin-50-OH cause no accumulation of LC3B-II. The main structural feature that differs in these pairs of compounds is the presence of the aliphatic tertiary amine in tenovin-39 and tenovin-50, or the terminal hydroxyl group in tenovin-39-OH and tenovin-50-OH. As shown in Fig 1A, the presence of the tertiary amine in tenovin-50 significantly contributes to cell growth inhibition. Similarly, tenovin-39 and tenovin-39-OH show the same pattern whereby tenovin-39-OH is unable to block autophagic flux (Fig 2A). From the above results and those summarised in Table 1, tenovins increase LC3B-II levels in a manner that is associated with the presence of the tertiary amine located at the end of the aliphatic chain. We do note, however, that the weakly basic aniline moiety of tenovin-3 is also able to partially block autophagy at high doses. We also noted the accumulation of p62 after 24 h of incubation with compounds (Fig 2B). This suggested very strongly that the effect of tenovins on autophagy was most likely to be due to blockage of autophagic flux rather than induction of autophagy.

The effect of this aliphatic tertiary amine in the tenovins on LC3B may be due to either autophagic blockage or increased formation of autophagosomes as a rise in LC3B-II can occur in both scenarios [43]. To distinguish between these two possibilities we co-incubated cells with saturating concentrations of chloroquine (CQ) to ensure maximal autophagic flux blockage, and a single dose of tenovin-39, tenovin-39-OH, tenovin-50 and tenovin-50-OH to see whether there was any unobservable change in autophagy induction or blockage. As shown in Fig 2C, neither tenovin-39 nor tenovin-50 lead to a significant increase in LC3B-II levels in the presence of CQ, whilst tenovin-39-OH and tenovin-50-OH also were unable to ablate CQ blockage of autophagy. This suggests that the tertiary amine is causing a block in autophagic flux rather than induction of autophagosome formation as there was no additive effect in combination on the levels of LC3B-II [43,47]. To test whether these effects were tumour cell specific, we also tested tenovins 50, 50-OH, 39 and 39-OH against CQ as a positive control in HNDF cells (S3 Fig). We observed the same pattern of autophagic flux blockage with these tenovins in the untransformed cells, confirming that the blockage of autophagic flux can occur in non-tumour cells. We also examined the ARN8 melanoma cells that possess wild-type p53 to see whether there was any effect on autophagy in those cells in the presence of wild-type p53 (S4 Fig). We noted a high degree of correlation between the ARN8 and MDA-MB468 cells, with a notable accumulation of p62 in these cells along with increases in LC3B, suggesting that the same mechanism for the increase in LC3B is true in both p53 wild-type and mutant cell lines.

Reinforcing these results we carried out experiments using the weakly basic lysosomotropic dye, LysoTracker, that fluoresces under acidic conditions, and assessed the appearance of cytoplasmic puncta (Fig 3A). These assays demonstrated the propensity of tenovin-50, in a similar manner to the lysosomotropic agent CQ, to reduce the staining of lysosomes by LysoTracker indicating alkalinisation of the lysosomes due to weakly basic moieties in their structures. In contrast, tenovin-50-OH, a tenovin-50 analogue lacking the tertiary amine, was unable to alter LysoTracker staining of the lysosomes (Fig 3A) as well as being unable to increase LC3B-II levels (Fig 2A). We further characterised the ability of other tenovins with aliphatic tertiary amines to reduce LysoTracker signal in both ARN8 and MDA-MB468 cells. We found that the trend to reduce LysoTracker signal was unique for tenovins in possession of a tertiary amine (Fig 3C and S3 Fig). This suggests that the presence of the weakly basic tertiary amine is necessary for alkalinisation of the lysosomes and for autophagic blockage.

### Tenovins capable of blocking autophagic flux achieve total eradication of melanoma cells in culture

Based on the growth inhibitory curves in Fig 1, it appears that tenovin-1 and tenovin 39-OH, despite being able to activate p53, fail to achieve total cell kill based on the small residual population of cells following treatment. To test whether these residual cells are able to resume proliferation upon removal of the compounds, we conducted clonogenic assays using a panel of tenovin-1, tenovin-6, tenovin-39 and tenovin-39-OH as these compounds showed strong activity in the SRB assay (Fig 1). As shown in Fig 4, tenovin-1 and tenovin-39-OH treatment resulted in a residual viable population of ARN8 tumour cells that could proliferate upon removal of the compounds. In contrast the few remaining cells following tenovin-6 and tenovin-39 treatment did not proliferate or did so to a much lower extent, an effect that was masked in the SRB assay. This demonstrated that the two tenovins that were unable to block autophagic flux were also unable to achieve total cell kill.

To see whether this observation held for other autophagy blockers in our series we used tenovin-50 as a molecular tool to test this hypothesis. As mentioned earlier tenovin-50 is a potent inhibitor of autophagic flux. For this reason, tenovin-50 was tested in a panel of three melanoma lines; the ARN8 parental cell line A375 as well as SK-Mel-28 and HT144 cells (S5 Fig). Based on this analysis, tenovin-50 achieved total eradication in all melanoma cell lines tested, including the SK-Mel-28 cells that possess mutated p53 [48]. This further cemented the observation that autophagy is the pivotal factor for achieving total melanoma cell kill in clonogenic recovery assays with the tenovins in this cell line.

### Tenovins capable of blocking autophagic flux cooperate with vemurafenib and achieve total kill in melanoma cell lines

The human melanoma cell line A375, the parental cell line for ARN8 cells [36], expresses activated kinase B-Raf^V600E^ [49]. Vemurafenib (PLX4032), a potent and selective inhibitor of B-Raf^V600E^, is currently in the clinic for advanced stage melanoma [11]. We observed that in a SRB growth inhibitory assay with vemurafenib in ARN8 cells that there is a significant portion of cells remaining after treatment even at concentrations far beyond the reference GI_50_ of 47 nM in A375 cells (Fig 5A and 5B) [50]. Following this observation, we tested whether any residual vemurafenib-insensitive population could resume proliferation in other melanoma cell lines possessing the B-Raf^V600E^ mutation [11]. As shown in S5 Fig, A375, HT144 and SK-Mel-28, cells that have been previously reported to be sensitive to B-Raf inhibition, when treated with vemurafenib could proliferate upon removal of the drug [11]. We also examined the cell cycle using propidium iodide staining of DNA following vemurafenib treatment (Fig 5B). This analysis revealed that vemurafenib predominantly led to cell cycle arrest in G1 of the cell cycle after 48 hours of treatment, as well as a small population of cells in sub-G1. To test whether a tenovin capable of inhibiting autophagy could eliminate cells surviving vemurafenib treatment, we treated cells with tenovin-50 after 72 hours of vemurafenib treatment and also in combination with vemurafenib at various doses (Fig 5C and 5D). Most interestingly, we found that tenovin-50 was able eliminate cells upon co-treatment with vemurafenib. Additionally, it was able to ablate growth and achieve total kill of cells arrested in G1 by pre-treatment with vemurafenib (Fig 5D).

## Discussion

The current targeted clinical treatment of melanoma consists of two key therapeutic areas, vemurafenib against BRAF mutant cells, and anti-immune checkpoint therapies, both of which display very visible clinical benefits [13,15,16]. It is, however, highly unfortunate that when combined these therapies demonstrate clear dose-limiting toxicities, rendering synergy impossible in a clinical setting [15,16]. It is, therefore, of importance to explore the possibility of using another non-genotoxic therapy in combination with one of these current, clinically approved compounds to improve survival of melanoma patients. With the interest in inhibitors of autophagy in combination with current chemotherapeutics as proposed for CML in combination with imatinib, combination of blockers of autophagic flux with vemurafenib or other protein kinase inhibitors might be of interest, if total tumour cell kill can be demonstrated *in vitro* [21].

It has been previously reported that tenovin-6 leads to the appearance of autophagic vacuoles in chronic lymphocytic leukaemia cells [31,32]. In the present study we have expanded on this observation by elucidating the mechanism by which tenovins lead to the accumulation of the autophagic vacuoles, as well as demonstrating the pivotal nature of the aliphatic tertiary amine in mediating the appearance of autophagic vacuoles. Coupled with these discoveries, we also demonstrate that autophagic blockage by the tenovins leads to complete tumour cell kill and elimination of melanoma cells that survive B-Raf inhibition with vemurafenib.

During the target elucidation process of the tenovins, the aliphatic chain was incorporated to increase the water solubility of tenovin-1, the initial hit compound from a screen for activators of the p53-response in cells [1]. Through modelling the pKa of each molecule (Table 1), this tertiary amine, as part of the aliphatic chain, is predicted to have a pKa between 9.15 and 9.49. Compounds with aliphatic tertiary amines, including CQ, which has a pKa of 9.86, have been demonstrated to act as weak bases that become protonated in acidic organelles, such as lysosomes or the intermembrane space of the mitochondria [51,52]. Protonation of a compound reduces its membrane permeability and therefore can result in its accumulation in acidic organelles [51]. The accumulation of small molecules in lysosomes leads to the alkalinisation of these compartments and subsequent inhibition of lysosomal hydrolases [19,24,51–53]. The lysosomal accumulation of CQ has previously been shown to be responsible for blockage of autophagic flux through inhibition of lysosomal hydrolases and the lack of fusion of lysosomes with autophagosomes [28,29]. Our observations using a panel of tenovin analogues suggest that tenovins containing the aliphatic tertiary amine lead to a rise in LC3B-II levels by a similar mechanism to CQ. The tenovins with a tertiary amine, particularly tenovin-50, reach a dose capable of maximally blocking autophagy at a lower micromolar concentration than CQ. One exception to this rule is tenovin-3 which also leads to a very slight rise in LC3B-II despite lacking an aliphatic tertiary amine. This tenovin, however, possesses an aniline that may also undergo protonation and sequestration in acidic organelles, thus increasing expression of LC3B-II, albeit to a lesser degree than seen with the tenovins with an aliphatic tertiary amine (see S1 Table for all structures). This strong structure-activity relationship between the presence of the tertiary amine and blockage of autophagic flux suggests the requirement for a basic moiety to achieve this blockage.

We have already demonstrated that tenovin-6 elicits a cell cycle arrest in HNDFs at concentrations less than 5 μM [54]. However, at concentrations above 5 μM, toxicity to the fibroblasts starts to increase. Here we confirm these results in both SRB assays and flow cytometry analysis where tenovin-6 results in death at higher concentrations. This is in contrast to tenovin-1, which demonstrates a cytostatic phenotype in HNDFs whilst causing death in ARN8 cells. Indeed, this observation is recapitulated when we examine the difference between tenovin-39 and tenovin-39-OH, where tenovin-39 results in cell death after 48 hours of exposure whilst tenovin-39-OH results purely in cell-cycle arrest after 48 hours in HNDFs. This suggests the tertiary amine in the tenovins may lead to undesired toxicity of these molecules at concentrations above 5 μM. We have also seen that the point at which autophagic blockage occurs coincides at approximately 5 μM of compound. This evidence supports the conclusion that autophagic blockage by the compounds possessing a tertiary amine results in toxicity to both normal and tumour cells.

Whilst these results insinuate that autophagic blockage may be undesirable when one considers mitigation of unwanted toxicity, we have also determined that autophagic blockage is required for complete tumour kill. Our clonogenic assays establish that a reservoir of tumour cells resistant to treatment with tenovin-1 and tenovin-39-OH had the ability to survive and continue to proliferate. This was in stark contrast to tumour cells treated with tenovin-6 or tenovin-39 where it was evident that elimination of all tumour cells was achieved at concentrations where autophagic blockage was induced. This suggests that for complete kill to be achieved, autophagic blockage is absolutely essential.

To complete the picture of the importance of autophagic blockage in the treatment of tumours, we compared the results using our most potent autophagy inhibitor, tenovin-50, with a current treatment against melanoma, the B-Raf inhibitor vemurafenib. At present there is no clear evidence to suggest that vemurafenib is a curative drug [14]. Our clonogenic assay (Fig 5) with melanoma cells possessing the B-Raf^V600E^ mutation demonstrates that vemurafenib, in spite of its potency, is unable to kill all cells and indeed primarily leads to arrest in the G1 phase of the cell cycle. This is in contrast to our observations with tenovin-50 treatment where total kill was achieved. Coupled with this result, we have also seen that tenovin-50 is capable of killing cells arrested in G1 following vemurafenib treatment even whilst co-administered with vemurafenib.

Altogether, these results illustrate that the aliphatic tertiary amine of the tenovins is essential for the blockage of autophagic flux. Taken with our other results, we conclude that the blockage of autophagic flux contributes directly to achieving complete kill in cultures of tumour cells by tenovins, but concurrently increases toxicity in normal cells. Additionally, we also show that tenovin-50 is capable of eliminating melanoma cells that survive vemurafenib treatment. The information provided by these studies may lead to strategies to increase relapse free survival in cancer patients.

## Supporting information

S1 Fig Tenovins are capable of affecting viability at 48 hours and repeat the pattern seen in Fig 1. (A) SRB analysis following treatment with various tenovins for 48 hours. (B) FACS analysis of propidium iodide staining following treatment with 10 μM of tenovin-1, tenovin-6, tenovin-39, tenovin-39-OH, tenovin-50 and tenovin-50-OH or 100 μm chloroquine (CQ) for 48 hours.
(TIF)

S2 Fig Certain tenovins demonstrate target engagement with SirT1 despite being unable to alter SirT1 levels. Western blot analysis of two different types of CETSA using H1299 cells (A-H). (A,C,E and G) Temperature gradient showing stabilisation of SIRT1 by tenovins 6, 39, 39-OH and 50 at fixed doses (20 μM) as compared to vehicle (DMSO). (B,D,F and H) Dose titration of tenovins 6, 39, 39-OH and 50 showing the dose dependency of the thermal stabilisation of SIRT1. Blots are quantified following normalisation to total protein loading in each lane and graphed below. (K) Western blot using H1299 cells showing SirT1 levels upon treatment. For all experiments the treatments with tenovins 6, 39, 50 or EX 527 were for two hours and for tenovin-39-OH for four hours.
(TIF)

S3 Fig Differential effect of tenovins on autophagy in various cell lines. (A) HOS cells expressing a GFP-LC3 plasmid showing the increase in lipidated LC3 levels upon treatment with tenovin-6 or tenovin-D3 for four hours as measured by flow cytometry. (B) HNDF cells were treated with 15 μM tenovin-50, tenovin-50-OH, tenovin 39, tenovin-39-OH or 100 μM chloroquine for six hours followed by detection of LC3B and alpha-tubulin by western blot. (C) ARN8 or MDA-MB468 cells were treated with the indicated compounds or vehicle control (DMSO) at 10 μM concentration for six hours prior to staining with LysoTracker red and analysed using the ImageStream X Mk II. Median fluorescence intensity of LysoTracker was calculated for each treatment and plotted below.
(TIF)

S4 Fig ARN8 cells demonstrate a similar pattern of autophagy blockage as MDA-MB468 cells. (A) Western blot analysis of ARN8 cells treated with 10 μM of the indicated compounds for the indicated times. (B) Western blot analysis of ARN8 cells treated for 6 h with the indicated compounds.
(TIF)

S5 Fig Effect of tenovins in combination with vemurafenib on various melanomas possessing the B-Raf^V600E^ mutation. Clonogenic assay in A375 (A), HT144 (B) or SK-Mel28 (C) human melanoma cells showing the ability of various tenovins to eliminate tumor cells in culture. (i) Cells were treated for 72 hours and stained with giemsa stain to show pre-recovery cell number. (ii) Cells were treated for 72 hours with the medium replaced and the cells allowed to grow for a set period of time as described in materials and methods followed by staining with giemsa stain to show surviving cells that proliferate during recovery from treatment.
(TIF)

S1 Table Structures and nomeclature of all tenovins used in this paper. (DOCX)

S1 File Supplemental materials and methods. (DOCX)

S2 File Chemical synthetic route for all tenovins not previously published. (DOCX)

S3 File Full blot images for all western blots in this study. (PDF)
