# Supplementary figures and images for: Autophagic flux blockage by accumulation of weakly basic tenovins leads to elimination of B-Raf mutant tumour cells that survive vemurafenib
Source: PLoS One. 2018 Apr 23;13(4):e0195956. doi: 10.1371/journal.pone.0195956 (PMC5912769; doi:10.1371/journal.pone.0195956)

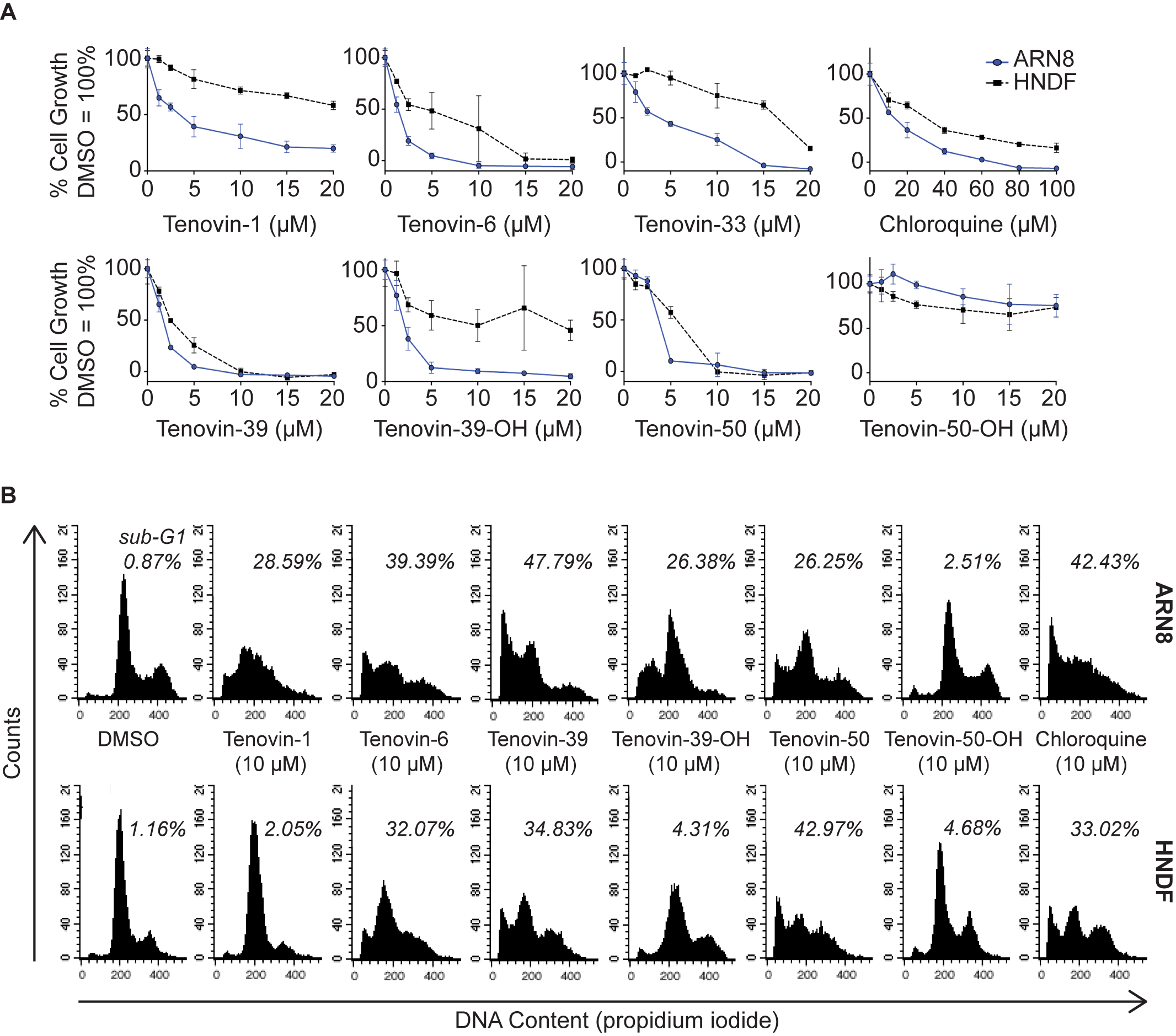

Supplement: S1 Fig — (A) SRB analysis following treatment with various tenovins for 48 hours. (B) FACS analysis of propidium iodide staining following treatment with 10 μM of tenovin-1, tenovin-6, tenovin-39, tenovin-39-OH, tenovin-50 and tenovin-50-OH or 100 μm chloroquine (CQ) for 48 hours. (TIF) [file pone.0195956.s001.tif]

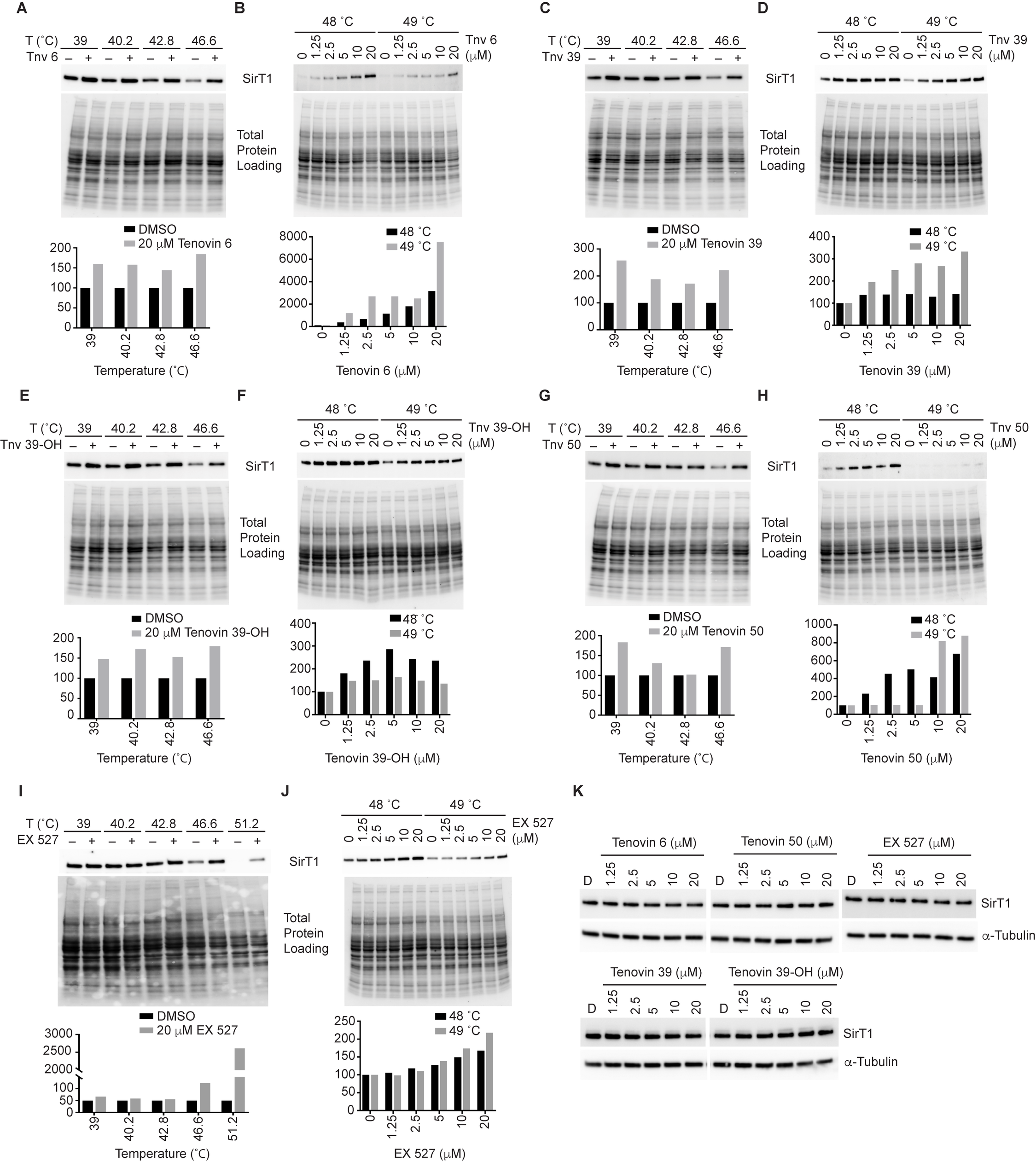

Supplement: S2 Fig — Western blot analysis of two different types of CETSA using H1299 cells (A-H). (A,C,E and G) Temperature gradient showing stabilisation of SIRT1 by tenovins 6, 39, 39-OH and 50 at fixed doses (20 μM) as compared to vehicle (DMSO). (B,D,F and H) Dose titration of tenovins 6, 39, 39-OH and 50 showing the dose dependency of the thermal stabilisation of SIRT1. Blots are quantified following normalisation to total protein loading in each lane and graphed below. (K) Western blot using H1299 cells showing SirT1 levels upon treatment. For all experiments the treatments with tenovins 6, 39, 50 or EX 527 were for two hours and for tenovin-39-OH for four hours. (TIF) [file pone.0195956.s002.tif]

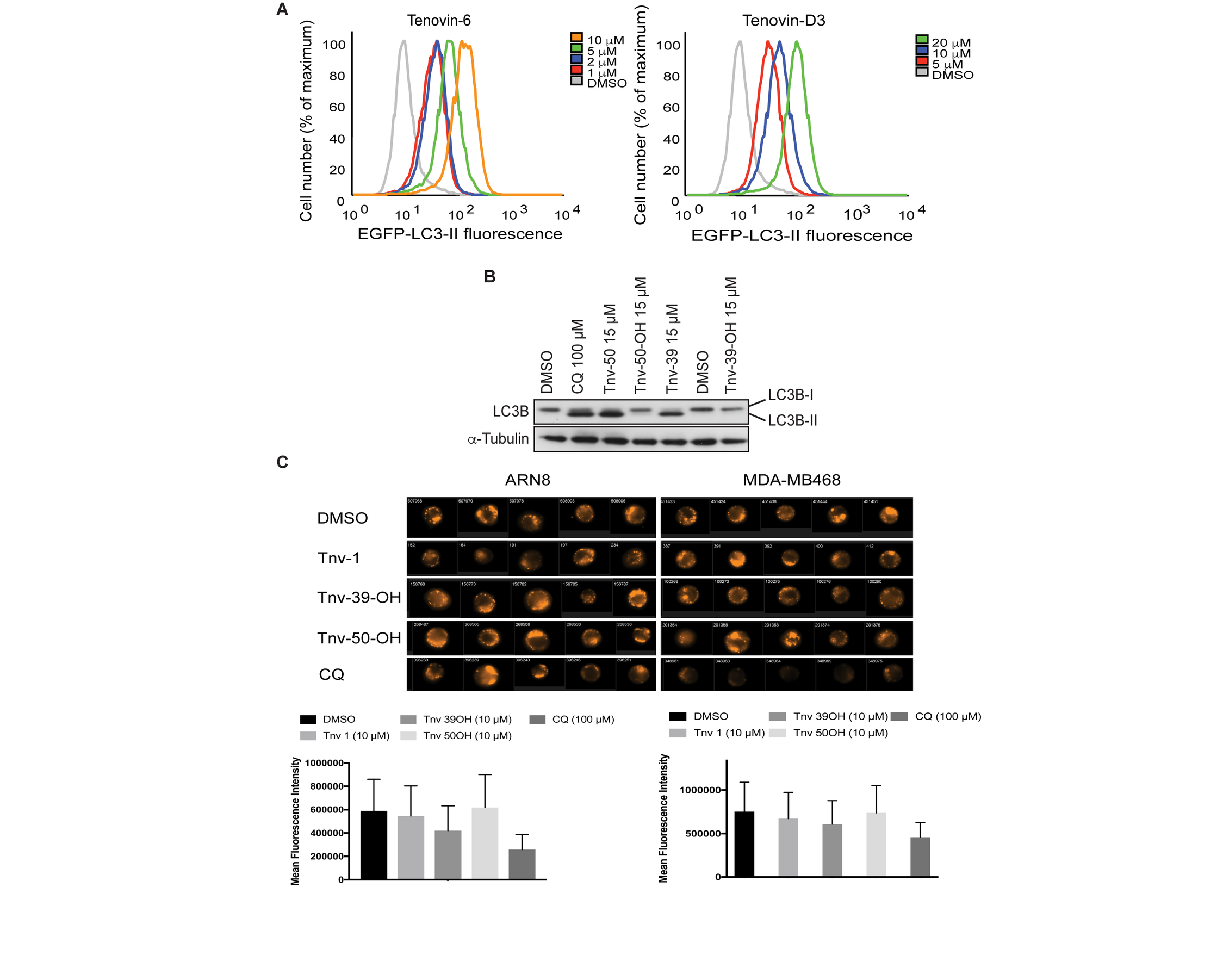

Supplement: S3 Fig — (A) HOS cells expressing a GFP-LC3 plasmid showing the increase in lipidated LC3 levels upon treatment with tenovin-6 or tenovin-D3 for four hours as measured by flow cytometry. (B) HNDF cells were treated with 15 μM tenovin-50, tenovin-50-OH, tenovin 39, tenovin-39-OH or 100 μM chloroquine for six hours followed by detection of LC3B and alpha-tubulin by western blot. (C) ARN8 or MDA-MB468 cells were treated with the indicated compounds or vehicle control (DMSO) at 10 μM concentration for six hours prior to staining with LysoTracker red and analysed using the ImageStream X Mk II. Median fluorescence intensity of LysoTracker was calculated for each treatment and plotted below. (TIF) [file pone.0195956.s003.tif]

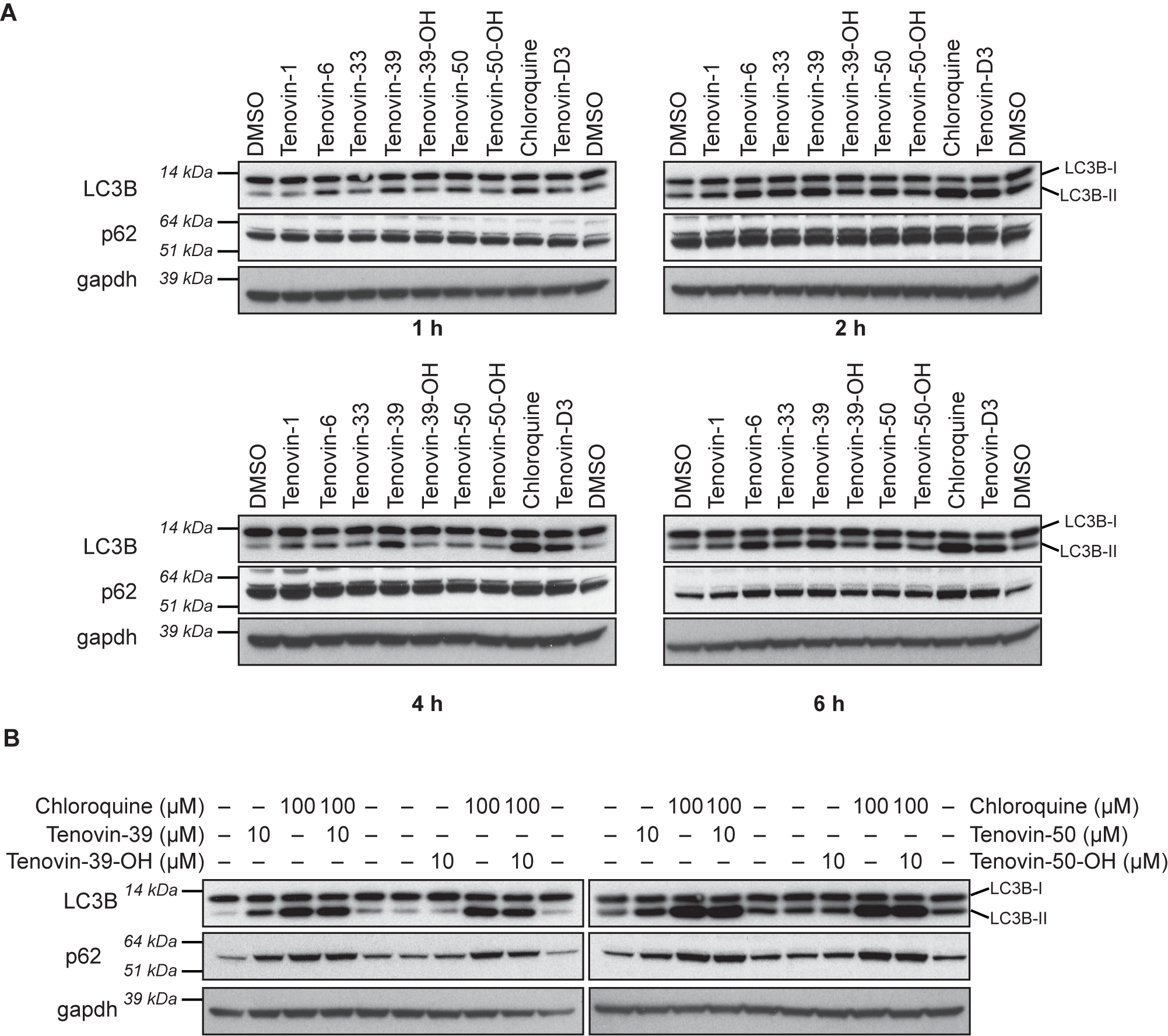

Supplement: S4 Fig — (A) Western blot analysis of ARN8 cells treated with 10 μM of the indicated compounds for the indicated times. (B) Western blot analysis of ARN8 cells treated for 6 h with the indicated compounds. (TIF) [file pone.0195956.s004.tif]

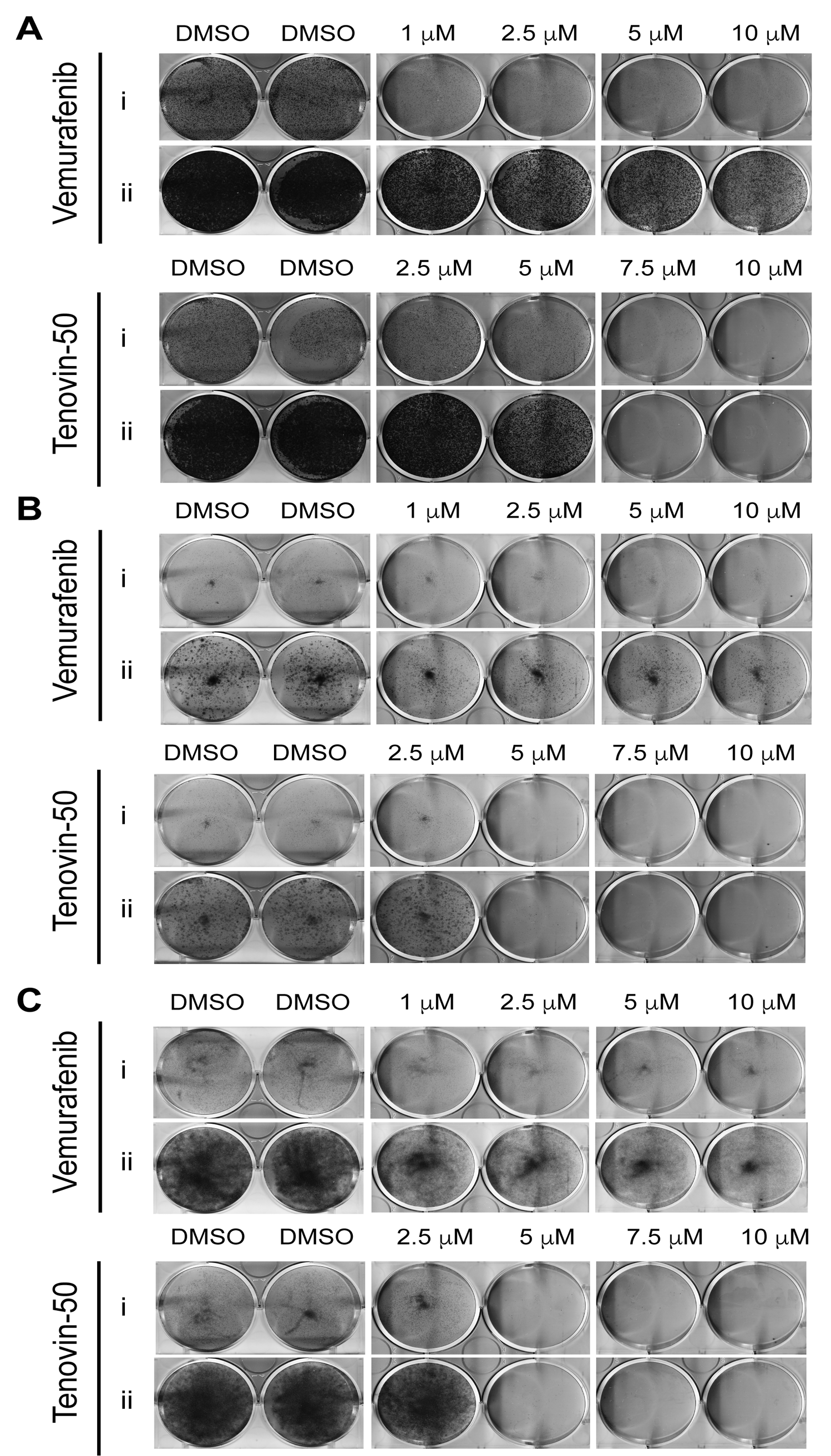

Supplement: S5 Fig — Clonogenic assay in A375 (A), HT144 (B) or SK-Mel28 (C) human melanoma cells showing the ability of various tenovins to eliminate tumor cells in culture. (i) Cells were treated for 72 hours and stained with giemsa stain to show pre-recovery cell number. (ii) Cells were treated for 72 hours with the medium replaced and the cells allowed to grow for a set period of time as described in materials and methods followed by staining with giemsa stain to show surviving cells that proliferate during recovery from treatment. (TIF) [file pone.0195956.s005.tif]
