# Supplementary material for: Autophagic flux blockage by accumulation of weakly basic tenovins leads to elimination of B-Raf mutant tumour cells that survive vemurafenib
Source: PLoS One. 2018 Apr 23;13(4):e0195956. doi: 10.1371/journal.pone.0195956 (PMC5912769; doi:10.1371/journal.pone.0195956)
Supplement: S1 Table — (DOCX) [file pone.0195956.s006.docx]

| **Table S1.** Structures and nomenclature of tenovin compounds referenced in the paper. | |
| --- | --- |
| **Structure** | **Name** |
|  |  |
|  | Tenovin-51 |
|  | Tenovin-30a |
|  | Tenovin-30b |
|  | Tenovin-30d |
|  | Tenovin-30j |
|  | Tenovin-30k |
|  | Tenovin-30n |
|  | Tenovin-3 |
|  | 5406085 |
