# Supplementary material for: Autophagic flux blockage by accumulation of weakly basic tenovins leads to elimination of B-Raf mutant tumour cells that survive vemurafenib
Source: PLoS One. 2018 Apr 23;13(4):e0195956. doi: 10.1371/journal.pone.0195956 (PMC5912769; doi:10.1371/journal.pone.0195956)
Supplement: S1 File — (DOCX) [file pone.0195956.s007.docx]

of CETSA (A-B**S1 File. Supplemental materials and methods.**

Marcus J G W Ladds^1*^, Andrés Pastor-Fernández^1^, Gergana Popova^1^, Ingeborg M M van Leeuwen^1^, Kai Er Eng^1^, Catherine J Drummond^1^, Lars Johansson^2^, Richard Svensson^3^, Nicholas J Westwood^4^, Anna R McCarthy^1^, Fredrik Tholander^5^, Mihaela Popa^6^, David P Lane^1^, Emmet McCormack^6,7^, Gerald M McInerney^1^, Ravi Bhatia^8^ and Sonia Laín^1*^

^1^ Department of Microbiology, Tumor and Cell Biology, Karolinska Institutet, Stockholm SE 171-77, Sweden

^2^ Chemical Biology Consortium Sweden, Science for Life Laboratory, Division of Translational Medicine and Chemical Biology, Department of Medical Biochemistry and Biophysics, Karolinska Institutet, SE 171-77, Sweden

^3^ Department of Pharmacy, Uppsala University Drug Optimization and Pharmaceutical Profiling Platform (UDOPP), Department of Pharmacy, Uppsala University, SE-752 37, Sweden

^4^ School of Chemistry and Biomedical Science Research Complex, University of St Andrews and EaStCHEM, St Andrews, Fife, KY16 9ST, Scotland, UK

^5^ Department of Medical Biochemistry and Biophysics, Karolinska Institutet, Stockholm SE 171-77, Sweden

^6^ Department of Clinical Science, University of Bergen, Jonas Liesvei 91B, Bergen N-5020, Norway

^7^ Department of Internal Medicine, Hematology Section, Haukeland University Hospital, Jonaes Leisvei 65, Bergen, N-5021, Norway

^8^ Department of Hematology and Oncology, University of Alabama, 1720 2nd Avenue South, NP2540, Birmingham, Alabama, 35294-3300, United States of America

**Corresponding Author**

*E-mail: marcus.ladds@ki.se Telephone: +46 8 524 846 03

sonia.lain@ki.se Telephone: +46 8 524 846 03

**Supplemental Methods**

**CETSA and Stain-free Western Blot**

Cells were seeded and allowed to grow to confluence in either a 75 cm^2^ flask (for temperature gradient experiments) or 25 cm^2^ flasks (for concentration gradients) with one flask per dose condition in fully supplemented RPMI as described in main methods. All medium was removed and cells were then treated for either 2 hours (tenovin-6, 39, 50 and EX 527) or 4 hours (tenovin-39-OH) made up in fresh medium. Following incubation, media was removed and the cells washed with 1× PBS and detached by trypsinisation (Sigma-Aldrich #T4174). Cells were then centrifuged at 200 *g* for 5 minutes and the trypsin removed. The cells were resuspended in 460 μL of 1× PBS for the temperature gradient or 160 μL of 1× PBS for the concentration gradient. Cells were then divided into labelled PCR tubes in a volume of 60 μL per tube. For the temperature gradient experiment, the PCR tubes were loaded into a thermocycler set to a temperature gradient with six different temperatures (39˚C, 40.2˚C, 42.8˚C, 46.6˚C, 51.2˚C and 55.1˚C) for six minutes followed by cooling to 22˚C for two minutes. For the concentration gradient, the PCR tubes were incubated at either 48˚C or 49˚C for six minutes followed by cooling to 22˚C for two minutes. The tubes for either experiment were then snap frozen in liquid nitrogen for two minutes and then allowed to thaw totally before repeating the snap freezing twice more. The contents of the PCR tubes were transferred to individual microfuge tubes (1.5 mL) and centrifuged at 16 000*g* for 45 minutes. The supernatant was transferred to a clean microfuge tube (1.5 mL) and an appropriate measure of 4× BioRad Laemmli loading buffer (BioRad #1610747) to give a final concentration of 1× as well as DTT (Sigma-Aldrich #43819) to a final concentration of 100 μM was added to each sample. The samples were run on 12-well stain free TGX gels (BioRad #4568085) in standard tris-glycine running buffer (BioRad #1610732) at 150 V. Prior to transfer, the gels were activated using the ChemiDoc Touch (BioRad #1708370) stain free gel activation protocol for five minutes. The gel was then blotted using the Trans-Blot Turbo transfer system (Biorad #1704150) using the PVDF membrane Trans-Blot Turbo kit (BioRad #1704272) for sandwich assembly. The transfer was conducted using the Trans-Blot Turbo pre-set programme for standard semidry transfer (30 minutes). All membranes were blocked in 5% milk (w/v) in PBS-T containing 0.1% tween 20 v/v (Sigma-Aldrich P9416). All antibodies were made up in 5% milk w/v in PBS-T. All antibody incubations were either overnight at 4°C or at room temperature for 1 hour. Primary antibodies for SirT1 were either mouse monoclonal (Millipore #05-1243) or rabbit monoclonal (Abcam #32441) diluted to 1:1000 v/v or 1:5000 v/v respectively. All secondary antibodies were horseradish peroxidase conjugated polyclonal rabbit anti-mouse (DAKO, Glostrup, Denmark #P0260) or swine anti-rabbit (DAKO #P0399) and diluted at concentrations of 1:1000 v/v. Imaging of the blots used the ChemiDoc Touch system for both the chemiluminescence mode for SirT1 with development using the Clarity substrate (BioRad #1705060) or stain free membrane mode for the total protein loading. All blots were normalised to their respective vehicle control and quantified using ImageLab (BioRad version 5.2.1 build 11) and graphed using Graphpad Prism (version 7.0b).
