# Supplementary material for: Autophagic flux blockage by accumulation of weakly basic tenovins leads to elimination of B-Raf mutant tumour cells that survive vemurafenib
Source: PLoS One. 2018 Apr 23;13(4):e0195956. doi: 10.1371/journal.pone.0195956 (PMC5912769; doi:10.1371/journal.pone.0195956)
Supplement: S2 File — (DOCX) [file pone.0195956.s008.docx]

of CETSA (A-B**S2 File. Chemical synthetic route for all tenovins not previously published**

Marcus J G W Ladds^1*^, Andrés Pastor-Fernández^1^, Gergana Popova^1^, Ingeborg M M van Leeuwen^1^, Kai Er Eng^1^, Catherine J Drummond^1^, Lars Johansson^2^, Richard Svensson^3^, Nicholas J Westwood^4^, Anna R McCarthy^1^, Fredrik Tholander^5^, Mihaela Popa^6^, David P Lane^1^, Emmet McCormack^6,7^, Gerald M McInerney^1^, Ravi Bhatia^8^ and Sonia Laín^1*^

^1^ Department of Microbiology, Tumor and Cell Biology, Karolinska Institutet, Stockholm SE 171-77, Sweden

^2^ Chemical Biology Consortium Sweden, Science for Life Laboratory, Division of Translational Medicine and Chemical Biology, Department of Medical Biochemistry and Biophysics, Karolinska Institutet, SE 171-77, Sweden

^3^ Department of Pharmacy, Uppsala University Drug Optimization and Pharmaceutical Profiling Platform (UDOPP), Department of Pharmacy, Uppsala University, SE-752 37, Sweden

^4^ School of Chemistry and Biomedical Science Research Complex, University of St Andrews and EaStCHEM, St Andrews, Fife, KY16 9ST, Scotland, UK

^5^ Department of Medical Biochemistry and Biophysics, Karolinska Institutet, Stockholm SE 171-77, Sweden

^6^ Department of Clinical Science, University of Bergen, Jonas Liesvei 91B, Bergen N-5020, Norway

^7^ Department of Internal Medicine, Hematology Section, Haukeland University Hospital, Jonaes Leisvei 65, Bergen, N-5021, Norway

^8^ Department of Hematology and Oncology, University of Alabama, 1720 2nd Avenue South, NP2540, Birmingham, Alabama, 35294-3300, United States of America

**Corresponding Author**

*E-mail: marcus.ladds@ki.se Telephone: +46 8 524 846 03

sonia.lain@ki.se Telephone: +46 8 524 846 03

**Synthesis and characterization of tenovin-50, tenovin-50-OH, tenovin-39-OH and tenovin-51**

**Materials and Methods**

Starting materials and reagents were obtained from commercial suppliers and used without further purification. ^1^H NMR spectra were measured at room temperature (298 K) on a Bruker DPX 400 (^1^H NMR = 400 MHz) instrument. Deuterated solvents were used and ^1^H NMR chemical shifts internally referenced to CHCl_3_ (7.26 ppm) in chloroform-*d*_1_ solution, and to CD_2_HSO_2_CD_3_ (2.6 ppm) in dimethylsulfoxide-*d*_6_. Chemical shifts are expressed as δ in units of ppm. ^13^C NMR spectra were recorded under the same conditions and in the same solvents (^13^C NMR = 100 MHz). Mass spectra (electrospray in positive mode) were recorded using reverse phase high pressure liquid chromatography-coupled mass spectrometry (LC-MS) on an Agilent/HP 1100 or 1200 system fitted with a Waters X-bridge C_18_ 3.5 μm column using acetonitrile-0.1% trifluoroacetic acid in water as mobile phases.

**Synthesis of tenovin-50**

**4-[3-(4-*tert*-Butyl-benzoyl)-ureido]-benzoic acid (2)**

4-*tert*-Butylbenzamide (**1**) (1 g, 5.64 mmol, 2 equiv.) was stirred in 1,2-dichloroethane (10 mL) with molecular sieves under N_2_ atmosphere and a 2 M solution of oxalyl chloride in dichloromethane (4.5 mL, 9.03 mmol, 3.2 equiv.) added. The clear yellow solution was heated at reflux for 2 hours. The reaction mixture was concentrated *in vacuo*, the remaining residue resuspended in dry acetonitrile (8 mL) and 4-aminobenzoic acid (383.9 mg, 2.80 mmol, 1 equiv.) added. A cream precipitate formed immediately and the suspension was heated at reflux for 15 min. The reaction mixture was cooled to rt and the precipitate collected by filtration. Further washing of the collected solid with acetonitrile gave **2** as a cream solid (900.8 mg, 95%) that was not purified further. ^1^H NMR (400 MHz, DMSO-*d_6_*) δ 11.08 (1H, s, NH), 11.01 (1H, s, NH), 7.94 (4H, m, Ar), 7.69 (2H, d, *J* = 8.7 Hz, ArH), 7.55 (2H, d, *J* = 8.6 Hz, ArH), 1.29 (9H, s, (CH_3_)_3_); ^13^C NMR (100 MHz, DMSO-*d_6_*) δ 168.6, 167.0, 156.4, 151.2, 141.9, 130.6, 129.4, 128.4, 125.7, 125.5, 119.2, 34.9, 30.9; LCMS (*m/z*): [M+H]^+^ 341; HRMS (*m/z*): [M]^+^ calcd for C_19_H_21_N_2_O_4_, 341.1423; found, 341.1496.


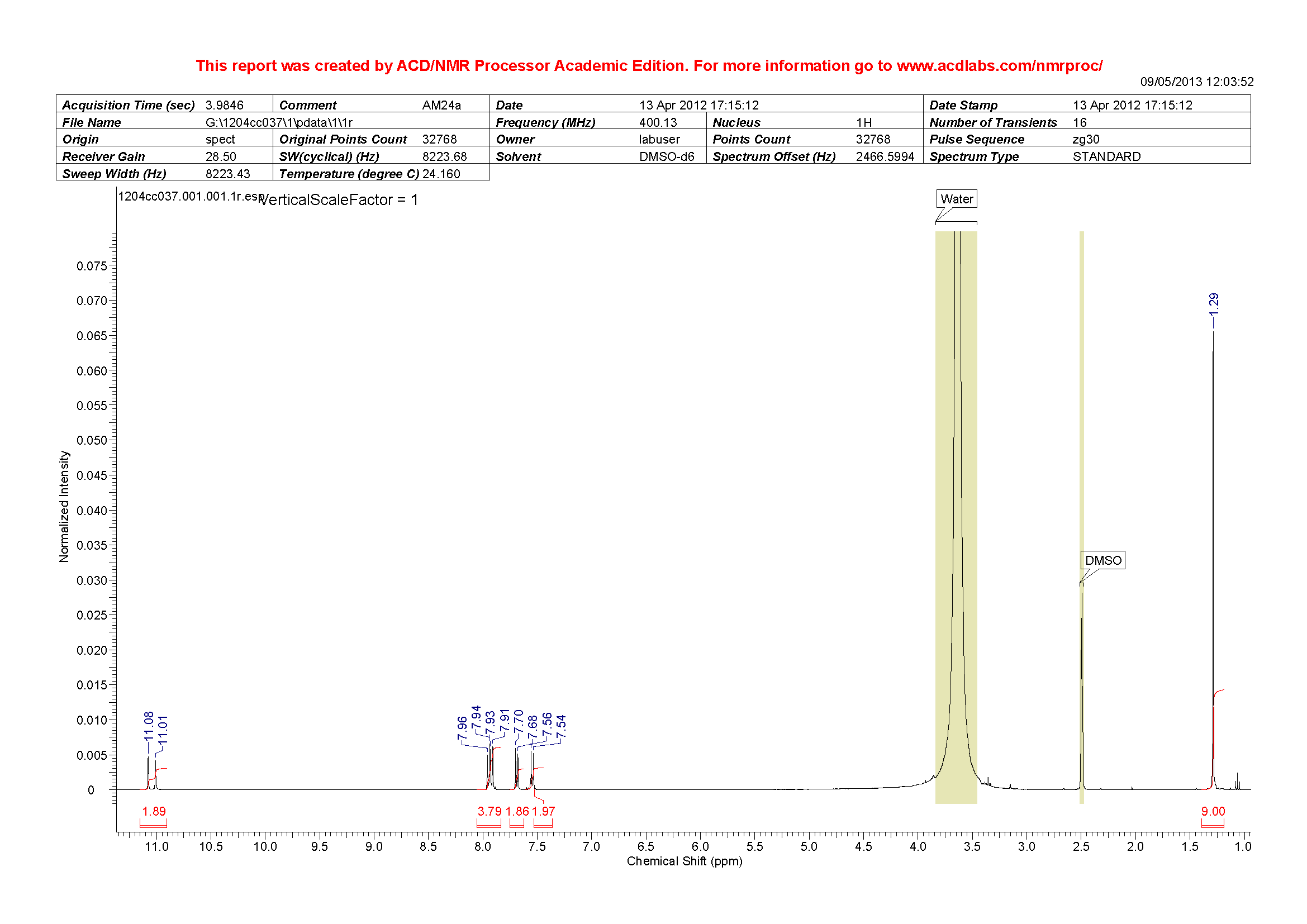


**Supplemental Figure S4a.** ^1^H NMR spectrum of **2**.


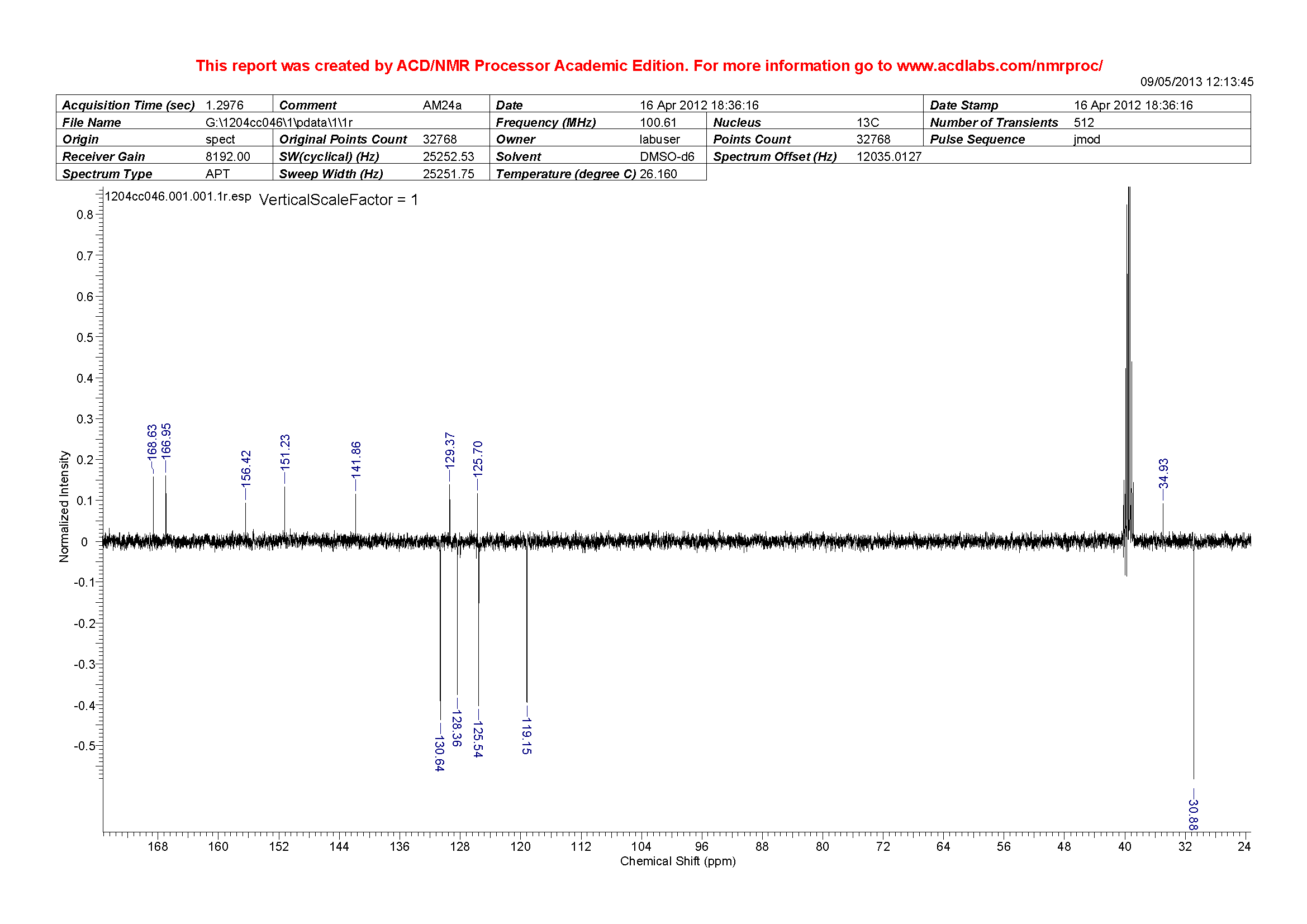


**Supplemental Figure S4b.** ^13^C NMR spectrum of **2**.

**4-[3-(4-*tert*-Butyl-benzoyl)-ureido]-*N*-(4-hydroxyl-butyl)*-*benzamide (tenovin-50-OH)**

**4-[3-(4-*tert*-Butyl-benzoyl)-ureido]-benzoic acid** (400 mg, 1.18 mmol 1 equiv.) was stirred in dichloromethane (8 mL) with triethylamine (326.4 μL, 2.35 mmol, 2 equiv.) under a N_2_ atmosphere and cooled to 0°C. Methyl chloroformate (180 μL, 2.35 mmol, 2 equiv.) was added dropwise. After stirring for 40 min at rt the reaction mixture was cooled to 0°C, 4-aminobutanol (288.8 μL, 3.13 mmol, 2.7 equiv.) added dropwise and the reaction mixture stirred at rt for 16 h. The solvent was removed *in vacuo* and the resulting solid stirred in 2 M aqueous sodium hydroxide. The white precipitate was collected via filtration and washed with water to give **tenovin-50-OH** (147 mg, 30%) as a white solid, which was used without further purification. ^1^H NMR (400 MHz, DMSO-*d_6_*) δ 11.07 (1H, br s, NH), 11.00 (1H, br s, NH), 8.39 (1H, t, *J* = 5.5 Hz, NH), 8.00 (2H, d, *J* = 8.6 Hz, ArH), 7.85 (2H, d, *J* = 8.8 Hz, ArH), 7.67 (2H, d, *J* = 8.8 Hz, ArH), 7.57 (2H, d, *J* = 8.5 Hz, ArH), 4.41 (1H, *t* = 5.0 Hz, OH), 3.41 (2H, t, *J* = 6.5 Hz, CH_2_), 3.27 (2H, m, CH_2_), 1.54 (2H, m, CH_2_), 1.48 (2H, m, CH_2_), 1.32 (9H, s, (CH_3_)_3_); ^13^C NMR (100 MHz, DMSO-*d_6_*) δ 169.2, 165.5, 155.4, 153.0, 140.9, 131.0, 129.0, 128.3, 128.1, 125.2, 118.6, 60.5, 34.8, 31.0, 30.9, 30.0, 25.9 ; LCMS (*m/z*): [M+H]^+^ 412; HRMS (*m/z*): [M]^+^ calcd for C_23_H_30_N_3_O_4_, 412.2158; found, 412.2231.


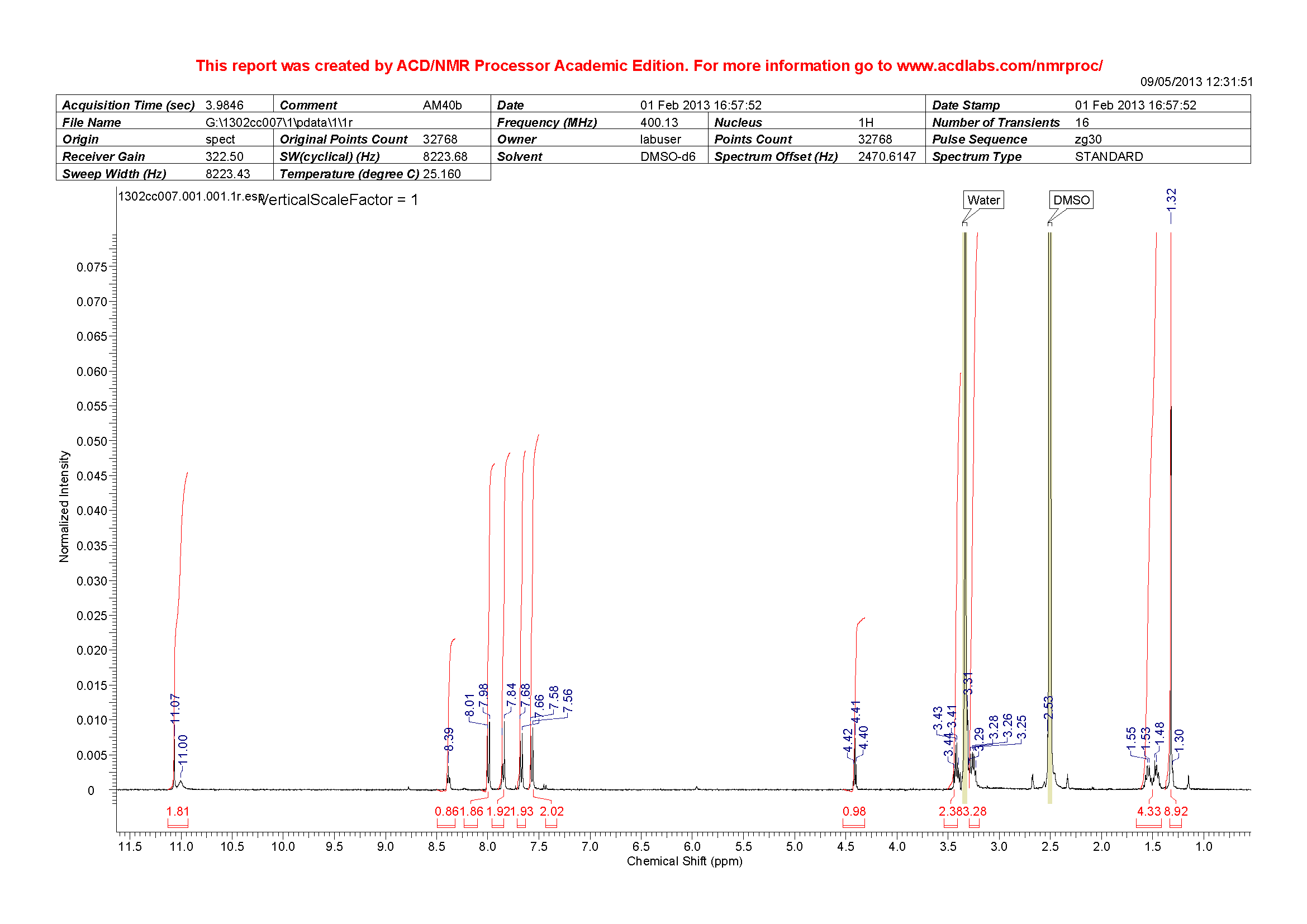


**Supplemental Figure S5a.** ^1^H NMR spectrum of **tenovin-50-OH**.


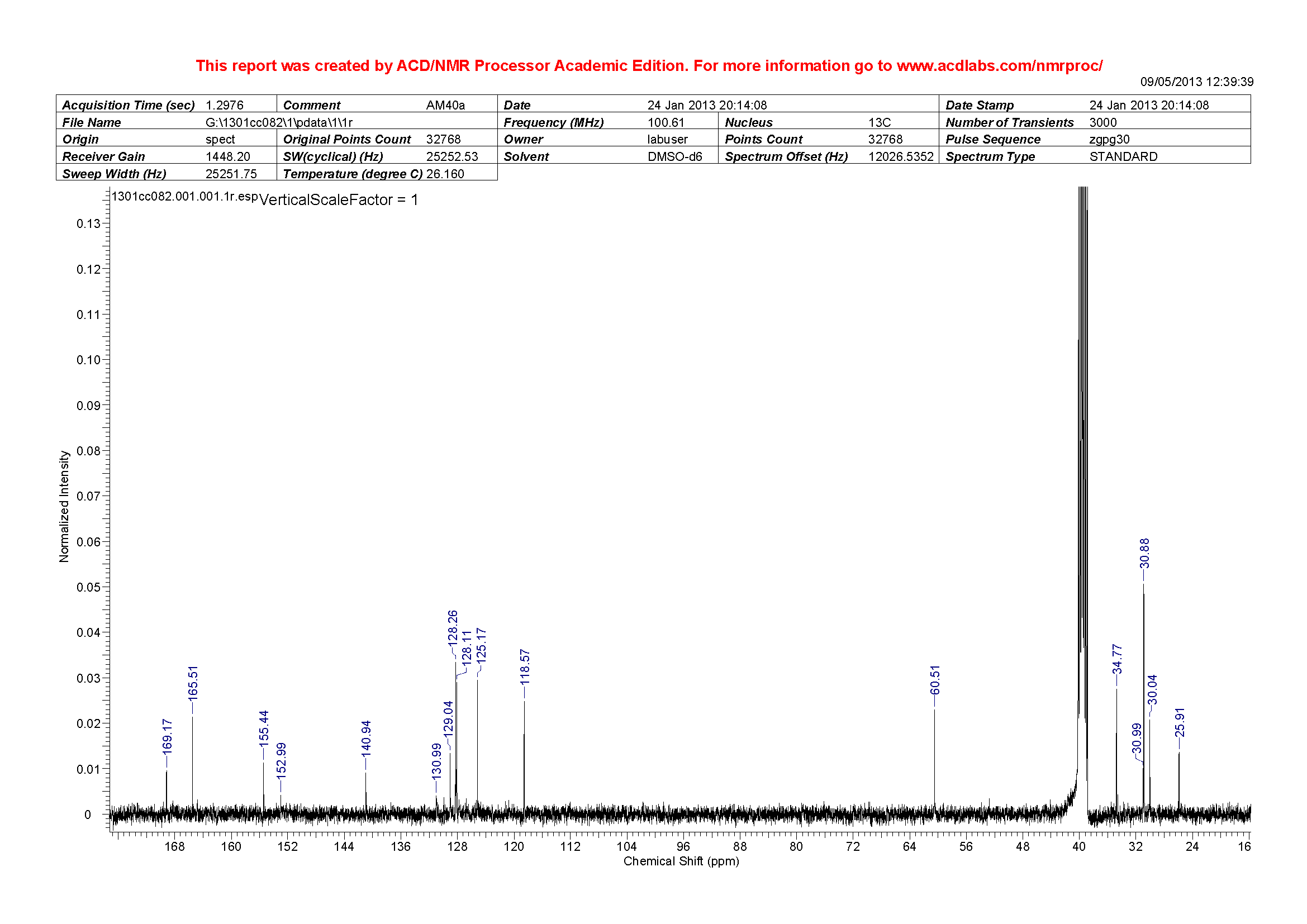


**Supplemental Figure S5b.** ^13^C NMR spectrum of **tenovin-50-OH**.

***N*-(4-Bromo-butyl)-4-[3-(4-*tert*-butyl-benzoyl)-ureido]-benzamide (4)**

**Tenovin-50-OH** (147 mg, 0.36 mmol, 1 equiv.) was stirred in dichloromethane (6 mL) and carbon tetrabromide (130.4 mg, 0.39 mmol, 1.08 equiv.) added. The white suspension was cooled to 0°C and triphenylphosphine (103.2 mg, 0.39 mmol, 1.08 equiv.) added slowly. The resulting mixture was left to warm to room temperature and stirred for 16 hours. The solvent was removed *in vacuo* to give the crude product. Column chromatography eluting with hexanes/ethyl acetate (1:1) afforded **4** (65 mg, 38%) as a white solid. ^1^H NMR (400 MHz, DMSO-*d_6_*) δ 11.07 (1H, s, NH), 11.04 (1H, br s, NH), 8.44 (1H, t, *J* = 5.7 Hz, NH), 7.99 (2H, d, *J =* 8.5 Hz, ArH), 7.85 (2H, d, *J* = 8.8 Hz, ArH), 7.68 (2H, d, *J* = 8.8 Hz, ArH), 7.57 (2H, d, *J* = 8.5 Hz, ArH), 3.58 (2H, t, *J* = 6.8 Hz, CH_2_), 3.27 (2H, m, CH_2_), 1.85 (2H, m, CH_2_), 1.65 (2H, m, CH_2_), 1.32 (9H, s, (CH_3_)_3_); ^13^C NMR (100 MHz, DMSO-*d_6_*) δ 168.5, 165.5, 156.3, 151.1, 140.2, 129.6, 129.4, 128.3, 128.2, 125.5, 119.0, 38.2, 34.9, 34.8, 30.8, 29.8, 27.9; LCMS (*m/z*): [M+H]^+^ 474; HRMS (*m/z*): [M]^+^ calcd for C_23_H_29_N_3_O_3_Br, 474.1314; found, 474.1387.


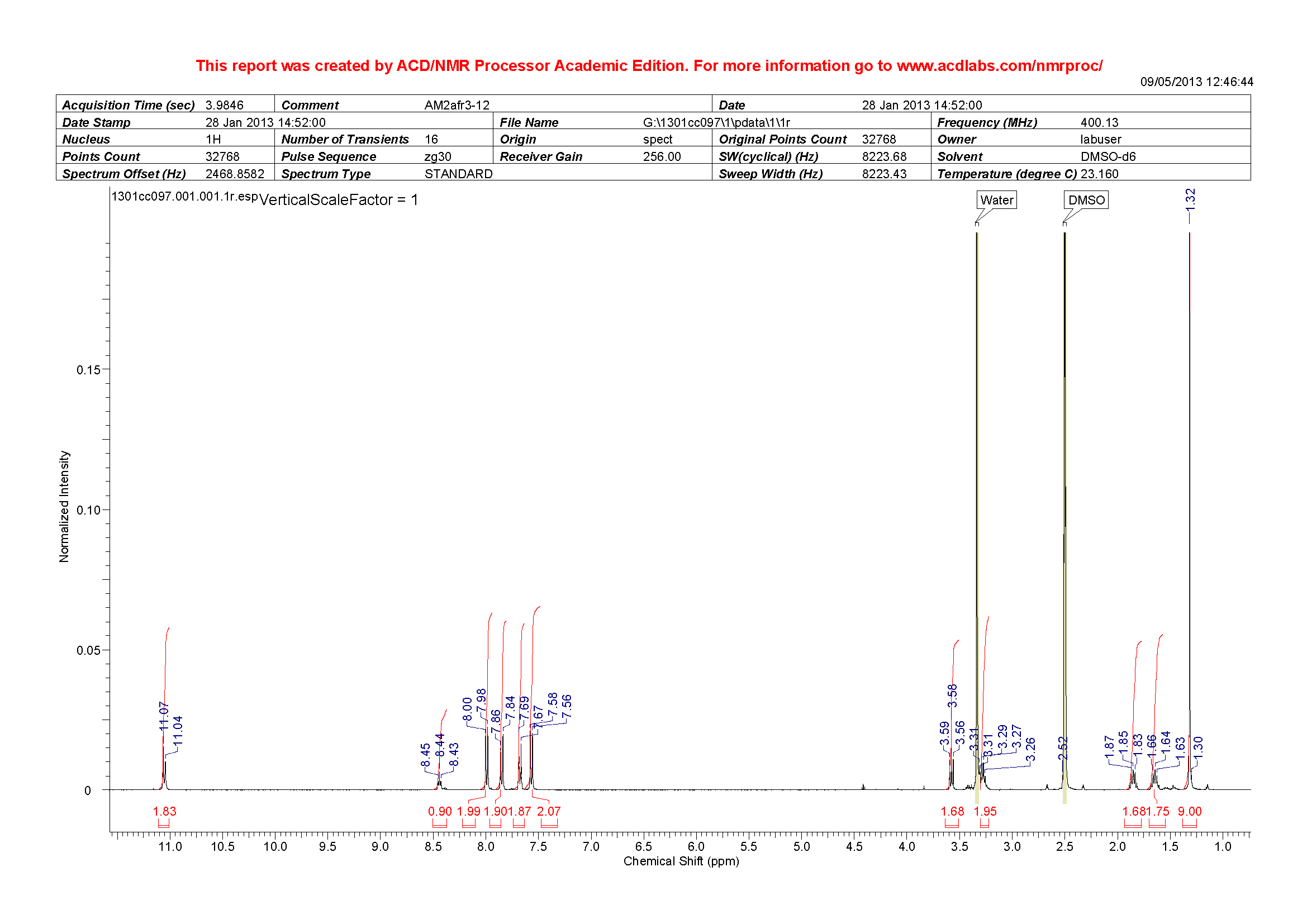


**Supplemental Figure S6a.** ^1^H NMR spectrum of **4**.


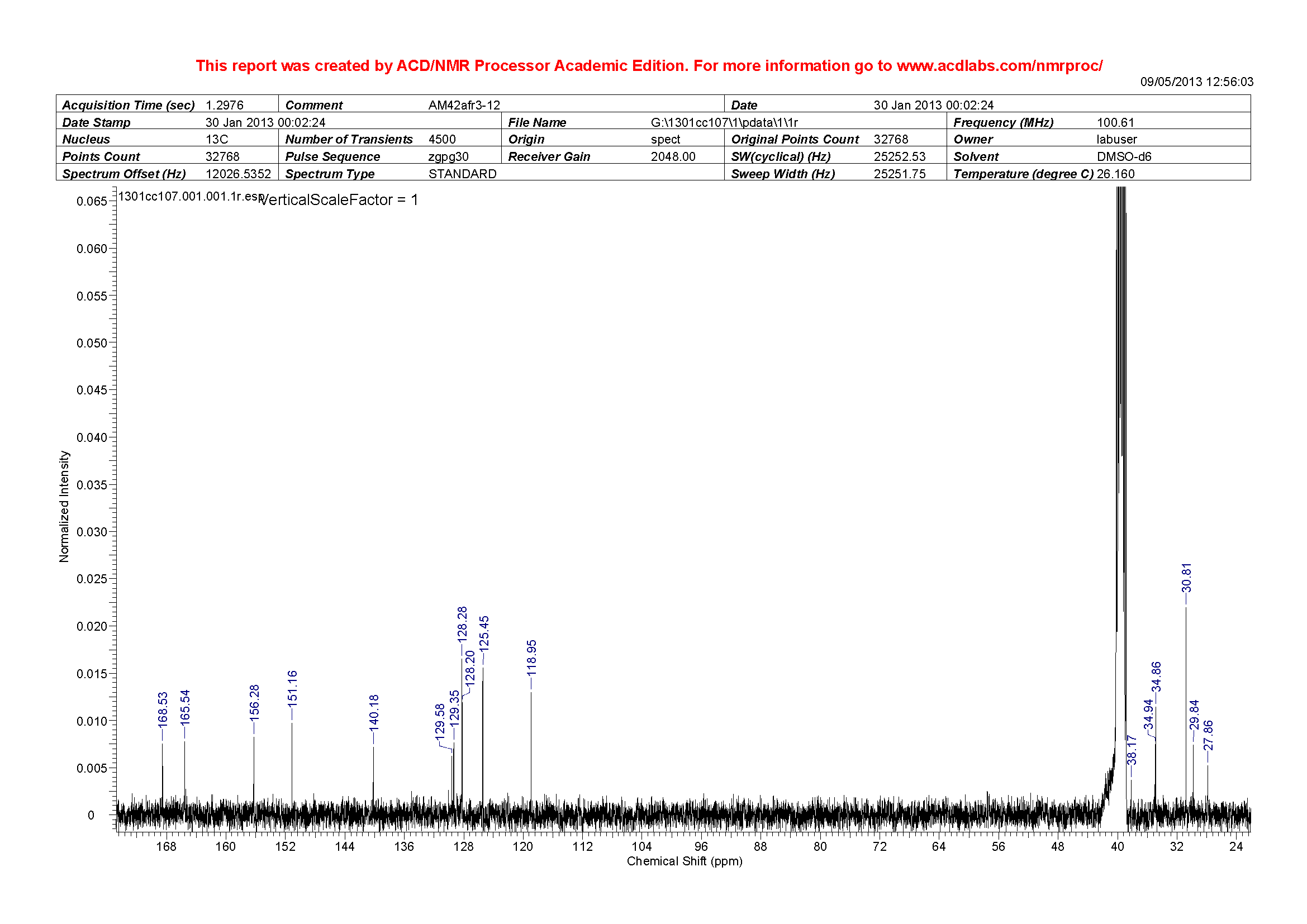


**Supplemental Figure S6b.** ^13^C NMR spectrum of **4**.

**4-[3-(4-*tert*-Butyl-benzoyl)-ureido]-*N*-(4-dimethylamino-butyl)-benzamide hydrochloride (tenovin-50)**

Compound **4** (65 mg, 0.14 mmol was stirred in dichloromethane (4 mL), 40% aqueous dimethylamine (1.5 mL) and water (3 mL) at room temperature for 16 h. The reaction mixture was diluted with dichloromethane (10 mL) and washed with water (1 x 10 mL), 1 M aqueous sodium hydroxide (1 x 10 mL), and saturated brine (1 x 10 mL). The organic phase was dried (MgSO_4_) and the solvent removed *in vacuo* to give a yellow oil which was dissolved in acetone and treated with 2 M hydrochloric acid in diethyl ether dropwise until a precipitate appeared. **Tenovin-50** (20 mg, 31%) was isolated via filtration as a white solid. ^1^H NMR (400 MHz, DMSO-*d_6_*) δ 11.08 (1H, br s, NH), 11.04, br s, NH), 10.16 (1H, br s, NH), 8.52 (1H, br m, NH), 8.00 (2H, d, *J* = 8.0 Hz, ArH), 7.87 (2H, d, *J* = 8.3 Hz, ArH), 7.68 (2H, d, *J* = 8.3 Hz, ArH), 7.56 (2H, d, *J* = 8.1 Hz, ArH), 3.28 (2H, br m, CH_2_), 3.05 (2H, br m, CH_2_), 2.73 (6H, d, *J* = 4.5 Hz, N(CH_3_)_2_), 1.69 (2H, br m, CH_2_), 1.55 (2H, br m, CH_2_), 1.31 (9H, s, (CH_3_)_3_); ^13^C NMR (100 MHz, DMSO-d6) δ 168.3, 165.6, 156.2, 151.1, 140.3, 129.5, 129.4, 128.3, 128.2, 125.4, 118.8, 56.2, 42.0, 38.4, 34.9, 30.8, 26.2, 21.2; LCMS (*m/z*): [(M-HCl)+H]^+^ 439; HRMS (*m/z*): [M]^+^ calcd for C_25_H_35_N_4_O_3_, 439.2709; found, 439.2704.


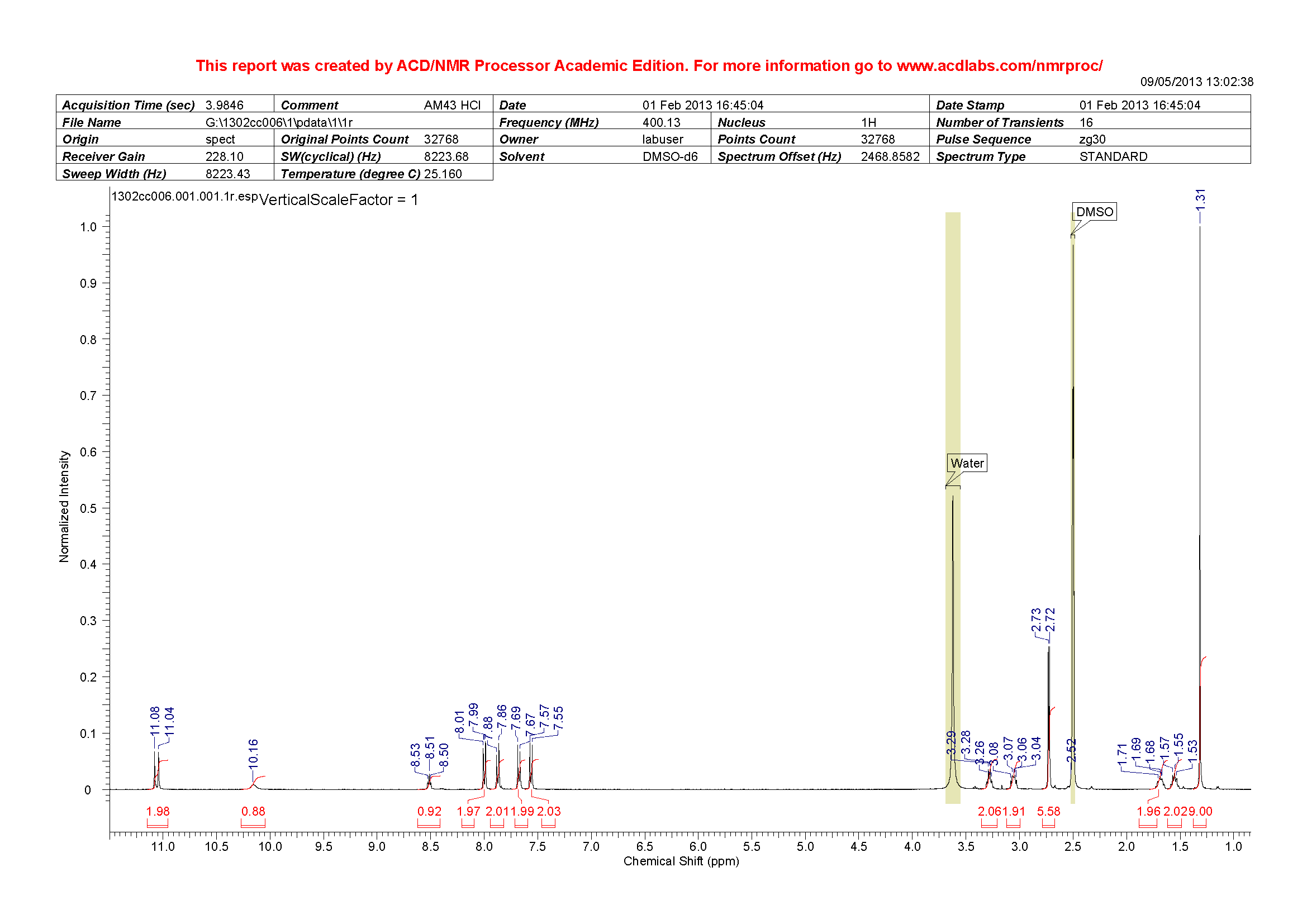


**Supplemental Figure S7a.** ^1^H NMR spectrum of **tenovin-50**.


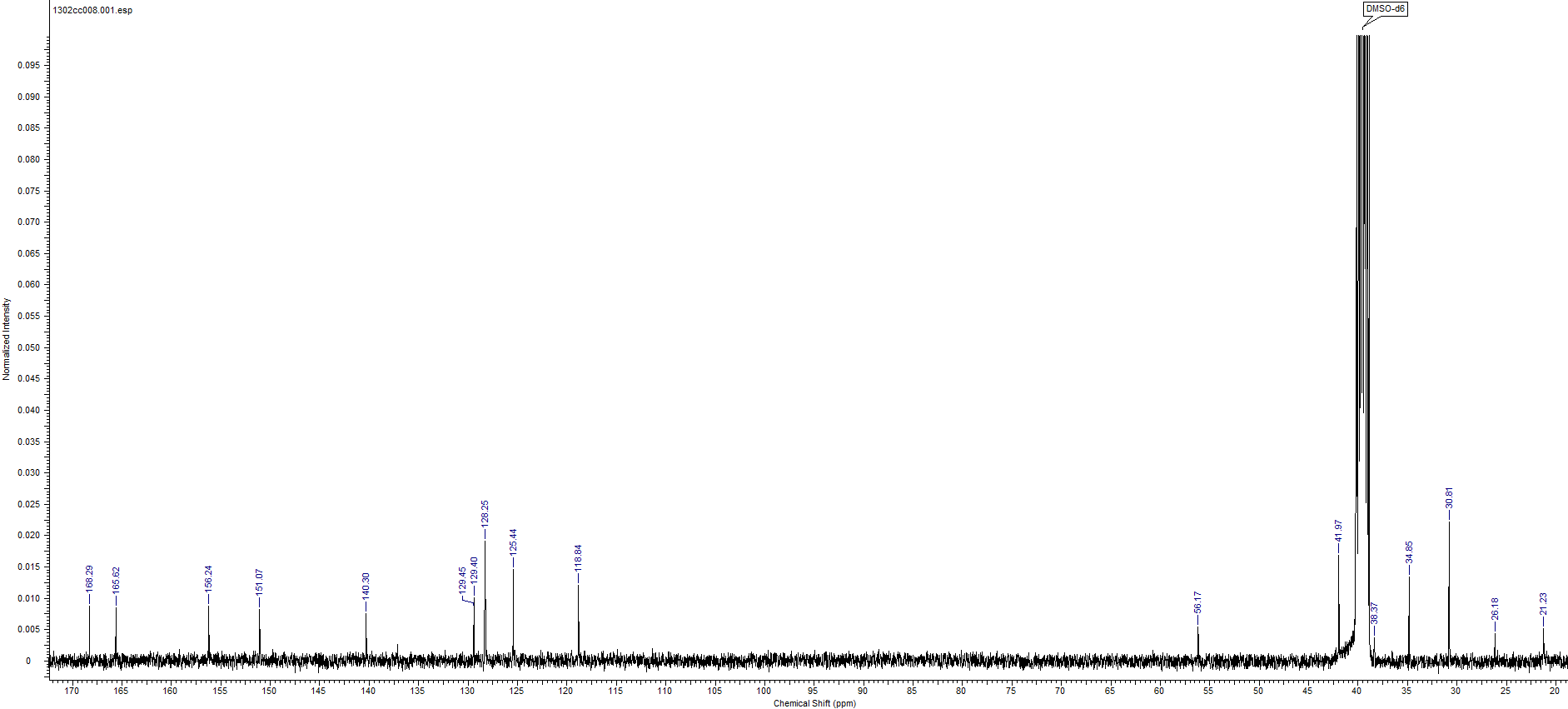


**Supplemental Figure S7b.** ^13^C NMR spectrum of **tenovin-50**.

**Synthesis of tenovin-39-OH and tenovin-51**

**4-[3-(4-*tert*-Butyl-benzoyl)-thioureido]-benzoic acid (6)**

4-*tert*-Butyl benzoyl chloride (**5**) (924 μL, 5.08 mmol, 2 equiv.) was stirred in dry acetone (10 mL) with sodium thiocyanate (410 mg, 5.08 mmol, 2 equiv.) at room temperature for 16 h. To the resulting white suspension was added 4-aminobenzoic acid (348.3 mg, 2.54 mmol, 1 equiv.) as a solution in acetone (3 mL) and the mixture stirred at room temperature for 30 min. Removal of the solvent *in vacuo* gave the crude product, which was resuspended in dichloromethane. The white precipitate was collected by filtration to afford **6** (1.4 g, 77%) as a white solid that was used without further purification. ^1^H NMR (400 MHz, DMSO-*d_6_*) δ 12.98 (1H, br s, OH), 12.86 (1H, s, NH), 11.56 (1H, s, NH), 7.94 (6H, m, ArH), 7.58 (2H, d, *J* = 8.6 Hz, ArH), 1.32 (9H, s, (CH_3_)_3_); ^13^C NMR (100 MHz, DMSO-*d_6_*) ) δ 13C NMR (100 MHz, DMSO-d6) δ 179.0, 168.0, 166.7, 156.4, 141.9, 129.9, 129.2, 128.7, 128.0, 125.3, 123.5, 34.9, 30.8; LCMS (*m/z*): [M+H]^+^ 357; HRMS (*m/z*): [M]^+^ calcd for C_19_H_21_N_2_O_3_S, 357.1194; found, 357.1267.


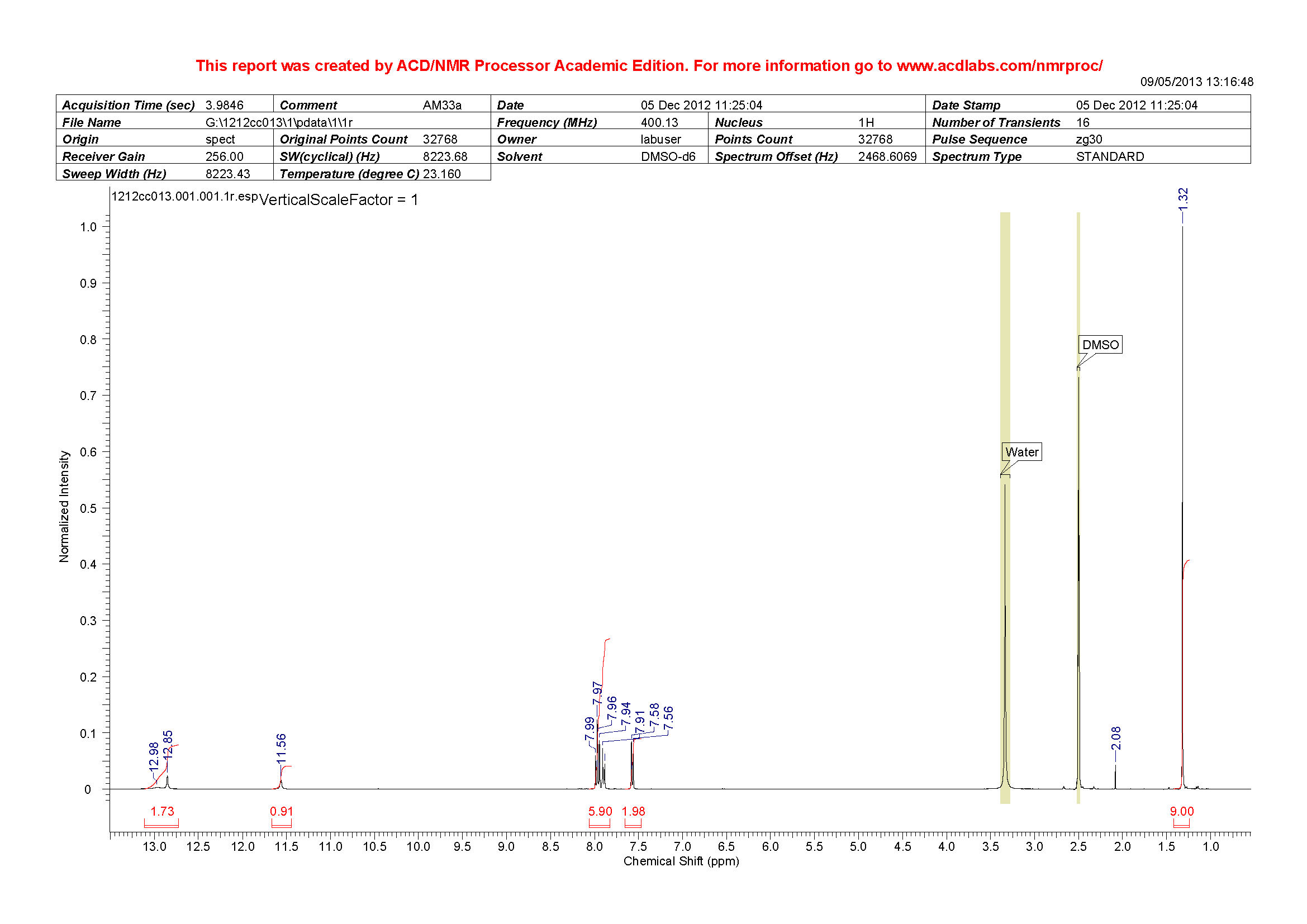


**Supplemental Figure S8a.** ^1^H NMR spectrum of **6**.


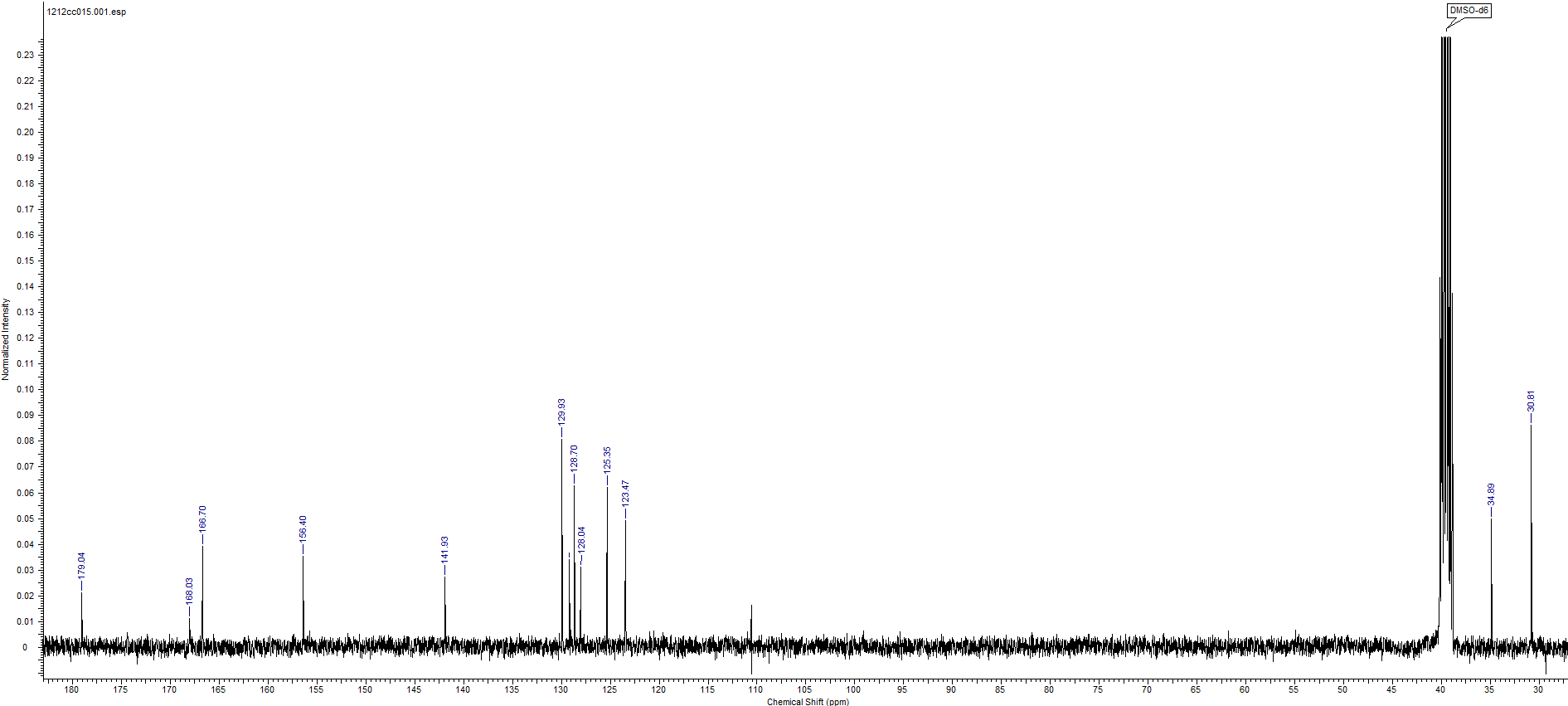


**Supplemental Figure S8b.** ^13^C NMR spectrum of **6**.

**4-[3-(4-*tert*-Butyl-benzoyl)-thioureido]-*N*-(4-hydroxy-butyl)-benzamide (tenovin-39-OH)**

Compound **6** (300 mg, 0.84 mmol, 1 equiv.) was stirred in dichloromethane (5 mL) with triethylamine (236.5 μL, 1.68 mmol, 2 equiv.) under N_2_ atmosphere and cooled to 0°C. Methyl chloroformate (129.8 μL, 1.68 mmol, 2 equiv.) was added dropwise and the mixture warmed to room temperature. After stirring for 4 hours the reaction mixture was cooled to 0°C and 4-aminobutanol (155.2 μL, 1.68 mmol, 2 equiv.) added dropwise before the reaction mixture was stirred at rt for 16 h. The solvent was removed *in vacuo* to give the crude product. Column chromatography eluting with dichloromethane/methanol (19:1) afforded **tenovin-39-OH** which was purified further by resuspending in ethyl acetate and collecting as a white solid via filtration (29 mg, 8%). ^1^H NMR (400 MHz, CDCl_3_) δ 12.86 (1H, s, NH), 9.08 (1H, s, NH), 7.86 (6H, m, ArH), 7.57 (2H, d, *J* = 8.8 Hz), 6.50 (1H, m, NH), 3.76 (2H, m, CH_2_), 3.54 (2H, m, CH_2_), 1.74 (4H, m, (CH_2_)_2_), 1.38 (9H, s, (CH_3_)_3_) ; ^13^C NMR (100 MHz, CDCl_3_) δ 178.3, 166.9, 166.7, 158.0, 140.3, 132.6, 128.4, 127.7, 127.5, 126.3, 123.4, 62.5, 39.8, 35.3, 31.0, 29.8, 26.3; LCMS (*m/z*): [M+H]^+^ 428; HRMS (*m/z*): [M]^+^ calcd for C_23_H_30_N_3_O_3_S, 428.1929; found, 428.2002.


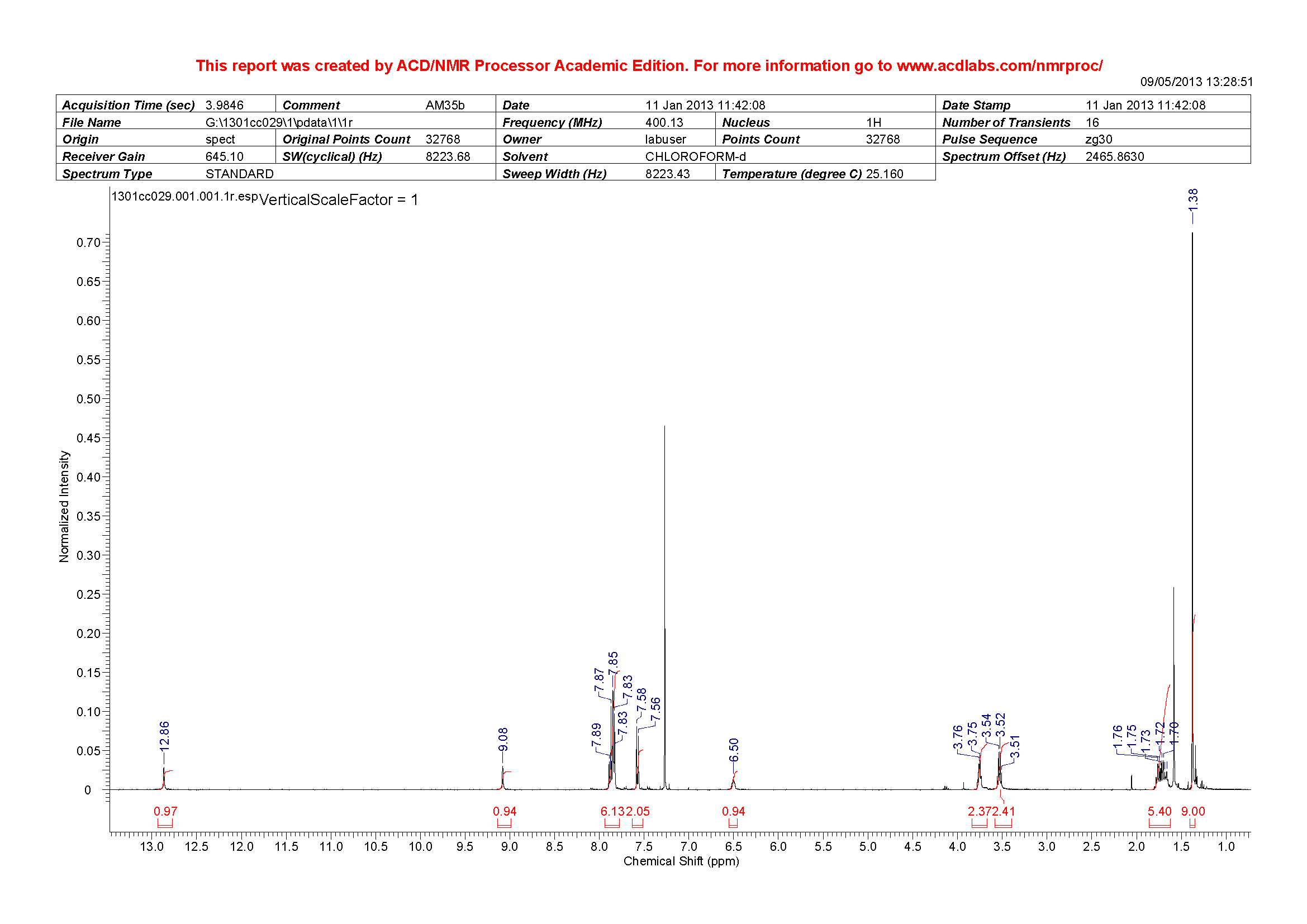


**Supplemental Figure S9a.** ^1^H NMR spectrum of **tenovin-39-OH**.


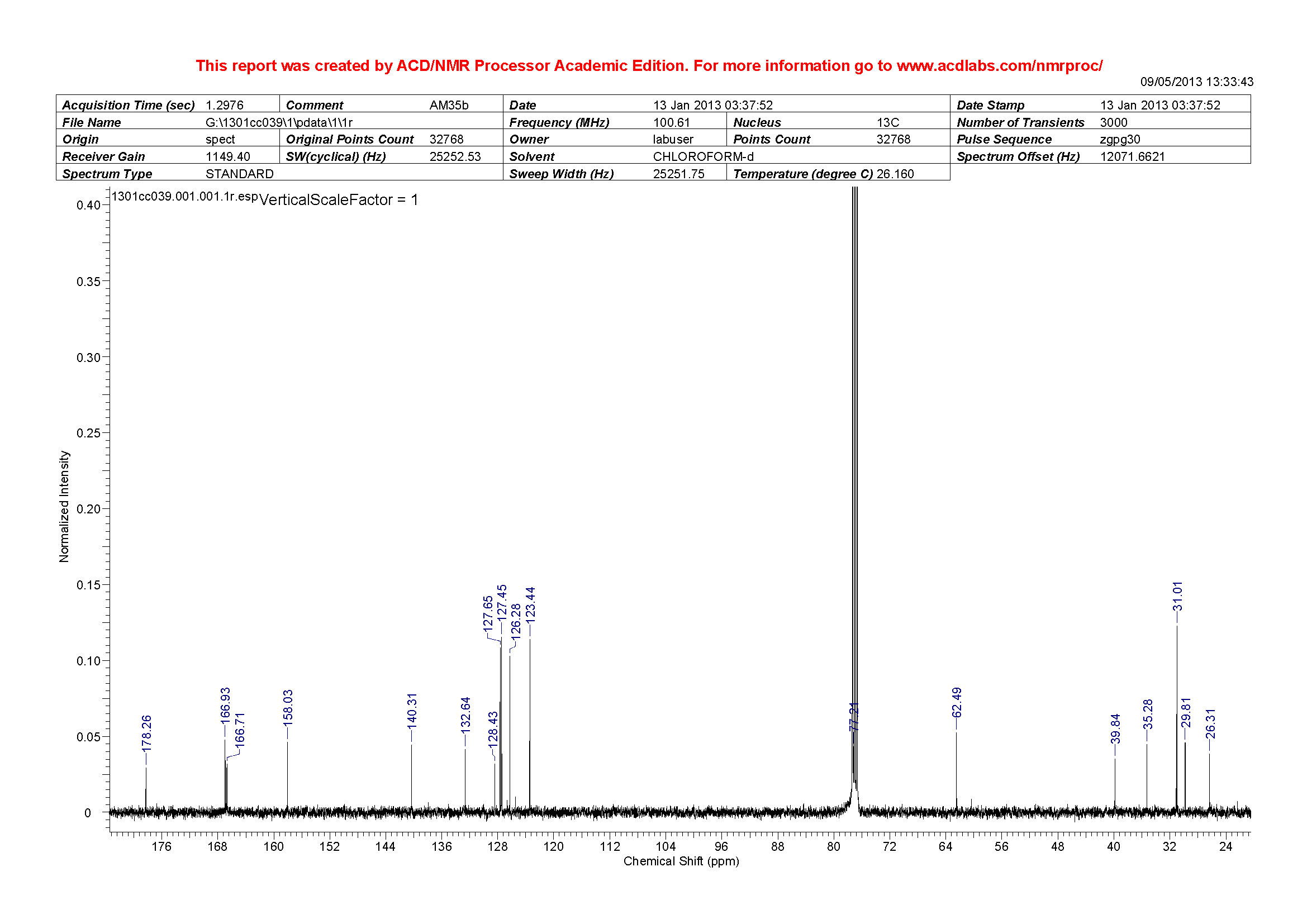


**Supplemental Figure S9b.** ^13^C NMR spectrum of **tenovin-39-OH**.

**Synthesis of tenovin-51**

**{4-[3-(4-*tert*-Butyl-benzoyl)-thioureido]-phenyl}-carbamic acid *tert*-butyl ester (tenovin-51)**

4-*tert*-Butyl benzoyl chloride (**1**) (500 mg, 2.54 mmol, 1 equiv.) was stirred in dry acetone (4 mL) with sodium thiocyanate (205 mg, 2.54 mmol, 1 equiv.) to give a pale yellow suspension which was stirred at room temperature for 16 h. *N*-Boc-*p*-phenylenediamine (529 mg, 2.54 mmol, 1 equiv.) was dissolved in acetone (3 mL) and added slowly to the yellow suspension. After addition of the amine the suspension was concentrated *in vacuo* to give the crude product. The product was resuspended in dichloromethane, filtered, and the filtrate concentrated *in vacuo* to give **tenovin-51** (663 mg, 63%) as a yellow solid that was used without further purification. ^1^H NMR (400 MHz, DMSO-*d_6_*) δ 12.56 (1H, s, NH), 9.09 (1H, s, NH), 7.83 (2H, d, *J* = 8.5 Hz, ArH), 7.64 (2H, d, *J* = 8.8 Hz, ArH), 7.56 (2H, d, *J* = 8.5 Hz, ArH), 7.42 (2H, d, *J* = 8.7 Hz, ArH), 6.59 (1H, s, NH), 1.53 (1H, s, (CH_3_)_3_), 1.37 (1H, s, (CH_3_)_3_); ^13^C NMR (100 MHz, DMSO-*d_6_*) δ 178.3, 166.8, 157.8, 152.6, 137.0, 132.6, 128.7, 127.4, 126.2, 124.9, 118.6, 80.8, 35.3, 31.0, 28.3; LCMS (*m/z*): [M+H]^+^ 428; HRMS (*m/z*): [M]^+^ calcd for C_23_H_30_N_3_O_3_S, 428.1929; found, 428.2002.


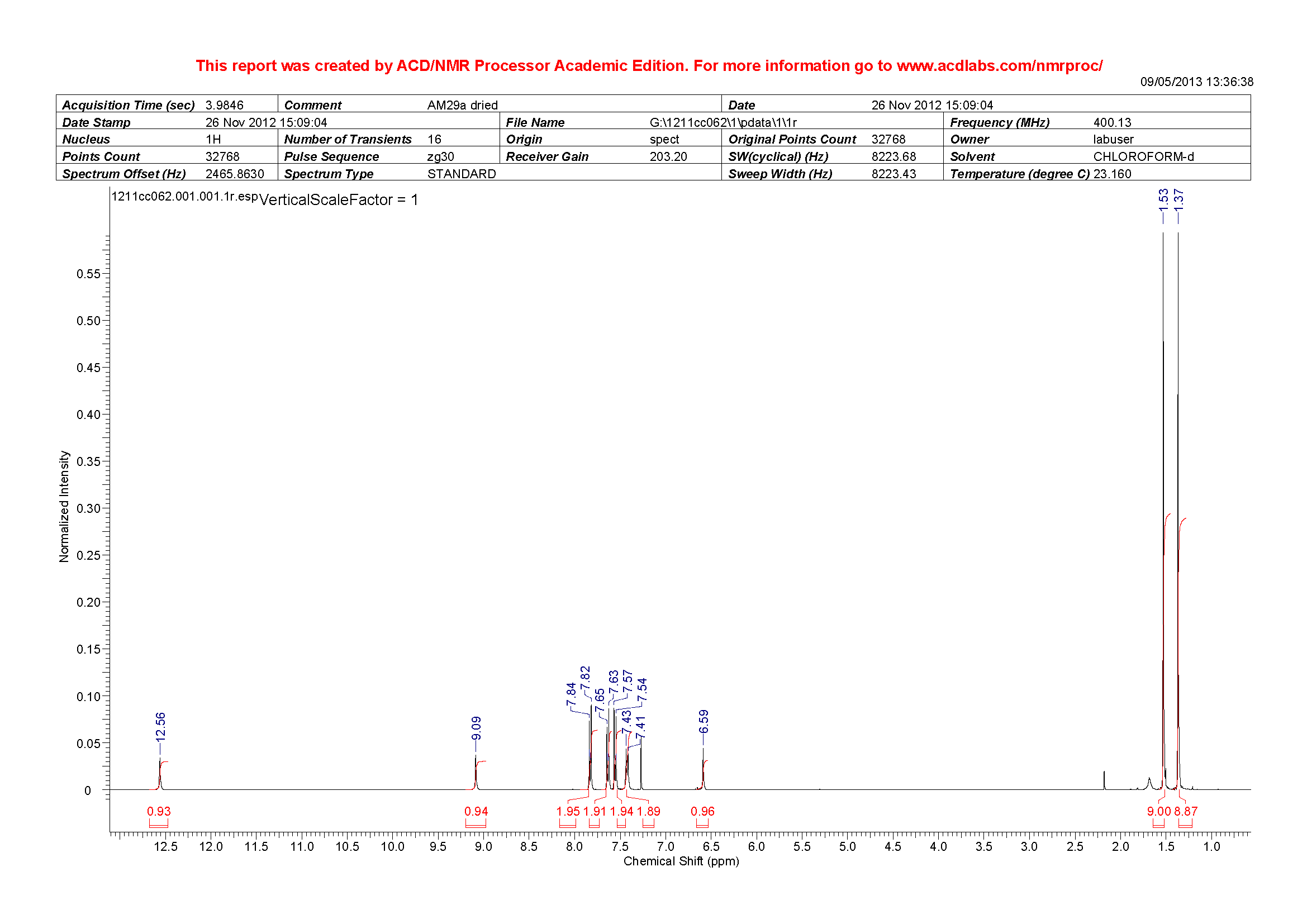


**Supplemental Figure S10a.** ^1^H NMR spectrum of **tenovin-51**.


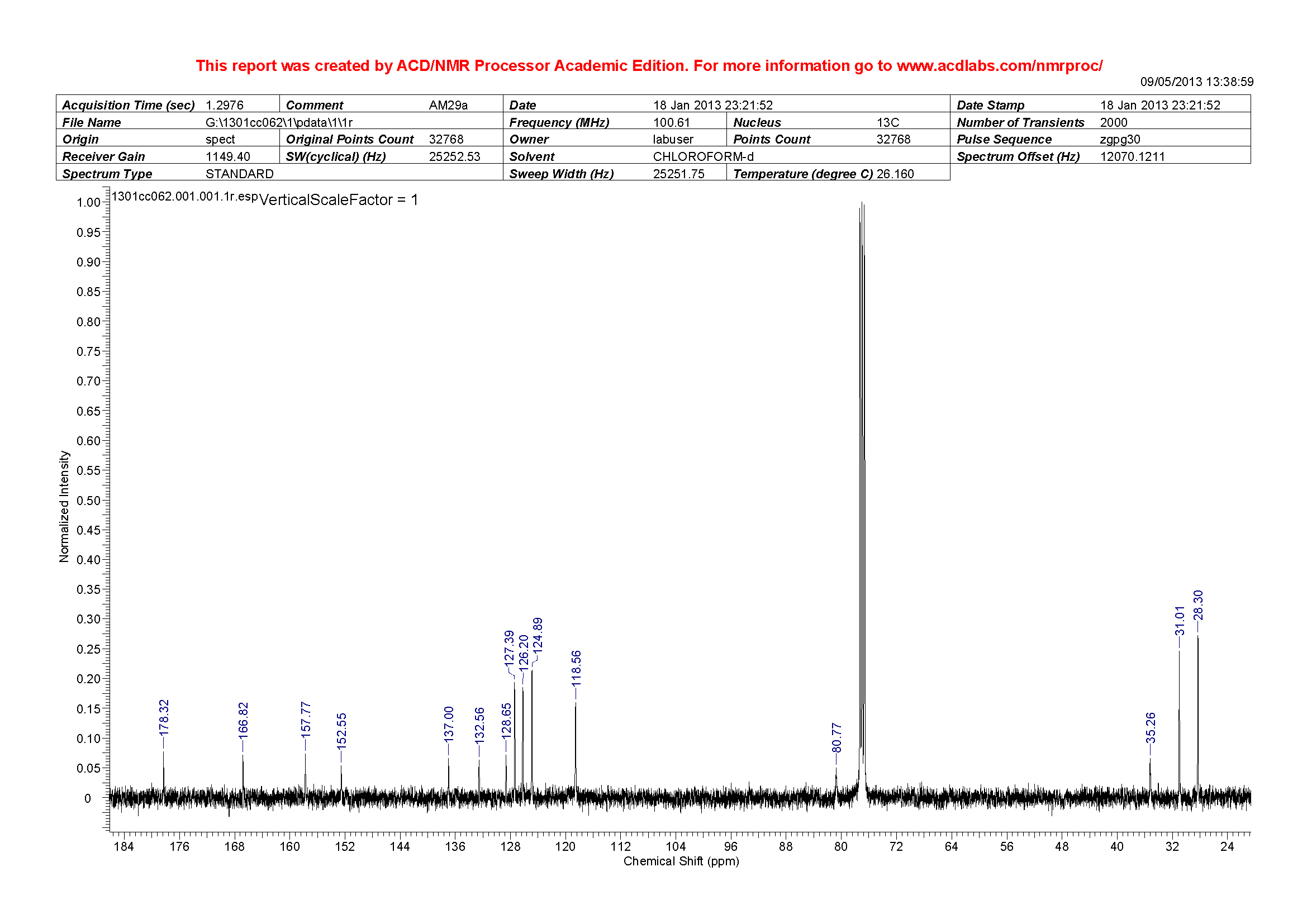


**Supplemental Figure S10b.** ^13^C NMR spectrum of **tenovin-51**.
