# Supplementary material for: Autophagic flux blockage by accumulation of weakly basic tenovins leads to elimination of B-Raf mutant tumour cells that survive vemurafenib
Source: PLoS One. 2018 Apr 23;13(4):e0195956. doi: 10.1371/journal.pone.0195956 (PMC5912769; doi:10.1371/journal.pone.0195956)
Supplement: S3 File — (PDF) [file pone.0195956.s009.pdf]

Western Blot Full Films

Fig 2A – Tenovin 6 and D3 (Top blot left film) – LC3B
Tenovin 1 and 51 (Bottom Blot Right film) – LC3B

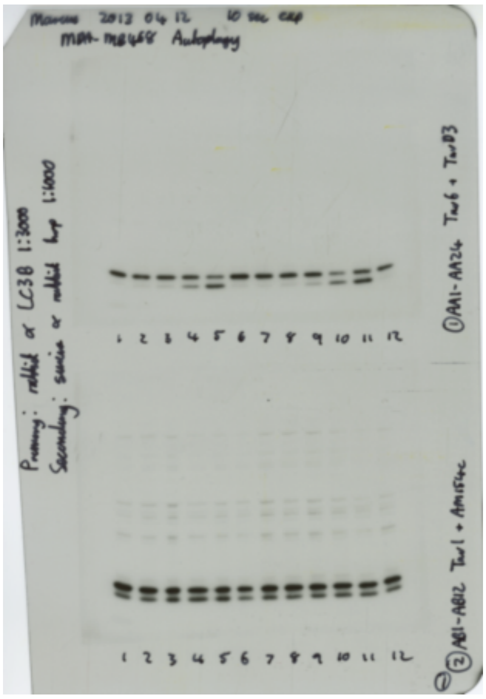

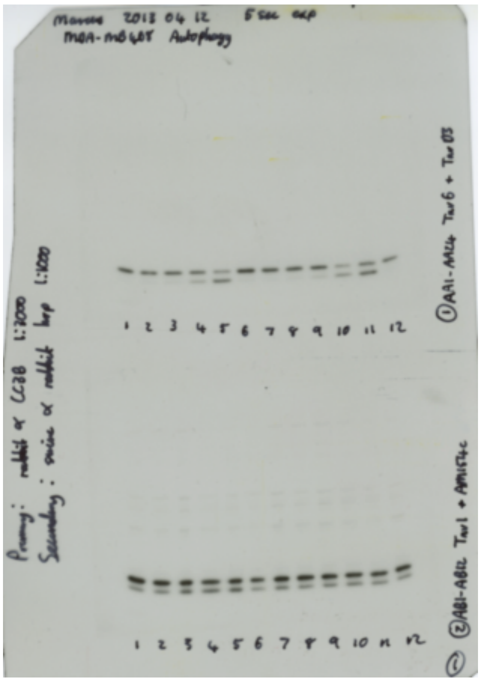

Tenovin 6 and D3 (Top blot) and Tenovin 1 and 51 (bottom) – Alpha Tubulin

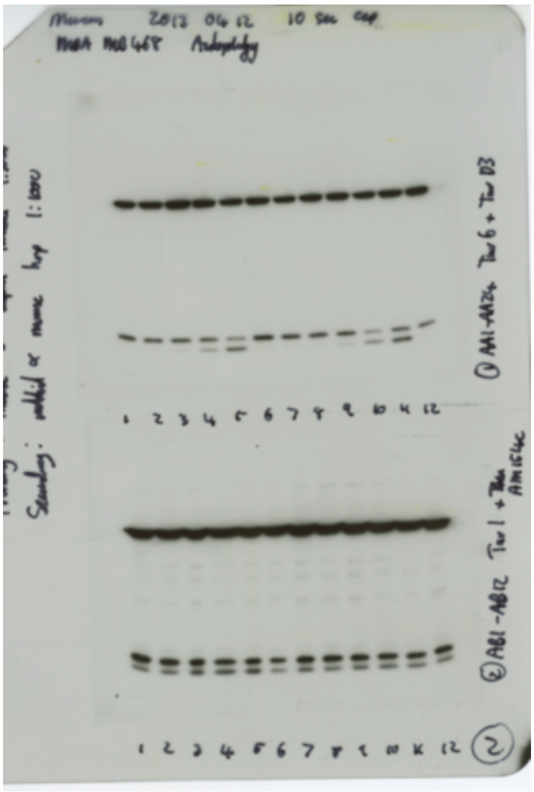

Top Blot	Bottom Blot
**1.** DMSO	**1.** DMSO
**2.** 2.5 µM Tenovin 6	**2.** 2.5 µM tenovin 1
**3.** 5 µM tenovin 6	**3.** 5 µM tenovin 1
**4.** 10 µM tenovin 6	**4.** 10 µM tenovin 1
**5.** 15 µM tenovin 6	**5.** 15 µM tenovin 1
**6.** DMSO	**6.** DMSO
**7.** DMSO	**7.** DMSO
**8.** 2.5 µM tenovin D3	**8.** 2.5 µM tenovin 51
**9.** 5 µM tenovin D3	**9.** 5 µM tenovin 51
**10.** 10 µM tenovin D3	**10.** 10 µM tenovin 51
**11.** 15 µM tenovin D3	**11.** 15 µM tenovin 51
**12.** DMSO	**12.** DMSO

Fig 2A – Tenovin 39 and 39OH – LC3B (left blot) and alpha tubulin (right blot)

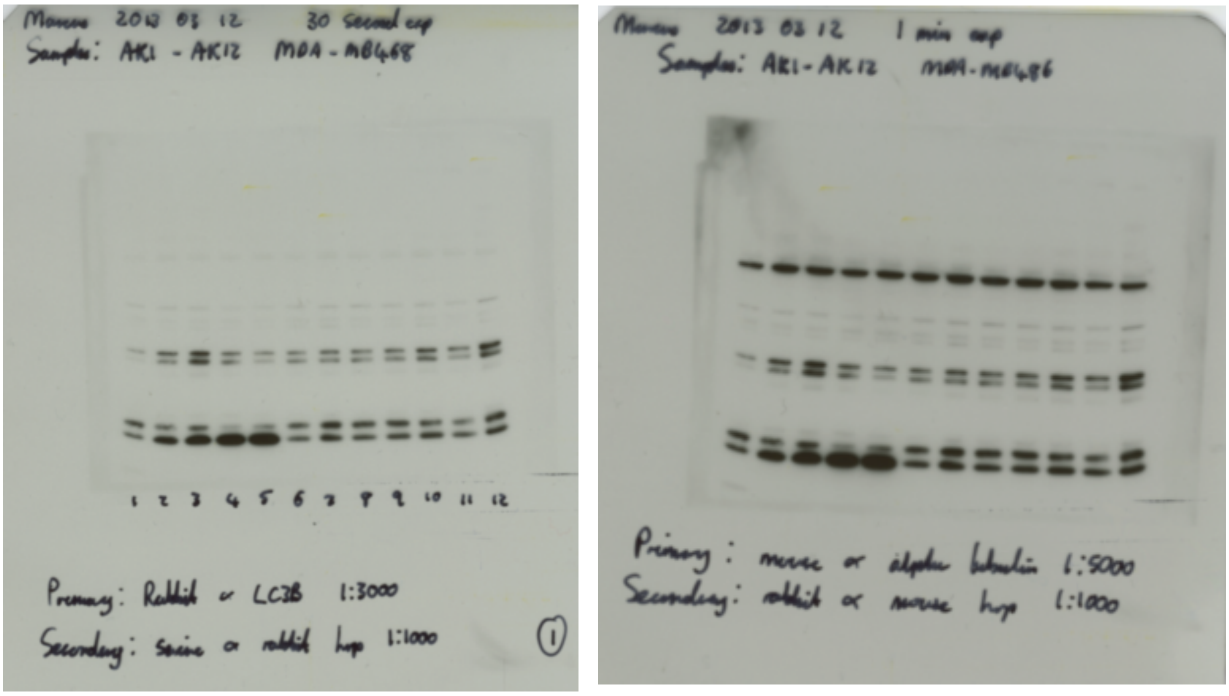

**Samples**
**1.** DMSO
**2.** 2.5 µM Tenovin 39
**3.** 5 µM tenovin 39
**4.** 10 µM tenovin 39
**5.** 15 µM tenovin 39
**6.** DMSO
**7.** DMSO
**8.** 2.5 µM tenovin 39 OH
**9.** 5 µM tenovin 39 OH
**10.** 10 µM tenovin 39 OH
**11.** 15 µM tenovin 39 OH
**12.** DMSO

Fig 2A – Tenovin 50 – LC3B (left blot top panel) and alpha tubulin (right blot top panel)

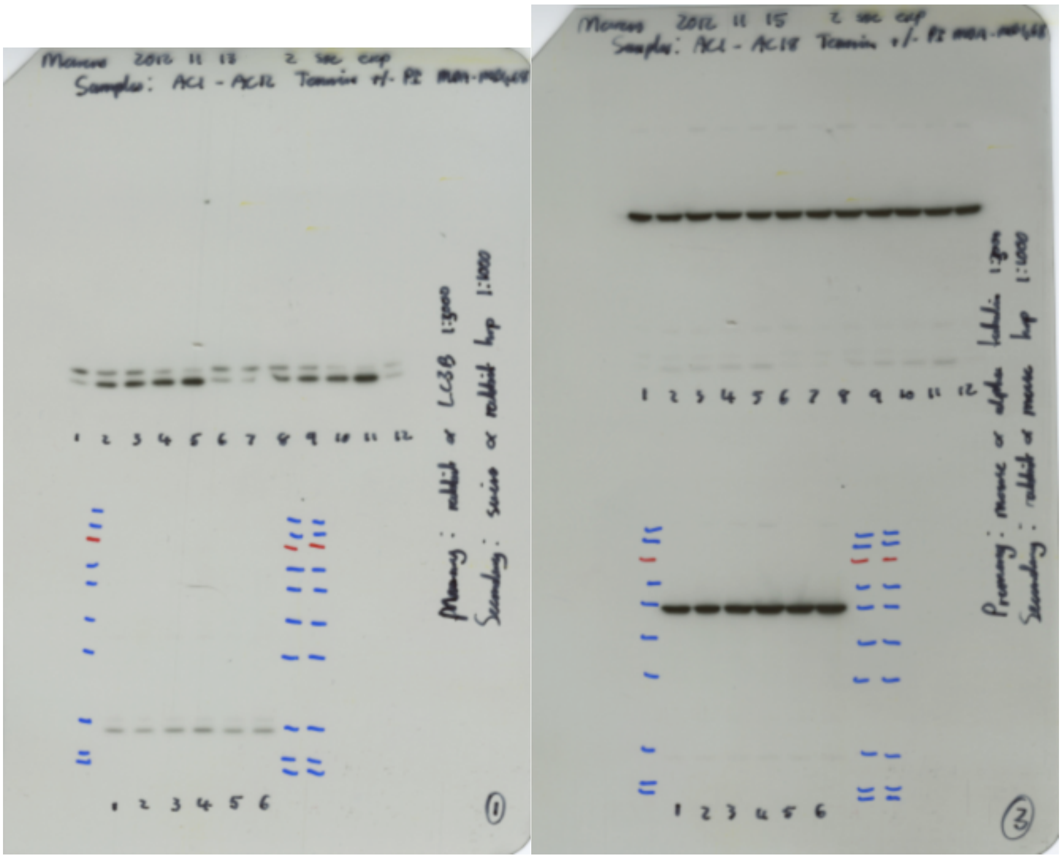

**Samples**
**1.** DMSO
**2.** 2.5 µM Tenovin 50
**3.** 5 µM tenovin 50
**4.** 10 µM tenovin 50
**5.** 15 µM tenovin 50
**6.** DMSO
**7-12** – another experiment

Fig 2A – Tenovin 50 OH – LC3B (left blot) and alpha tubulin (right blot)

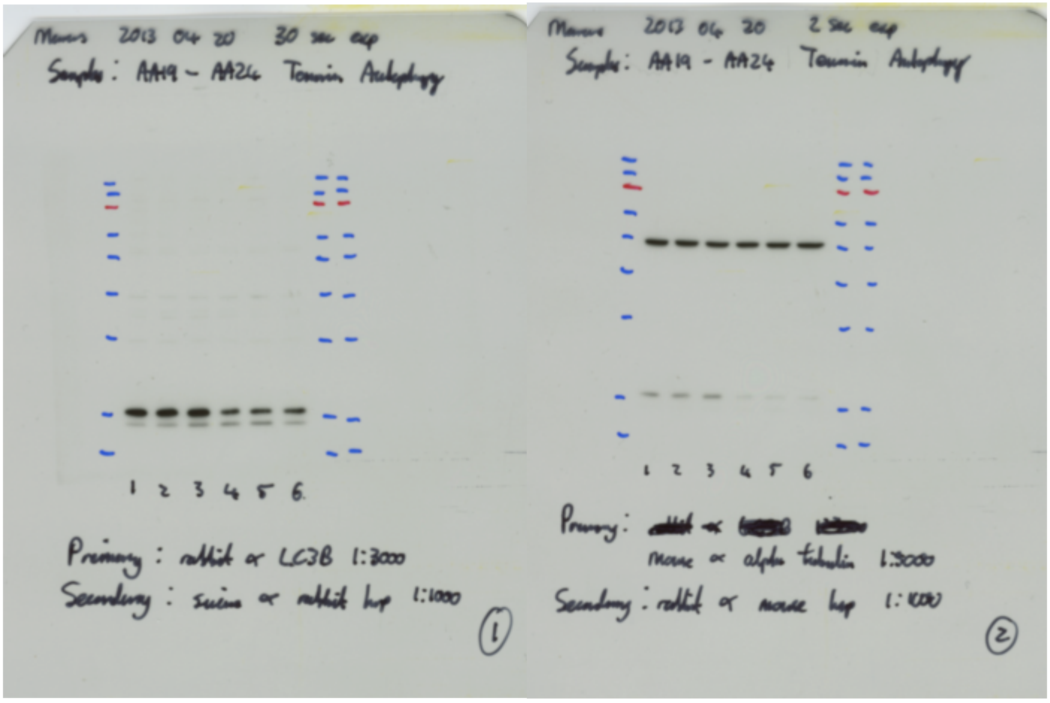

**Samples**
**1.** DMSO
**2.** 5 µM Tenovin 50 OH
**3.** 10 µM tenovin 50 OH
**4.** 15 µM tenovin 50 OH
**5.** 20 µM tenovin 50 OH
**6.** DMSO

Fig 2B – 1 hour treatment blot (LC3B left blot, p62 right blot).

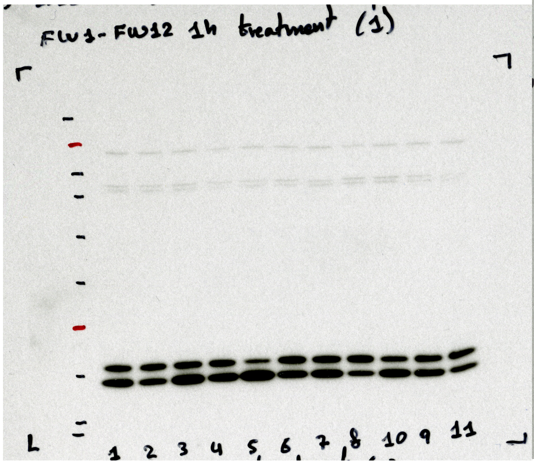

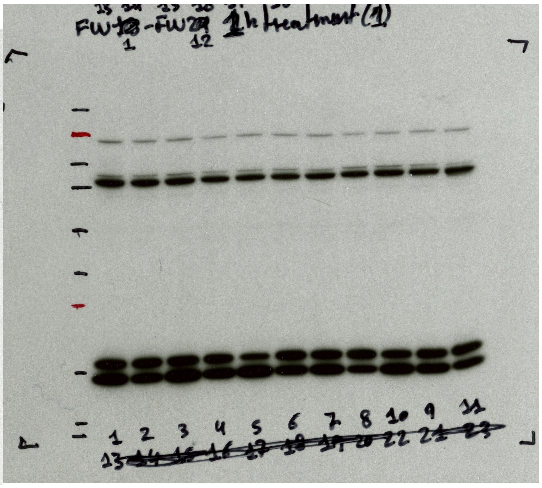

gapdh blot

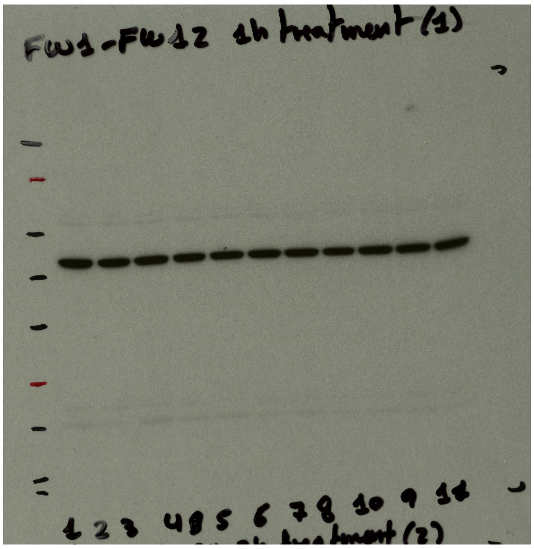

**Samples**
**1.** DMSO
**2.** Tenovin-1
**3.** Tenovin-6
**4.** Tenovin-33
**5.** Tenovin-39
**6.** Tenovin-39-OH
**7.** Tenovin-50
**8.** Tenovin-50-OH
**10.** Chloroquine
**9.** Tenovin-D3
**11.** DMSO

Fig 2B – 2 hour treatment blot (LC3B left blot, p62 right blot).

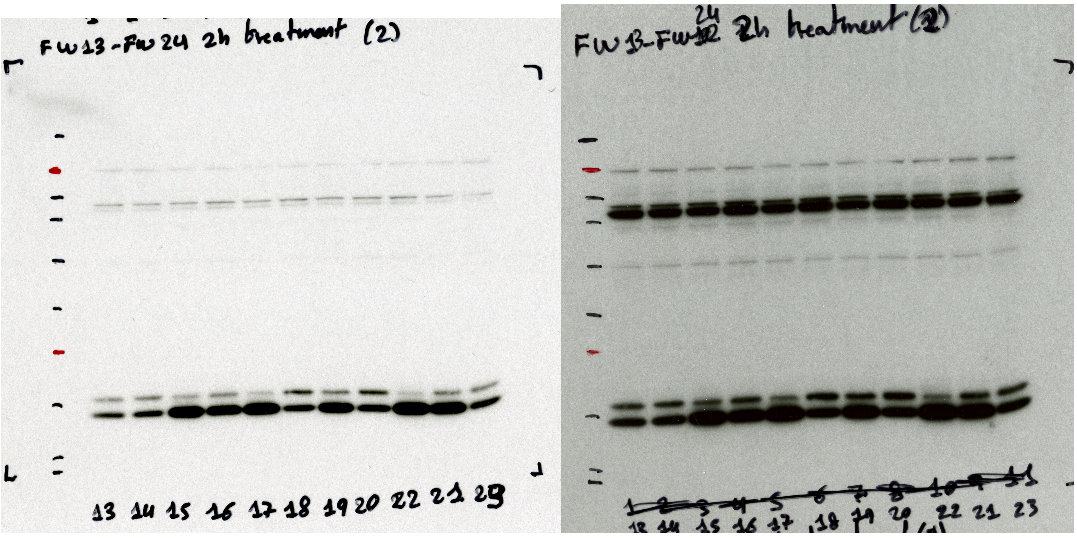

gapdh blot

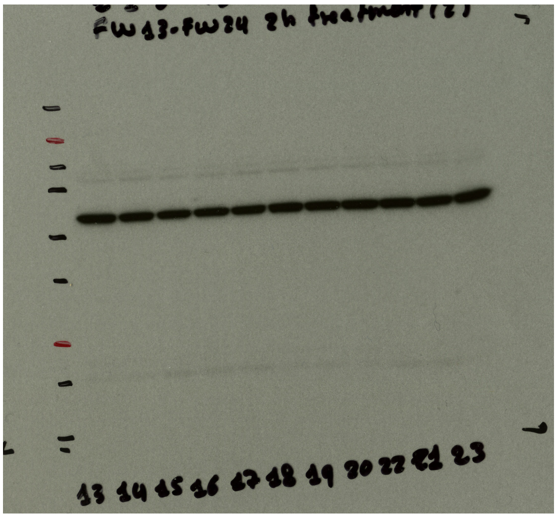

**Samples**
**13.** DMSO
**14.** Tenovin-1
**15.** Tenovin-6
**16.** Tenovin-33
**17.** Tenovin-39
**18.** Tenovin-39-OH
**19.** Tenovin-50
**20.** Tenovin-50-OH
**22.** Chloroquine
**21.** Tenovin-D3
**23.** DMSO

Fig 2B – 4h treatment blot (LC3B left blot, p62 right blot)

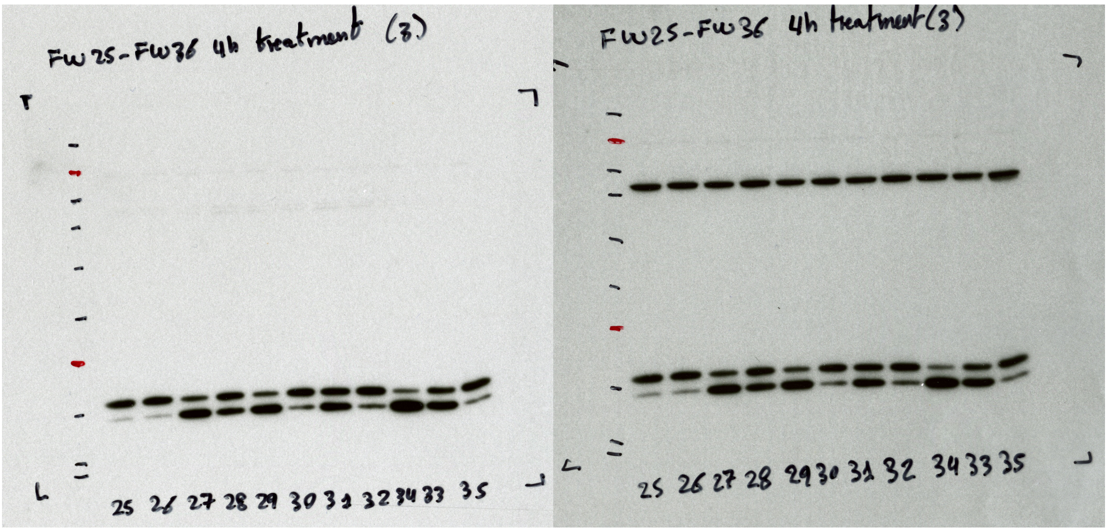

gapdh blot

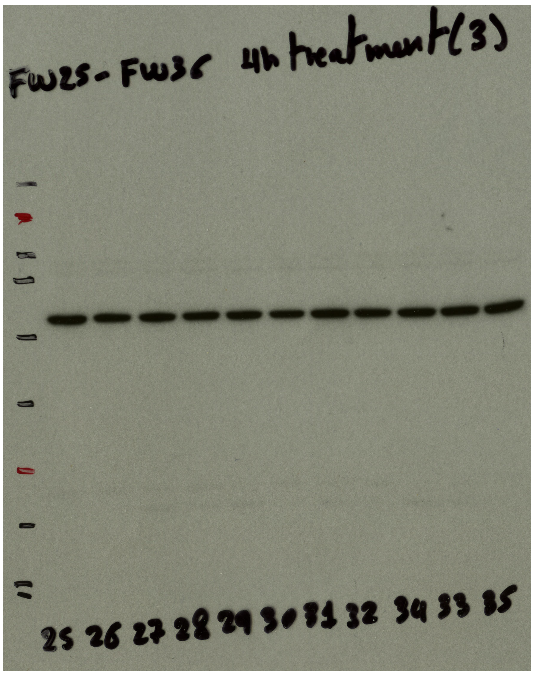

**Samples**
**25.** DMSO
**26.** Tenovin-1
**27.** Tenovin-6
**28.** Tenovin-33
**29.** Tenovin-39
**30.** Tenovin-39-OH
**31.** Tenovin-50
**32.** Tenovin-50-OH
**34.** Chloroquine
**33.** Tenovin-D3
**35.** DMSO

Fig 2B – 24 hour treatment blot (LC3B left blot, p62 right blot)

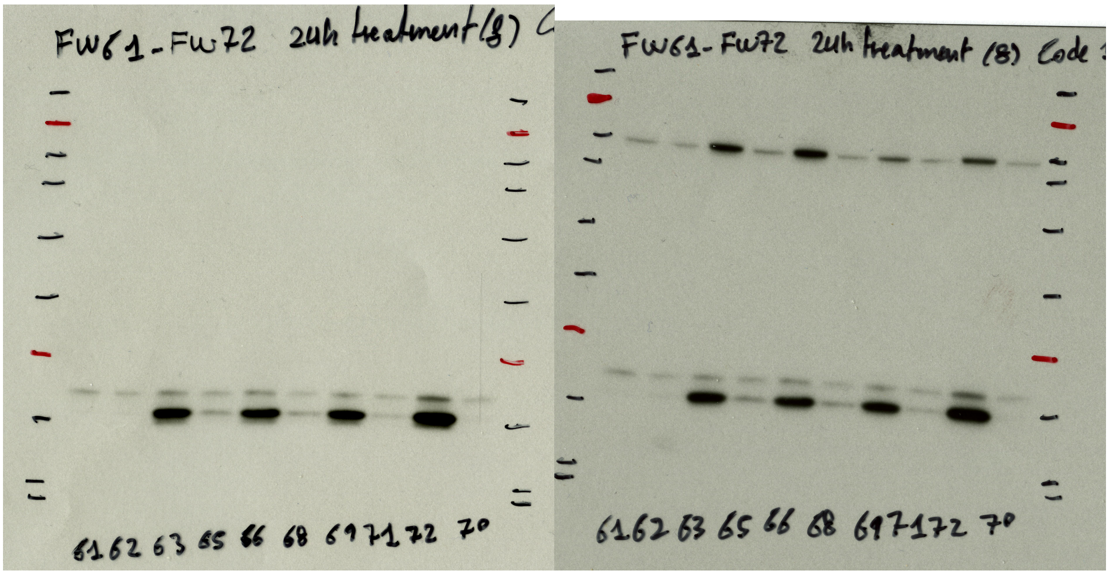

gapdh blot

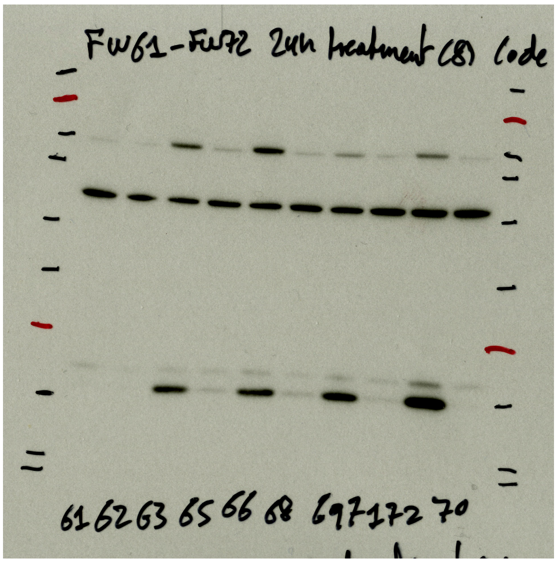

**Samples**
**61.** DMSO
**62.** Tenovin-1
**63.** Tenovin-6
**65.** Tenovin-33
**66.** Tenovin-39
**68.** Tenovin-39-OH
**69.** Tenovin-50
**71.** Tenovin-50-OH
**72.** Chloroquine
**70.** DMSO

Fig 2C – Chloroquine with LC3B

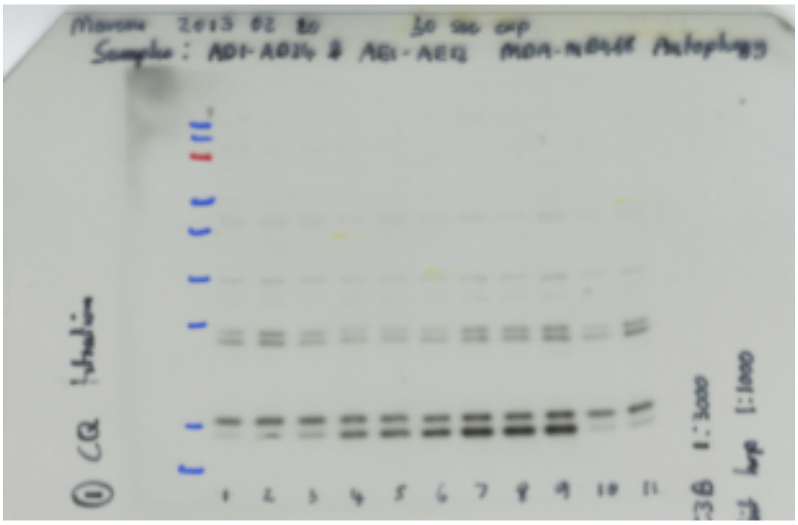

Chloroquine with alpha tubulin

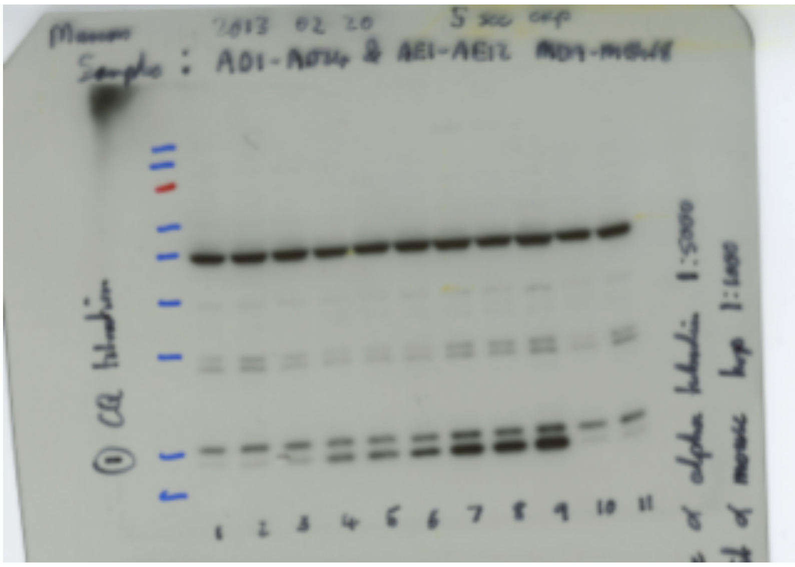

**Top Blot**
**1.** PBS
**2.** PBS
**3.** 2.5 µM chloroquine
**4.** 5 µM chloroquine
**5.** 10 µM chloroquine
**6.** 15 µM chloroquine
**7.** 25 µM chloroquine
**8.** 50 µM chloroquine
**9.** 100 µM chloroquine
**10.** PBS
**11.** PBS

Fig 2C Tenovin 39 and 39OH +/- Chloroquine LC3B blot.

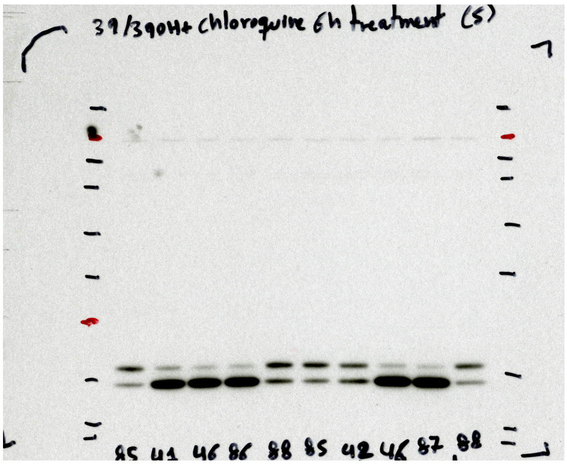

gapdh blot

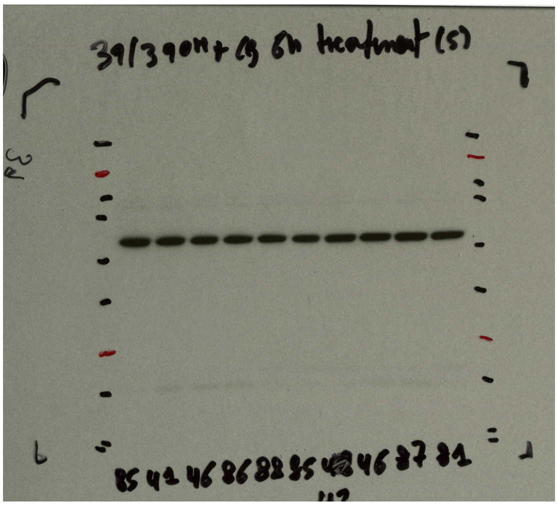

**Samples**
**85.** DMSO
**41.** Tenovin 39
**46.** Chloroquine (CQ)
**86.** Tenovin 39 + CQ
**88.** DMSO
**85.** DMSO
**42.** Tenovin-39-OH
**46.** CQ
**87.** Tenovin-39-OH + CQ
**88.** DMSO

Fig 2C Tenovin 50/50OH +/- Chloroquine LC3B blot

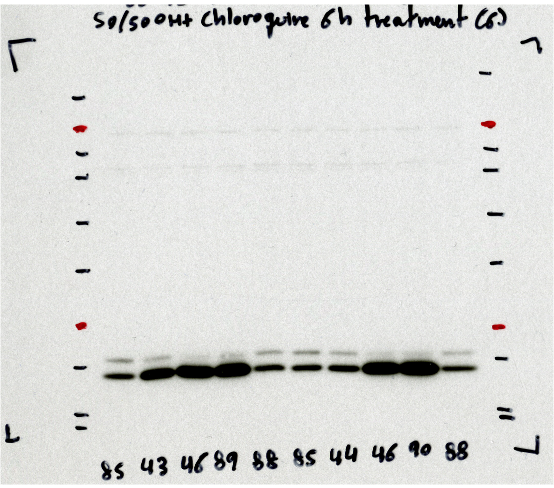

gapdh blot

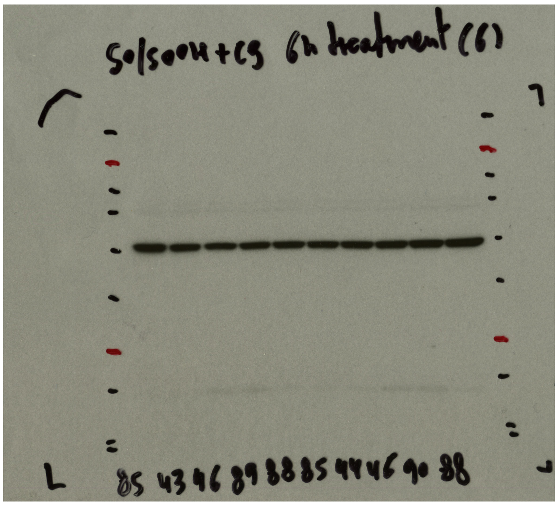

**Samples**
**85.** DMSO
**43.** Tenovin 50
**46.** Chloroquine (CQ)
**89.** Tenovin 50 + CQ
**88.** DMSO
**85.** DMSO
**44.** Tenovin-50-OH
**46.** CQ
**90.** Tenovin-50-OH + CQ
**88.** DMSO

Fig 3 – ARN8 blots (top panel) (LC3B left blot and alpha tubulin right blot).

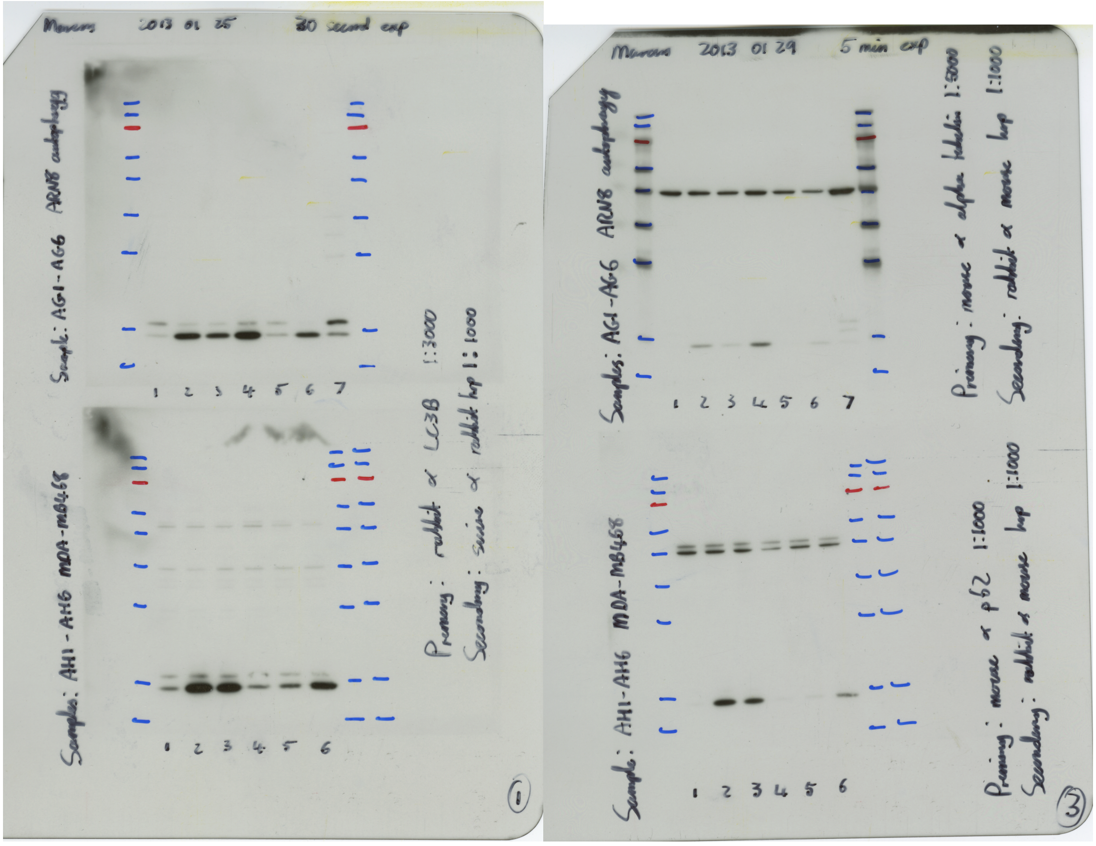

**Top Blot**
**1.** DMSO
**2.** 100 µM chloroquine
**3.** 15 µM tenovin 6
**4.** 15 µM tenovin 50
**5.** 20 µM tenovin 50 OH
**6.** DMSO
**7.** Separate experiment

S2 Fig – HNDF LC3B (left blot) and alpha tubulin (right blot)

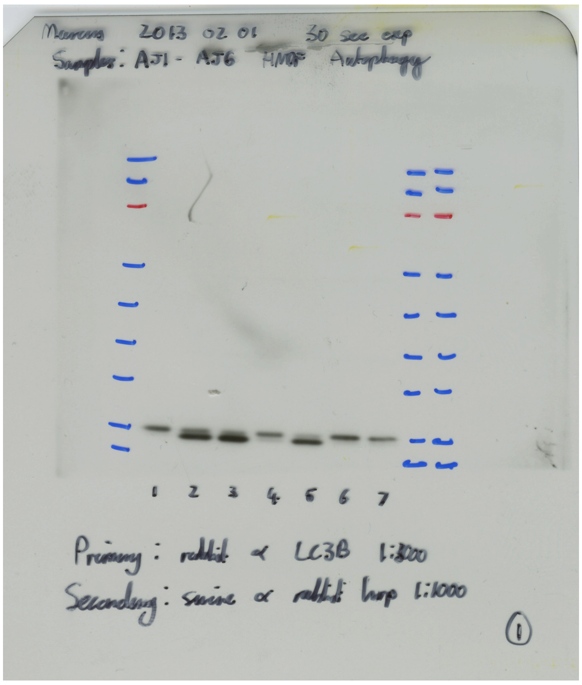

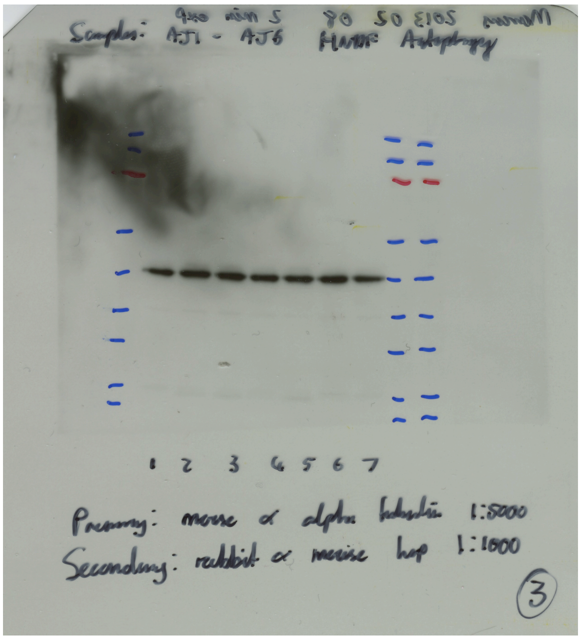

**Top Blot**
**1.** DMSO
**2.** 100 µM chloroquine
**3.** 15 µM tenovin 50
**4.** 15 µM tenovin 50 OH
**5.** 15 µM tenovin 39
**6.** DMSO
**7.** 15 µM tenovin 39 OH

S4 Fig - 1h treatment blot (LC3B left blot, p62 right blot)

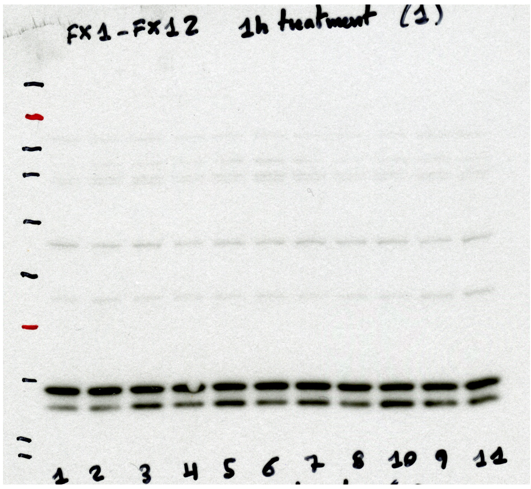

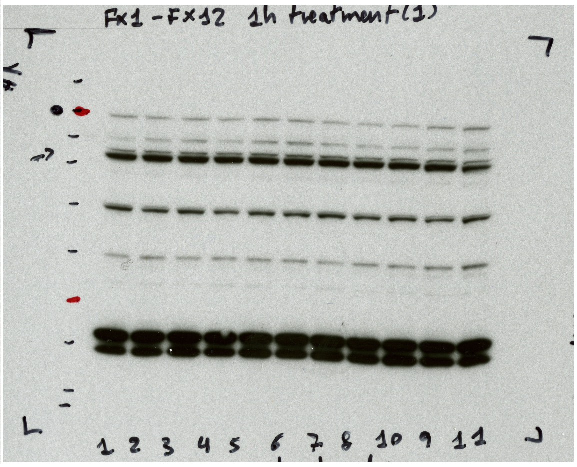

gapdh blot

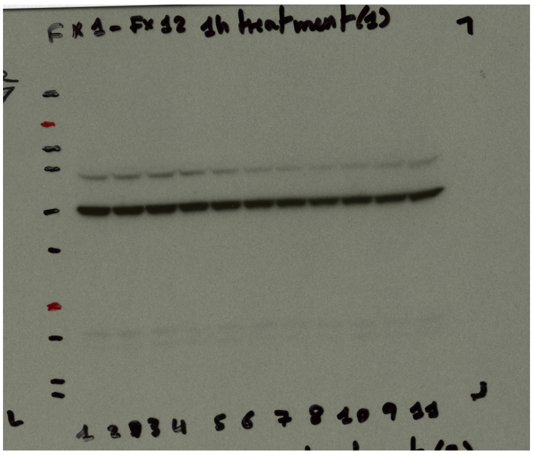

**Samples**
**1.** DMSO
**2.** Tenovin-1
**3.** Tenovin-6
**4.** Tenovin-33
**5.** Tenovin-39
**6.** Tenovin-39-OH
**7.** Tenovin-50
**8.** Tenovin-50-OH
**10.** Chloroquine
**9.** Tenovin-D3
**11.** DMSO

S4 Fig – 2 hour treatment blot (LC3B left blot and p62 right blot).

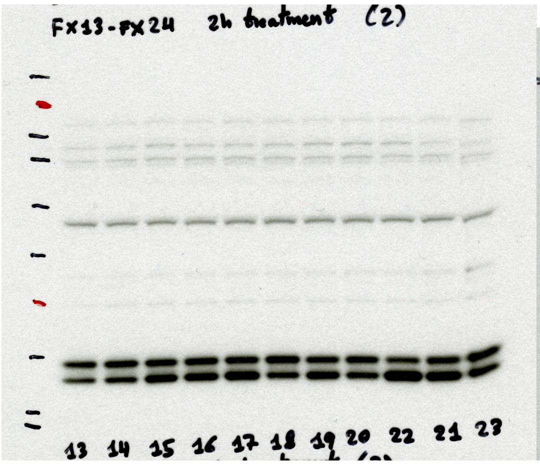

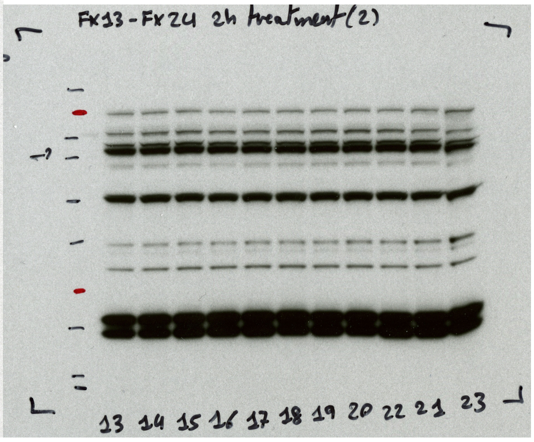

gapdh blot

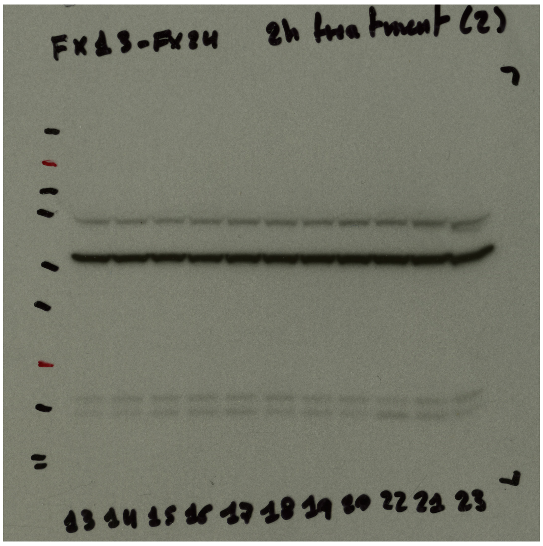

**Samples**
**13.** DMSO
**14.** Tenovin-1
**15.** Tenovin-6
**16.** Tenovin-33
**17.** Tenovin-39
**18.** Tenovin-39-OH
**19.** Tenovin-50
**20.** Tenovin-50-OH
**22.** Chloroquine
**21.** Tenovin-D3
**23.** DMSO

S4 Fig – 4 hour treatment (LC3B left blot, p62 right blot).

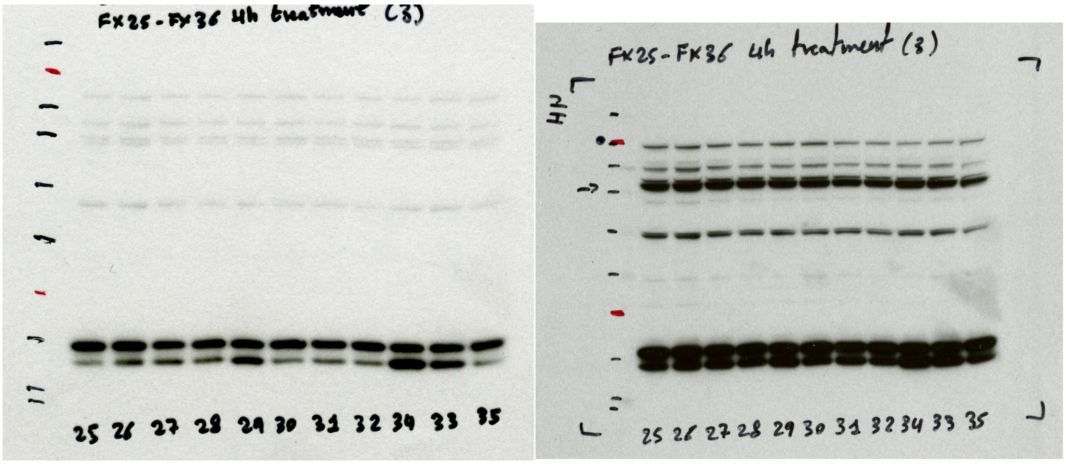

gapdh blot

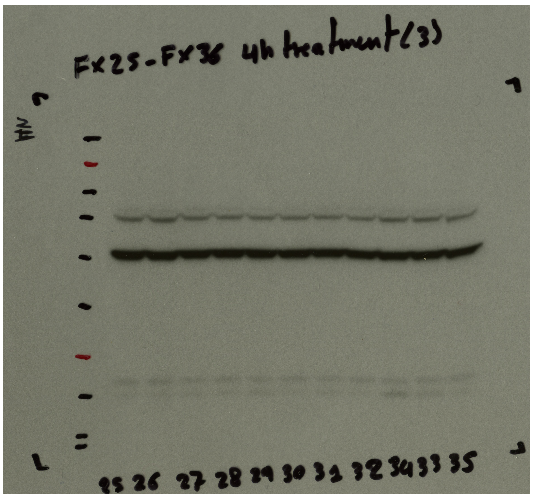

**Samples**
**25.** DMSO
**26.** Tenovin-1
**27.** Tenovin-6
**28.** Tenovin-33
**29.** Tenovin-39
**30.** Tenovin-39-OH
**31.** Tenovin-50
**32.** Tenovin-50-OH
**34.** Chloroquine
**33.** Tenovin-D3
**35.** DMSO

S4 Blot – 6 hour treatment (LC3B left blot, p62 right blot).

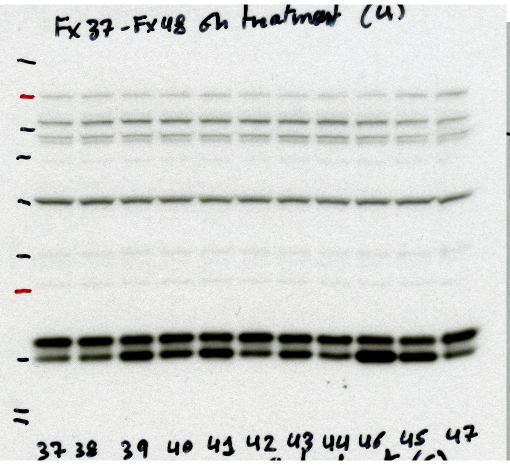

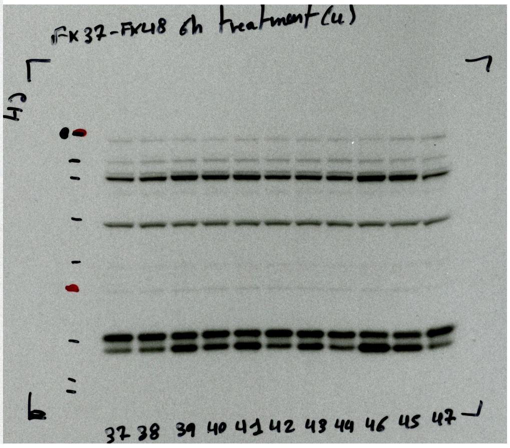

gapdh blot

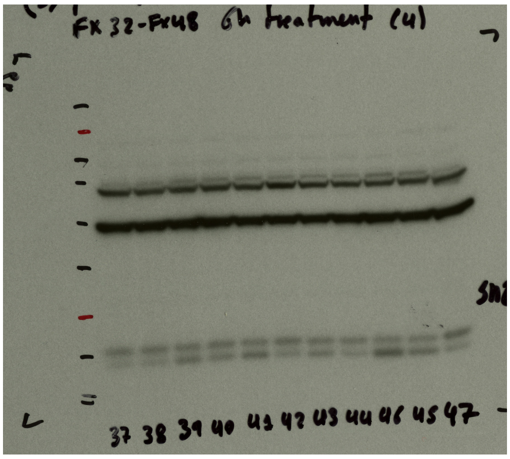

**Samples**
**37.** DMSO
**38.** Tenovin-1
**39.** Tenovin-6
**40.** Tenovin-33
**41.** Tenovin-39
**42.** Tenovin-39-OH
**43.** Tenovin-50
**44.** Tenovin-50-OH
**46.** Chloroquine
**45.** Tenovin-D3
**47.** DMSO

S4 Fig – Tenovin 39/39OH +/- chloroquine (LC3B blot left, p62 blot right).

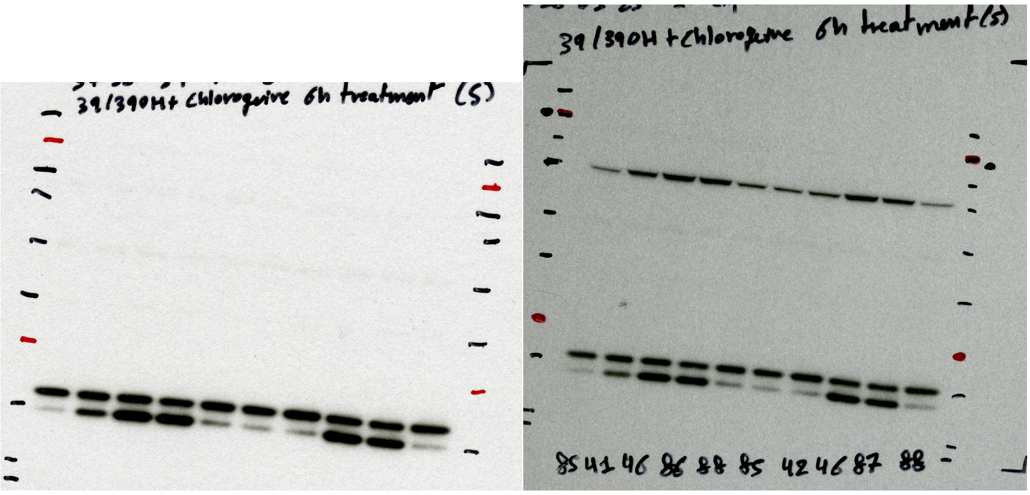

gapdh blot

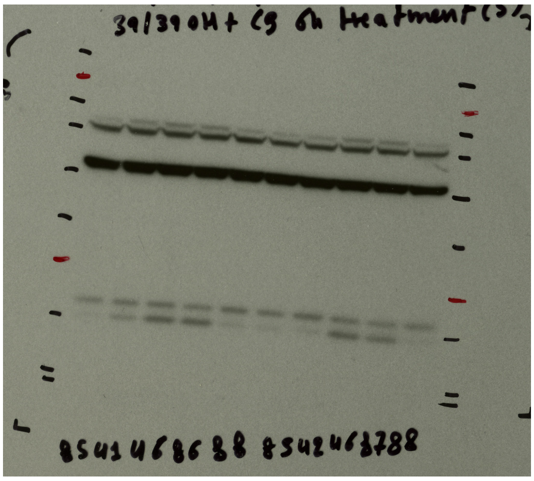

**Samples**
**85.** DMSO
**41.** Tenovin 39
**46.** Chloroquine (CQ)
**86.** Tenovin 39 + CQ
**88.** DMSO
**85.** DMSO
**42.** Tenovin-39-OH
**46.** CQ
**87.** Tenovin-39-OH + CQ
**88.** DMSO

S4 Fig – Tenovin 50/50OH +/- chloroquine (LC3B left blot, p62 right blot).

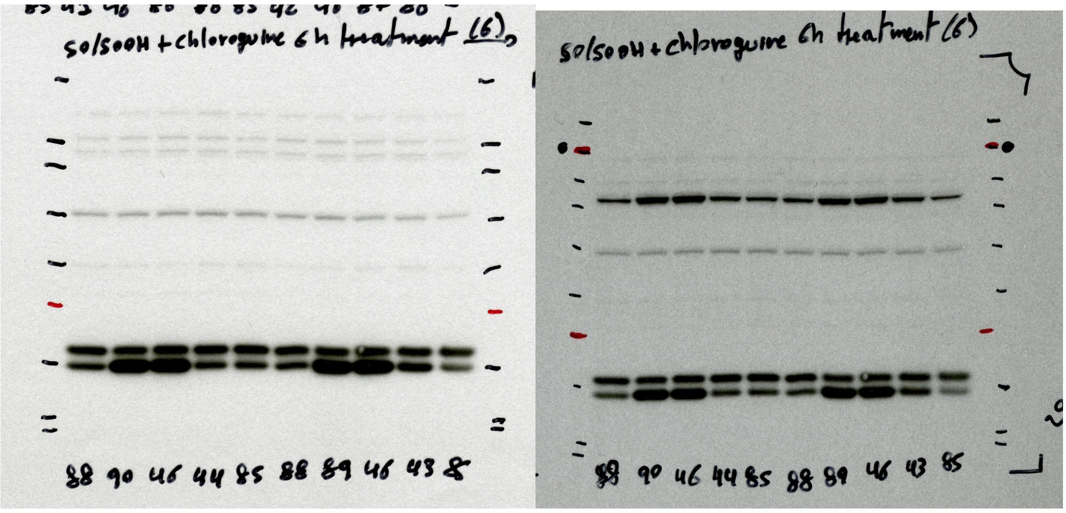

gapdh blot

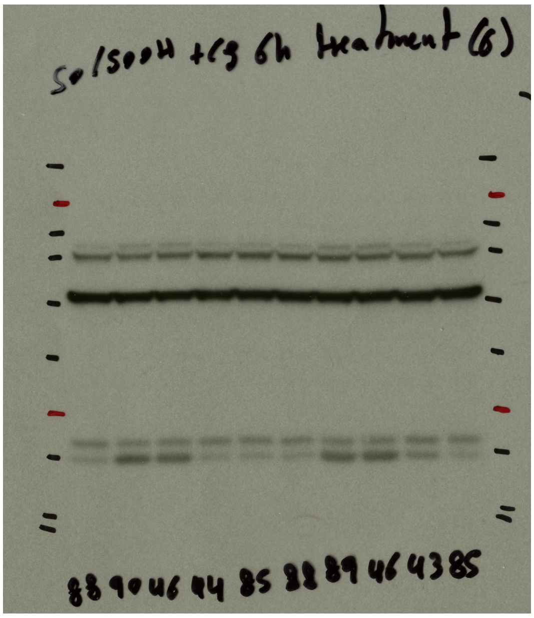

**Samples**
**88.** DMSO
**90.** Tenovin-50-OH + CQ
**46.** CQ
**44.** Tenovin-50-OH
**85.** DMSO
**88.** DMSO
**89.** Tenovin 50 + CQ
**46.** Chloroquine (CQ)
**43.** Tenovin 50
**85.** DMSO

All blots for supplemental figure 2 are available electronically as a ZIP file and can be opened using the free software available from BioRad (ImageLab).
